# Sumoylation stabilizes RACK1B and enhance its interaction with RAP2.6 in the abscisic acid response

**DOI:** 10.1038/srep44090

**Published:** 2017-03-08

**Authors:** Rongkai Guo, Weining Sun

**Affiliations:** 1 Shanghai Institute of Plant Physiology and Ecology, Chinese Academy of Sciences, Shanghai 200032, China; 2 University of Chinese Academy of Sciences, Beijing 100039, China

## Abstract

The highly conserved eukaryotic WD40 repeat protein, Receptor for Activated C Kinase 1 (RACK1), is involved in the abscisic acid (ABA) response in *Arabidopsis*. However, the regulation of RACK1 and the proteins with which it interacts are poorly understood. Here, we show that RACK1B is sumoylated at four residues, Lys50, Lys276, Lys281 and Lys291. Sumoylation increases RACK1B stability and its tolerance to ubiquitination-mediated degradation in ABA response. As a result, sumoylation leads to enhanced interaction between RACK1B and RAP2.6, an AP2/ERF family transcription factor. RACK1B binds directly to the AP2 domain of RAP2.6, which alters the affinity of RAP2.6 for CE1 and GCC *cis*-acting regulatory elements. Taken together, our findings illustrate that protein stability controlled by dynamic post-transcriptional modification is a critical regulatory mechanism for RACK1B, which functions as scaffold protein for RAP2.6 in ABA signaling.

Protein sumoylation, characterized by the covalent conjugation of a small ubiquitin-related modifier (SUMO) protein to a lysine residue in a target protein, is a key post-translational modification in plants. Eight SUMO isoforms, SUMO1–8, are found in *Arabidopsis*, of which SUMO1/2 is the main conjugate^1^. Similar to ubiquitination, sumoylation requires an E1-E2-E3 enzyme cascade to catalyze the activation, conjugation and ligation processes^2^,^3^. The E1, a heterodimer, activates the SUMO by thioester formation^4^. In *Arabidopsis*, two redundant genes, *SAE1A* and *SAE1B*, encode the smaller subunit of E1, whereas the larger subunit is encoded by a single gene, SAE2^4^. The E2, encoded by *AtSCE1*, transfers the SUMO from E1 to the lysine site of the substrate^4^. In some cases, the E2 is able to directly recognize not only the lysine located in the conical consensus sequence (ΨKxE) but also other irregular lysine residues^5^. The E3 ligase functions as an additional platform that promotes the interaction between SUMO-charged E2 and targets^6^. SIZ1 and HPY2 were identified as functional E3 ligases for protein sumoylation in *Arabidopsis*^7^,^8^. Recently, two E4-type SUMO ligases, PIAL1 and PIAL2, were identified that catalyze SUMO chain formation^9^.

A wide range of environmental and developmental signals, such as abiotic stresses^7^,^10^,^11^, the innate immune response^12^, hormones^13^, flowering^4^, and meristem maintenance^8^, are coupled with massive and rapid changes of sumoylation in *Arabidopsis*. In the abscisic acid (ABA) response, sumoylation of ABI5 by SIZ1 increases its stability and negatively regulates seed germination and inhibits primary root growth^14^. Sumoylation of MYB30, which functions in parallel with the ABI5-mediated ABA signaling pathway during seed germination^15^, is also stabilized by SIZ1. These results indicate that sumoylation is a regulatory mechanism in ABA responses.

The modified stability of transcription factors is often concomitant with altered transcriptional activities. Several *cis*-regulatory elements are involved in ABA signaling, such as the ABA responsive element (ABRE)^16^, the coupling element (CE)^17^,^18^, MYC recognition sites (MYCRs) and MYB recognition sites (MYBRs)^19^,^20^ and NAC recognition sites (NACRs)^21^. Among them, the ABRE is the best characterized *cis*-acting regulatory element and is recognized by the ABRE-binding protein/ABRE binding factor (AREB/ABF) in ABA-dependent gene expression^22^,^23^,^24^,^25^. However, a single ABRE copy is not sufficient for ABA-induced gene transcription and coupling elements, such as CE1 and CE3, are required for an effective ABA-responsive complex^17^,^18^. Using gel shift assays and yeast one-hybrid screenings, a group of proteins which could interact with CE1 or a CE1-like element were identified and designated as CE1 binding proteins (CEBPs)^26^. These proteins belong to the APETALA2/Ethylene Response Factor (AP2/ERF) transcription factor family^26^. In addition, most CEBPs can interact with the GCC-box, which acts as a *cis*-acting regulatory element in ethylene and jasmonic acid responses^27^,^28^.

Receptor for Activated C Kinase 1 (RACK1) functions as a versatile scaffold protein in all well-studied eukaryotic organisms^29^. The structure of RACK1 is characterized as a seven-bladed β-propeller, which provides a platform for protein-protein interaction in both mammalian and plant cells^30^,^31^. RACK1 is a ribosomal protein but it possesses no known enzymatic activity^32^. In the *Arabidopsis* genome, there are three homologous *RACK1* genes, *RACK1A, RACK1B* and *RACK1C*^33^. Accumulating evidence indicates that RACK1 plays pivotal roles in diverse physiological processes in plants. RACK1 regulates plant developmental processes, including rosette leaf production and root development^34^. RACK1 also mediates responsiveness to multiple phytohormones, such as brassinosteroid, gibberellins and abscisic acid, and functions in disease resistance and the innate immune pathway^33^. In addition, RACK1 is involved in several abiotic stresses, for example, water stress^31^.

It is well-known that *Arabidopsis* RACK1 participates in ABA signaling. Based on the observation that a *rack1a* single mutant, and *rack1a/rack1b* or *rack1a/rack1c* double mutants are hypersensitive to the inhibition effect of ABA in seed germination, cotyledon greening and root growth, it was proposed that AtRACK1 functioned as a negative regulator of the ABA response^35^. RACK1 affected the accumulation and processing of pri-miR159, which targets TF MYB33 and MYB65 in ABA responses^36^. On the other hand, ABA down-regulated the expression of *RACK1* and *eIF6*, which is a regulator for 80S ribosome assembly in mammalian cells^37^. This supports the finding that ABA impaires biogenesis of 60S ribosome subunit and ribosome assembly, although ABA had no effect on the RACK1-eIF6 interaction^37^. These studies indicate that *Arabidopsis* RACK1 might be involved in an ABA-regulated feedback loop. Although RACK1s play important roles in the ABA response, little is known about their up- and downstream interacting partners or the roles they play in regulating the ABA response.

Here, we report that *Arabidopsis* RACK1B is sumoylated at four residues, K50, K276, K281 and K291. Sumoylation increases the stability of RACK1B by blocking Ub-conjugation. Increased levels of exogenous ABA interfere with the balance between sumoylation and ubiquitination of RACK1B and pushes the balance towards sumoylation, thereby enhancing RACK1B stability. In addition, we provided evidence that ABA-stimulated sumoylation increased the interaction between RACK1B and transcription factor RAP2.6, which is a member of AP2/ERF family. This interaction resulted in altered affinity of RAP2.6 for CE1 and the GCC-box, which are important responsive elements in ABA and ethylene/jasmonic acid signaling, respectively^17^,^28^,^38^.

## Results

### RACK1B is subjected to sumoylation

**Figure 1:**
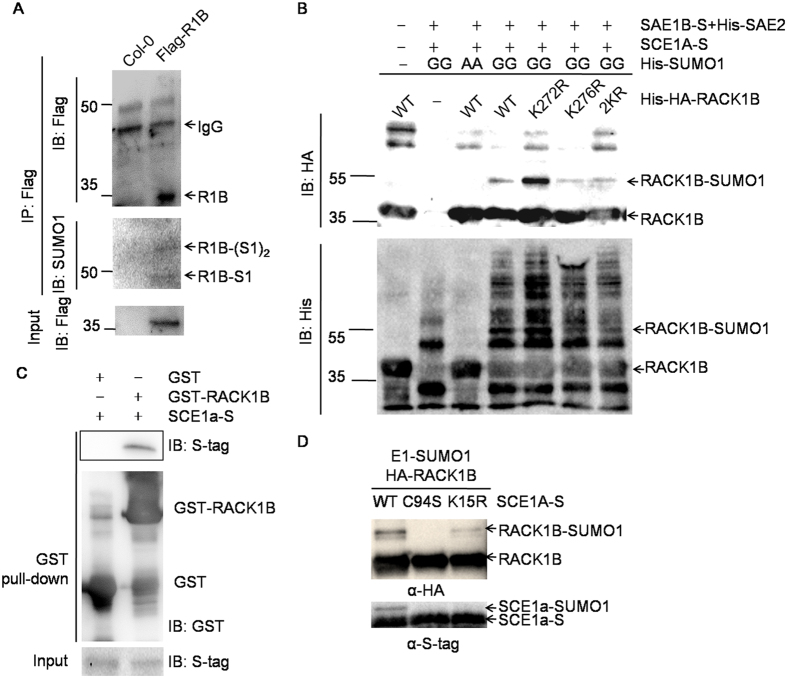
RACK1B is sumoylated by SUMO1. (**A**) *in vivo* sumoylation assay. Seven-day-old Col-0 and *35S::Flag-RACK1B/rack1b-2* seedlings were used. Equal amounts of individual proteins were immunoprecipitated with anti-Myc antibodies and sumoylation was detected with the indicated antibodies. (**B**) *In vitro* sumoylation assay for RACK1B. Sumoylation of HA-RACK1B and K272R, K276R or K272R/K276R (2KR) mutants was detected by immunoblotting with anti-HA (upper panel) and anti-His antibodies (lower panel), respectively. GG and AA stand for WT forms of SUMO1 and SUMO1^G92A-G93A^, respectively. (**C**) GST pull-down analysis of interaction between SCE1A and RACK1B. GST-SCE1A or GST was used as bait to pull-down HA-RACK1B recombinant protein. Target proteins were eluted with 2 × sampling buffer and detected by immunoblot analysis with the antibodies indicated. (**D**) *In vitro* sumoylation assay with SCE1A mutants. S-tag fused SCE1A^C94S^ or SCE1A^K15R^ were used in the *E. coli* sumoylation system and compared with WT SCE1A. The sumoylation of HA-RACK1B was detected by immunoblot with anti-HA antibodies. SCE1A and SAE1B were detected with anti-S-tag antibodies.

By analyzing protein structures and sequence homology of RACK1 from *Arabidopsis* and other species, two lysine residues, K272 and K276, were predicted to be potential sumoylation sites in AtRACK1B31. Further analysis with SUMOsp 2.0 software^39^ showed that both K276 and K272 have high probability as sumoylation sites and represent consensus and non-consensus sequences for sumoylation, respectively. To test whether RACK1B could be sumoylated, stable transgenic line expressing *Flag-RACK1B* in *rack1b-2* background was used. The Flag tagged RACK1B was enriched by immunoprecipitation. Sumoylated RACK1B was detected by immunoblotting with an anti-Myc antibody (Fig. 1A). The identity of the protein corresponding to the immunoreactive signal at 50 kD was further confirmed to be SUMO1-conjugated RACK1B by tandem liquid chromatograph-mass spectrometry (LC-MS/MS) (Supplementary Table S1). These results suggested that RACK1B is indeed sumoylated by SUMO1 *in vivo*.

Protein sumoylation can be accomplished either in an E3-dependent or an E3-independent manner^40^. To understand the RACK1B sumoylation mode and to verify the sumoylation site(s), an *in vitro* system consisting of E1, E2, SUMO and target protein was reconstituted in *E. coli*. The SUMO pathway-related proteins, SAE1B, SAE2, SCE1A and SUMO1(GG) were co-expressed with HA-RACK1B in *E. coli*. Note that SUMO1(GG) was used to mimic the mature form of SUMO1, which has C-terminal diglycine residues. A mutated version of SUMO1 containing two Ala residues [SUMO1(AA)], which cannot form a thioester bond, was used as a negative control^6^,^41^. In agreement with the *in vivo* experiment, both RACK1B and SUMO1-conjugated RACK1B could be detected (Fig. 1B). To our surprise, sumoylated RACK1B could still be detected in K272R and K276R single mutants and in the K272/K276R double mutant, suggesting that other undiscovered sumoylation sites may exist (Fig. 1B). Furthermore, RACK1B was not subjected to SUMO3 or SUMO5 conjugation *in vitro*, suggesting that RACK1B is specifically sumoylated by SUMO1 (Supplementary Fig. 1).

The fact that RACK1B can be sumoylated *in vitro* without the help of an E3 ligase indicates that SCE1A (E2) is able to directly recognize RACK1B. To test the hypothesis that SCE1A and RACK1B are components of one complex, we performed pull-down assays. The results showed that SCE1 directly interacted with RACK1B (Fig. 1C). SCE1A is self-sumoylated at Lys15 and at the catalytic Cys94 residue (within the active center)^9^; therefore, we investigated whether sumoylation of SCE1A is a prerequisite for sumoylation of RACK1B. Cys94 and Lys15 were mutated to Ser and Arg, respectively, via site-directed mutagenesis. As shown in Fig. 1D, RACK1B could be sumoylated by SCE1A^K15R^ but not by the negative control SCE1A^C94S^, which suggested that SCE1A sumoylation at Lys15 was not necessary for RACK1B sumoylation. We also tested whether the two known E3 ligases contribute to the RACK1B sumoylation. The SIZ1 could interact with RACK1B and increased concentration of SIZ1 resulted in increased conjugation of SUMO1 on RACK1B *in vitro* (Supplementary Fig. 2A,B). However, sumoylated RACK1B remained detectable in the *siz1–2* mutant (Supplementary Fig. 2C), suggesting that sumoylation of RACK1B is facilitated by but not rely on SIZ1. For the other E3 ligase, HPY2, due to severe dwarfism and defective meristems^8^, we failed to overexpress *RACK1B* in the *hpy2–2* mutant. Therefore, a co-immunoprecipitation assay was performed to examine the interaction between RACK1B and HPY2. HPY2 was not precipitated with RACK1B and could not facilitate its sumoylation (Supplementary Fig. 2D–E), suggesting that HPY2 might not be involved in the RACK1B sumoylation complex. Taken together, these results suggested that RACK1B can be sumoylated via direct interaction between SCE1A and RACK1B.

### RACK1B contains four lysine receptors of SUMO1

**Figure 2:**
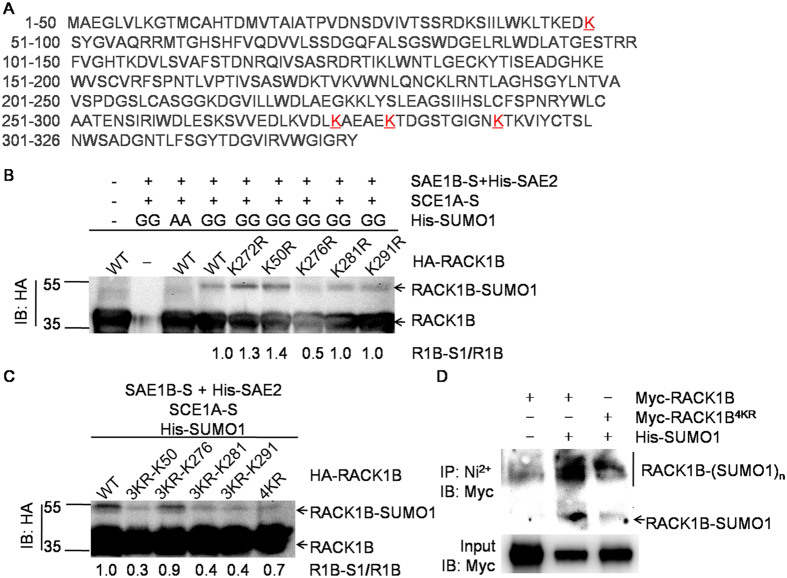
RACK1B has four major lysine residues. (**A**) Amino acid sequence of RACK1B. Four lysine residues that could be sumoylated are underlined. (**B**,**C**) *In vitro* sumoylation assay. The sumoylation of RACK1B and its mutants was examined in the SUMO1-sumoylation system and was detected by immunoblotting with anti-HA antibodies. In (**C**), 3KR-K50, RACK1B^K276,281,291R^; 3KR-K276, RACK1B^K50,281,291R^; 3KR-K281, RACK1B^K50,276,291R^; 3KR-K291, RACK1B^K50,276,281R^; 4KR, RACK1B^K50,276,281,291R^. (**D**) *In vivo* sumoylation assay. *35S::Myc-RACK1B* or *35S::Myc-RACK1B*^*4KR*^was co-transformed with *35S::His-SUMO1(GG*) or empty vector in Col-0 protoplasts. The purified proteins were separated by SDS-PAGE and detected by immunoblotting with anti-Myc antibodies.

The sumoylation site(s) of RACK1B were identified using LC-MS/MS. In the *E. coli* sumoylation system, SUMO1(GG) was replaced by SUMO1 H89R(GG), which leaves a QTGG footprint on receptor Lys residues after digestion with trypsin^42^,^43^. The QTGG footprint facilitates the identification of sumoylated peptides via mapping the paralog-specific fragment ions. The sumoylated RACK1B was purified, separated and subjected to LC-MS/MS analysis (Supplementary Fig. 3). Four Lys residues, Lys50, Lys276, Lys281 and Lys291, conjugated to the QTGG footprint, were identified (Fig. 2A, Supplementary Fig. 4 and Supplementary Table S2). In addition to the consensus Lys276, Lys50 was located in a reverse consensus sequence (D/ExKΨ), and the other two Lys residues were considered as non-consensus sites. To our surprise, Lys272 was not sumoylated.

The identified Lys receptors were then verified by immunoblot analysis. Five single mutants HA-RACK1B (K272R, K50R, K276R, K281R or K291R), four triple mutants [K276,281,291R (3KR-K50), K50,281,291R (3KR-K276), K50,276,291R (3KR-K281), K50,276,281R (3KR-K291)], and a quadruple mutant [K50,276,281,291R (4KR)] were constructed and expressed in the *E. coli* sumoylation system. The sumoylation level of RACK1B^K276R^ was severely decreased compared to the other four single mutants, while the sumoylation level of RACK1B^3KR–K276^ remained the highest among the four triple mutants (Fig. 2B,C), suggesting that SCE1A preferentially binds to the consensus Lys276 site *in vitro*. Mutation of the non-sumoylation site, K272R, increased the sumoylation level of RACK1B. The K50R mutation also slightly increased the level of sumoylation. Mutation of all four lysine residues severely decreased the sumoylation level both *in vivo* and *in vitro* although very low levels of sumoylation remained detectable (Fig. 2C,D). Taken together, these results indicated that RACK1B contains four sumoylation residues, Lys50, Lys276, Lys281 and Lys291.

### Sumoylation increases the stability of RACK1B

**Figure 3:**
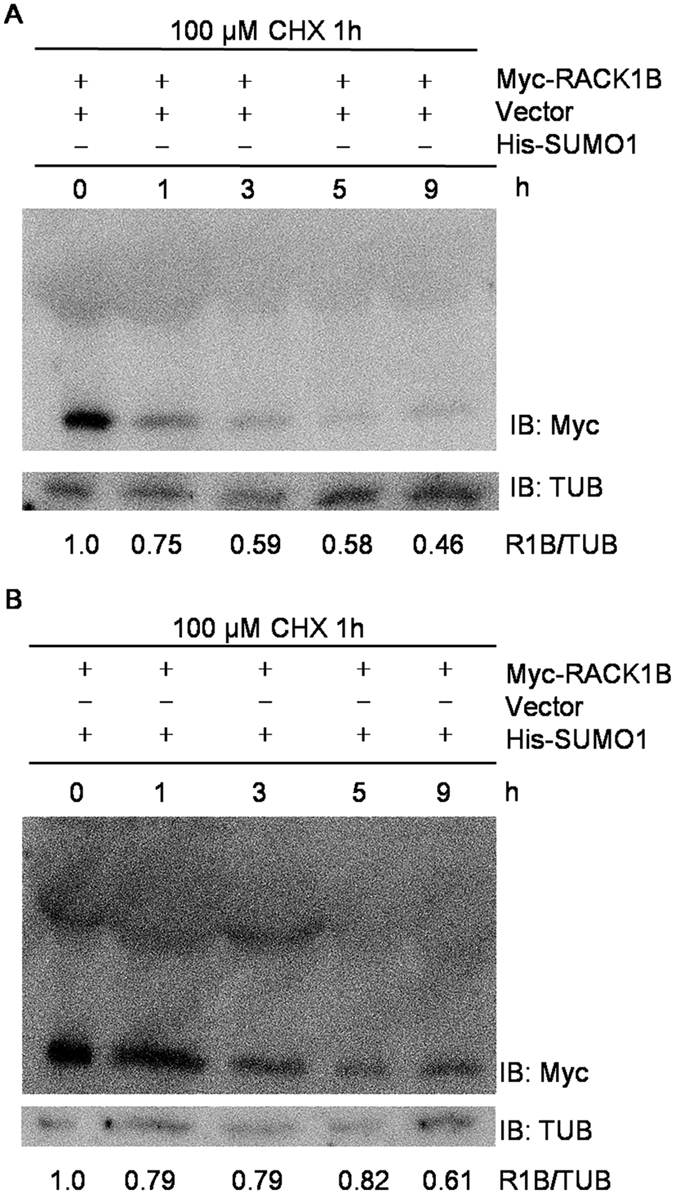
Sumoylation increases the stability of RACK1B. (**A**,**B**) Degradation assays. *35S::Myc-RACK1B* co-transformed with *35S::His-SUMO1(GG*) (**A**) or empty vector (**B**) in Col-0 protoplasts. After incubation for 20 h, the protoplasts were treated with 100 μM CHX and samples were harvested at 0, 1, 3, 5 and 9 h. The degradation of Myc-RACK1B was detected by immunoblotting with anti-Myc antibodies. Tubulin was used as a loading control. The signal intensities were analyzed and quantified using ImageJ. The Myc-RACK1B signal was normalized against that of tubulin and compared to signal at t = 0, which was set as 1.0. The numbers underneath represent the relative Myc-RACK1B abundance at each time point.

One consequence of sumoylation is a change in protein turnover rate^5^; therefore, we explored whether sumoylation influenced the stability of RACK1B. The degradation rates between transiently expressed *RACK1B* co-expressed with and without *SUMO1(GG*) were compared after treatment with cycloheximide (CHX), which inhibits new protein synthesis. As shown in Fig. 3, the level of RACK1B was significantly decreased at 3 h and was almost vanished at 5 h after CHX treatment in protoplasts expressing wild-type RACK1B alone. Co-transformation of *SUMO1(GG*) with *RACK1B* retarded the degradation of RACK1B and it remained detectable even 9 h after CHX treatment. Together, these results supported our postulation that sumoylation increases the stability of RACK1B. Note that we did not verify the degradation of RACK1B^4KR^ as a negative control because mutation of these four lysine residues also reduced the level of ubiquitination (see below).

### Sumoylation of RACK1B competes with its ubiquitination

**Figure 4:**
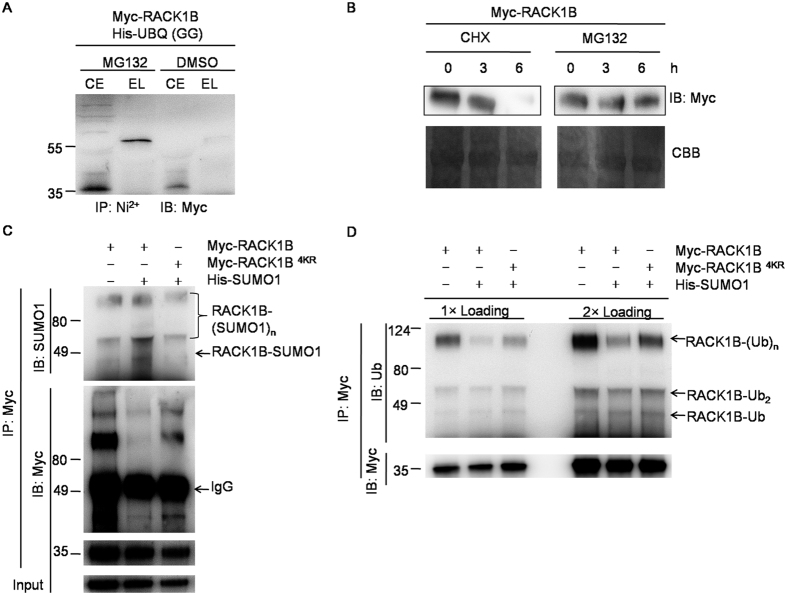
Sumoylation of RACK1B competes with ubiquitination at the same lysine residues. (**A**) *In vivo* ubiquitination assay. *35S::Myc*-*RACK1B* was co-transformed with *35S::His-UBQ(GG*) in Col-0 protoplasts and treated with 50 μM MG132 or DMSO for 20 h. The ubiquitination of RACK1B was detected by immunoblotting with anti-Myc antibodies. CE, crude extract, EL, elutes from Ni^2+^-chromatography. (**B**) Degradation assay. *35S::Myc-RACK1B* was transiently expressed in Col-0 protoplasts. After incubation for 20 h, the protoplasts were treated with 100 μM CHX or 50 μM MG132. The degradation rates of RACK1B were compared at the indicated time points. (**C**,**D**) *In vivo* immunoprecipitation assay. *35S::Myc-RACK1B* or *35S::Myc-RACK1B*^*4KR*^was expressed with or without *35S::His-SUMO1(GG*) in Col-0 protoplasts. RACK1B and RACK1B^4KR^ were precipitated using anti-Myc antibodies and subjected to immunoblot analysis with anti-SUMO1 (**C**) or anti-ubiquitin (**D**) antibodies.

Sumoylation of RACK1B increased its stability; therefore, we asked whether sumoylation prevents the degradation of RACK1B. First, we hypothesized that RACK1B is degraded by ubiquitination-mediated degradation. To confirm this, *RACK1B* and *UBQ(GG*) were co-expressed in *Arabidopsis* protoplasts. As shown in Fig. 4A, ubiquitinated RACK1B could only be detected after purification via Ni^2+^-chromatography with the addition of the proteasome inhibitor MG132 (Selleck, Shanghai, China). The unmodified form of RACK1B (35 kDa) was protected by MG132 (Fig. 4B). In contrast, over 95% of RACK1B was degraded after 6 h without MG132. These results indicated that RACK1B is degraded through the ubiquitination-mediated 26S proteosomal degradation pathway.

To confirm whether sumoylation prevents the degradation of RACK1B by competing with ubiquitination, *RACK1B* or *RACK1B*^*4KR*^was transformed into protoplasts with *SUMO1(GG*). Overexpression of *SUMO1(GG*) increased the sumoylation level of RACK1B but not that of RACK1B^4KR^ (Fig. 4C). Interestingly, the level of ubiquitinated RACK1B was significantly decreased in protoplasts overexpressing *SUMO1(GG*), whereas the level of ubiquitinated RACK1B^4KR^ was slightly reduced and but still higher than that of RACK1B (Fig. 4D). This result suggested that other unidentified lysine receptors might also be potential ubiquitination sites, and the competition exists between sumoylation and ubiquitination of RACK1B.

### ABA increases RACK1B stability by facilitating sumoylation

**Figure 5:**
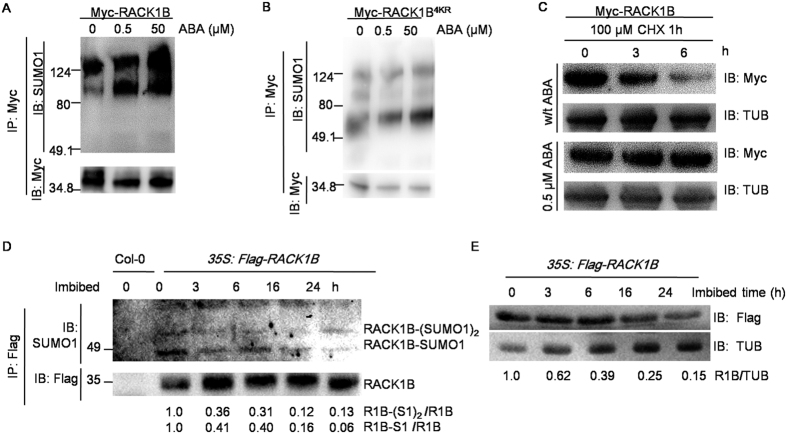
Sumoylation of RACK1B is regulated by ABA. (**A**,**B**) *In vivo* sumoylation assay. *35S::Myc-RACK1B* (**A**) and *35S::Myc-RACK1B*^*4KR*^ (**B**) were expressed in Col-0 protoplasts and treated with 0, 0.5 and 50 μM ABA for 20 h. Myc-RACK1B and Myc-RACK1B^4KR^ were immunoprecipitated with anti-Myc antibodies. Protein sumoylation levels were detected by immunoblotting with anti-SUMO1 antibodies. (**C**) *In vivo* degradation assay. *35S::Myc-RACK1B* or *35S::Myc-RACK1B*^*4KR*^ was expressed in Col-0 protoplasts and treated with or without 0.5 μM ABA for 20 h before 100 μM CHX treatment for 0, 3 and 6 h. Degradation of Myc-RACK1B was detected by immunoblotting with anti-Myc antibodies. Tubulin was used as a loading control. (**D**) The level of Flag-RACK1B in imbibed transgenic *35S::Flag-RACK1B* seeds. The protein level of Flag-RACK1B was detected by immunoblotting with anti-Flag antibodies. (**E**) *In vivo* sumoylation assay. Individual proteins from (**D**) were subjected to immunoprecipitation using anti-Flag antibody-conjugated beads. The proteins were eluted and detected by immunoblotting with anti-SUMO1 and anti-Flag antibodies. In (**D**,**E**) the signal intensities were quantified using ImageJ. The numbers underneath represent the relative protein abundance at each time point, normalized against tubulin.

RACK1B is involved in the ABA signaling pathway^35^; therefore, we asked whether ABA regulates RACK1B at a post-translational level via sumoylation. Protoplasts expressing RACK1B were treated with different concentrations of ABA and, as expected, the sumoylation level of RACK1B significantly increased in a dose-dependent manner (Fig. 5A). In addition, the sumoylation level of Myc-RACK1B^4KR^ was not affected by ABA, although a background of sumoylation still existed (Fig. 5B). Next, the degradation rates of RACK1B in protoplasts treated with exogenous ABA and in mock protoplasts were compared. The protoplasts were first treated with CHX to stop new protein synthesis and samples were harvested 3 and 6 h after incubation in the presence or absence of ABA. RACK1B was more resistant to degradation with the addition of ABA as shown by a higher abundance of RACK1B at 6 h (Fig. 5C). It is well-known that endogenous levels of ABA decrease during the first 24 h of seed imbibition^44^; therefore, we examined whether endogenous ABA regulates the sumoylation of RACK1B by using the transgenic line *35S::Flag-RACK1B/rack1b-2* (Supplementary Fig. 4). As expected, the levels of unmodified RACK1B and sumoylated RACK1B in germinating seeds gradually decreased with the time of imbibition (Fig. 5D). Furthermore, a co-immunoprecipitation assay was performed and optimized to achieve the same level of unmodified Flag-RACK1B in each well. Consistent with the unmodified RACK1B, the level of sumoylated RACK1B also decreased as endogenous ABA declined (Fig. 5E). Taken together, these results indicated that ABA enhances the stability of RACK1B by facilitating sumoylation. ABA increased the stability of RACK1B by up-regulating sumoylation and, at the same time, inhibiting ubiquitination-mediated degradation.

### RACK1B interacts with RAP2.6 and alters its affinity to *cis*-elements

**Figure 6:**
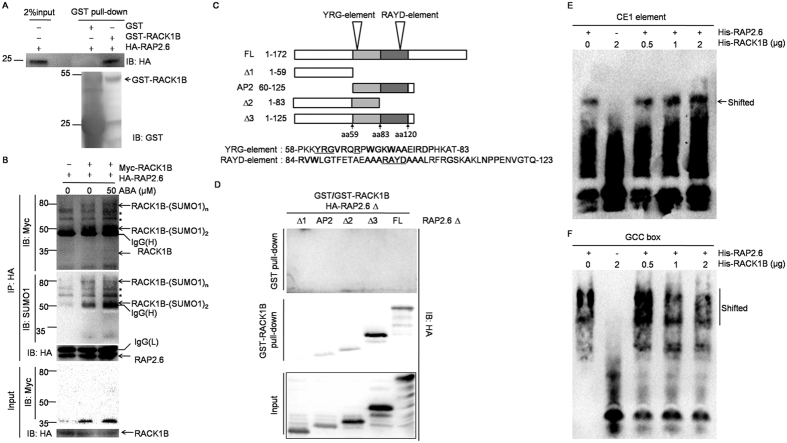
Sumoylation of RACK1B enhanced its interaction with RAP2.6. (**A**) *In vitro* GST pull-down assay. GST-RACK1B was used as bait to pull-down HA-RAP2.6 recombinant protein. The RACK1B-RAP2.6 interaction was detected by immunoblot analysis with anti-HA antibodies. (**B**) *In vivo* co-immunoprecipitation assay. *35S::Myc-RACK1B* and *35S::HA-RAP2.6* were co-expressed in Col-0 protoplasts and thereafter incubated with or without 50 μM ABA for 20 h at normal growth conditions. A co-immunoprecipitation assay was performed with anti-HA antibodies and proteins detected by immunoblotting with indicated antibodies. (**C**) Schematic diagrams of truncated versions of RAP2.6. aa, amino acid. (**D**) *In vitro* GST pull-down assays. GST-RACK1B was used to pull-down truncated RAP2.6 as indicated in (**C**). FL, full-length. (**E**,**F**) Analysis of RAP2.6 binding to CE1 element and GCC-box by EMSA. Biotin-labeled CE1 (**E**) or GCC (**F**) was used as probe. Equal amounts of His-HA-RAP2.6 protein were used in all the binding assays. Recombinant protein His-HA-RACK1B was added at the amount indicated. The band shift of probes was detected by ECL. The experiment was repeated three times with same results.

The transcription factor OsRAP2.6 interacts with OsRACK1 in the nucleus and cytoplasm^45^. *Arabidopsis* RAP2.6, which shares 94% amino acid identity with OsRAP2.6 in the AP2/ERF domain participates in ABA responses^45^. Because both RACK1 and RAP2.6 are nuclear localized^36^,^45^ and ABA enhanced the stability of RACK1B via sumoylation (Fig. 4), we assumed that AtRACK1B could interact with AtRAP2.6 and function as a scaffold protein in the ABA response. To test this hypothesis, a GST pull-down assay was performed and showed that RACK1B interacted with RAP2.6 *in vitro* (Fig. 6A). Furthermore, a co-immunoprecipitation assay showed that RACK1B and RAP2.6 could interact in *planta* (Fig. 6B). When *RACK1B* and *RAP2.6* were co-expressed in mesophyll protoplasts, RACK1B could be precipitated with anti-HA antibodies that recognize the HA tag fused to RAP2.6. RAP2.6 co-precipitated both RACK1B and sumoylated RACK1B and the intensity of co-precipitation was enhanced by exogenous ABA treatment. In addition, a greater amount of sumoylated RACK1B could be precipitated compared to unmodified RACK1B, while only unmodified RACK1B could be detected in crude extracts.

To test which domain of RAP2.6 is responsible for the interaction with RACK1B, truncated versions of RAP2.6 were generated (Fig. 6C). Based on a previous report, two conserved motifs, YRG and RAYD elements, exist in the AP2 domain of twelve RAP2 proteins^46^. As shown in Fig. 6D, the interaction could be detected when the peptide contained an intact AP2 domain (both YRG and RAYD motifs) or the YRG element alone, but not the N-terminal region of RAP2.6. Collectively, these results suggest that RACK1B interacts with RAP2.6 by direct binding to its AP2 domain and that this interaction could be enhanced by sumoylation of RACK1B.

AtRAP2.6 can bind to the GCC-box and the CE1 element, which serve as *cis*-acting regulatory elements in various phytohormone signaling pathways^47^ The intact promoter recognized by RAP2.6 remains elusive; therefore, we performed Electrophoretic mobility shift assay (EMSA) to examine whether the RACK1B-RAP2.6 interaction affects the binding of RAP2.6 to the GCC-box and/or CE1 elements. RACK1B and RAP2.6 were fused to His-tags and purified by Ni^2+^-chromatography. The result showed that the binding affinity of RAP2.6 to the CE1 element gradually improved with increasing amounts of RACK1B (Fig. 6E). In contrast, RACK1B decreased the binding affinity of RAP2.6 to the GCC-box (Fig. 6F). Taken together, these results suggest that RACK1B functions as a scaffold protein for RAP2.6 in the ABA response and alters the affinity of RAP2.6 for downstream *cis*-elements.

## Discussion

**Figure 7:**
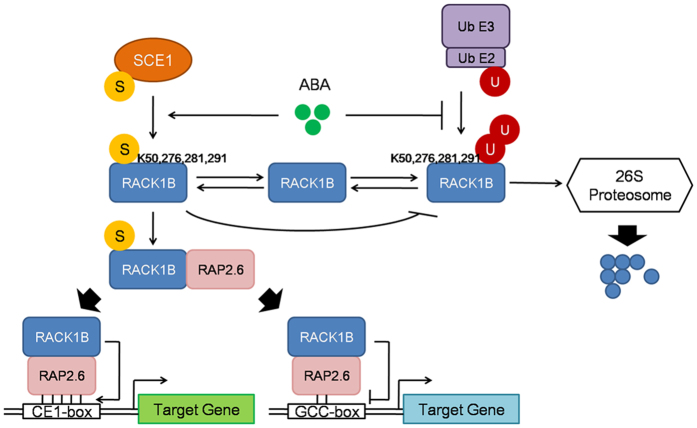
Working model for the ABA-induced increase in RACK1B stability via sumoylation. Sumoylation of RACK1B is directly mediated by SCE1A at four residues, K50, K276, K281 and K291, resulting in increased stability of RACK1B. Covalent linkage of SUMO1 (S) on RACK1B blocks some of the UBQ (U) conjugation sites in RACK1B, which prevents protein degradation. ABA facilitates the sumoylation of RACK1B and simultaneously inhibits ubiquitination, and promotes the interaction between RACK1B and RAP2.6. The consequence of this interaction results in enhanced affinity of RAP2.6 with CE1 element (five solid lines) and reduced affinity with GCC-box (two solid lines).

RACK1 proteins have been identified in all eukaryotic organisms and act as scaffold proteins that mediate protein-protein interactions or compete with other proteins for binding domains^29^. RACK1B participates in various hormone responses. For example, *rack1a-1* and *rack1a-2* mutants are insensitive to GA and BL, and produce reduced lateral roots when treated with exogenous 1-Naphthaleneacetic acid^33^. Here, we provide evidence for an expanded regulatory mechanism of ABA signaling that controls the stability of RACK1B, resulting in enhanced interaction with the transcription factor, RAP2.6. As summarized in Fig. 7, our study shows that RACK1B is sumoylated at four Lys residues via direct interaction with SCE1. The up-regulated sumoylation in response to ABA leads to increased stability and abundance of RACK1B, which results from competition with ubiquitination at the same residues. The interaction between RACK1B and RAP2.6 is also enhanced by sumoylation, which alters the affinity of RAP2.6 for its target *cis*-regulatory elements.

Studies of *Arabidopsis* sumoylation revealed that a few substrates are sumoylated in a SIZ1-dependent manner^10^,^14^,^48^,^49^,^50^. The E2-dependent RACK1B sumoylation described here is different from that in these reports (Fig. 2). This is probably due to the high affinity between RACK1B and SCE1A, which is consistent with the finding that most E3 ligase-independent sumoylation occurs at non-consensus lysine residues. Indeed, two of four identified sumoylation sites in RACK1B are non-consensus lysine residues (Fig. 2A and Supplementary Fig. 4). Results of the *in vitro* sumoylation assay suggested that SIZ1 facilitated the sumoylation of RACK1B. However, we cannot rule out the possibility that SIZ1 contributes to the site selectivity since we failed to identified the sumoylated sites *in vivo* by MS analysis.

A consequence of sumoylation can be to alter substrate stability^5^. Many substrates are both sumoylated and ubiquitinated, and often at the same lysine residues. However, the inter-relationship between these two systems is substrate specific, and can act either synergistically or antagonistically^51^,^52^. We observed that sumoylation increased RACK1B stability by antagonizing ubiquitination. The possible explanation is either two modifications share several common sites or SUMO molecule spatially blocks the ubiquitination sites or both. Besides, a form of coordination may exist between the lysine residues to balance the levels of sumoylation and ubiquitination. However, the ubiquitin complex targeting RACK1B degradation is unknown and this hypothesis requires further investigation.

It is well-known that ABA triggers multiple post-transcriptional modifications of proteins, such as phosphorylation and ubiquitination^53^,^54^. Here, we showed that ABA is also able to regulate sumoylation of target proteins. Blocking ubiquitination sites via sumoylation might be one of the regulatory mechanisms by which ABA stabilizes RACK1B (Figs 4 and 5). However, we have no direct evidence that sumoylation and ubiquitination of RACK1B are mutually exclusive in the ABA response, although ABA can inhibit the ubiquitination of RACK1B (Supplementary Fig. 6). It is possible that down-regulated ubiquitination resulted from various regulatory events, such as inhibition of the ubiquitination complex, or unidentified forms of post-transcriptional modification, instead of from direct competition for the modification of lysine residues. The varied modification status of RACK1B suggests that RACK1B is subjected to the interplay between various post-transcriptional modifications in response to different signals. A similar example is that of RACK1A phosphorylation by WNK8, which tends to be degraded in glucose signaling^55^. However, neither the phosphorylation-mimic nor the dead form of RACK1B at Ser122 and Thr161 abolished RACK1B sumoylation, although both forms affect its degradation (Supplementary Fig. 7). Therefore, the crosstalk between distinct post-transcriptional modifications of RACK1B in ABA signaling needs to be further investigated.

The interaction between RACK1B and RAP2.6 sheds light on RACK1B function that binds to one of AP2/ERF family TFs in ABA response. RAP2.6 preferentially binds to sumoylated RACK1B rather than unmodified RACK1B *in vivo*, suggesting that sumoylation facilitates RACK1B interaction with its binding partners (Fig. 6B). Additionally, ABA stimulates RACK1B sumoylation and, therefore, plays a role in regulating the dynamics of the interaction between RACK1B and RAP2.6, which could influence the expression of downstream target genes in response to ABA. Three RACK1 proteins were regarded as negative regulators of the ABA response^35^,^37^. The expression of the ABA marker gene, *RAB18*, was induced by ABA in a *rack1a* mutant and a *rack1a/rack1b* double mutant as early as 3 h after treatment^35^. However, in *RAP2.6* overexpressing plants, *RAB18* was induced after 24 h ABA treatment^47^. These downstream events continue to obscure the function of RACK1 in the ABA response. One possible explanation is that RACK1B acts as a scaffold to a wide range of proteins for distinct TFs in ABA signaling and, therefore, induction of *RAB18* in *rack1* mutants is a cumulative effect of impaired activities of distinct TFs due to loss of the platform.

RACK1 is a highly conserved WD40 repeat family protein present in a wide range of eukaryotic species, from microorganisms to plants and animals^29^. RACK1 is a scaffold protein that acts as a hub for the interaction of signaling proteins, including kinases/phosphatases^55^,^56^, G proteins^57^, transcription factors^58^, cytoskeleton proteins^29^, and the ubiquitin complex^59^. Four main effects of RACK1 on interacting proteins have been described: diversifying intermolecular interaction, changing the activity of partners, regulating the stability of partners and shuttling partners from one location to another^60^. However, in contrast to the detailed understanding of RACK1 in animals, very few studies have investigated the molecular mechanisms of RACK1 in plants. In rice, OsRACK1 is a component of the Rac1 complex and plays a role in the production of reactive oxygen species, and is involved in resistance to rice blast infection^61^. Its homolog in *Arabidopsis*, AtRACK1, is involved in a novel immune pathway that consists of the G-protein-RACK1-MAPK cascade and which transmits the signal of pathogen-secreted proteases^57^. *AtRACK1* also affects the accumulation and precision of pri-miRNAs by interacting with SERRATE and associating with the AGO1 complex^36^. Here, we postulate that a potential effect of RACK1B on RAP2.6 is to regulate its transcriptional activity because the *in vitro* EMSA result indicated that RAP2.6 has altered affinity to the GCC-box and CE1 elements once bound to RACK1B. This result also indicates that RACK1B and RAP2.6 are potential crosslink nodes between different abiotic and biotic signaling pathways. Identification of the target gene(s) of RAP2.6 will help explain the regulatory effect of RACK1B in signal transduction.

Sequence analysis revealed that RAP2 proteins are highly divergent except for the presence of at least one AP2 domain^46^. AP2/ERF proteins constitute a large family of plant specific transcription factors with more than 120 members in *Arabidopsis*^62^. Based on copies of the AP2 domain and sequence similarity, they can be categorized into three subfamilies, the AP2, ERF, and RAV subfamilies^62^,^63^. On the other hand, a number of *cis*-regulatory elements are mainly recognized by AP2/ERF transcription factors, including the drought responsive element/C-repeat responsive element (DRE/CRT)^64^, the ethylene responsive element (ERE or GCC-box)^38^, the JA and elicitor responsive element (JERE)^65^, the bipartite sequence^66^, and the ANT consensus site^67^. Because RACK1B binds directly to the AP2 domain within RAP2.6, we hypothesize that RACK1B is capable of binding different AP2/ERF members that contain an AP2 domain that shares high similarity with that of AtRAP2.6. High-throughput screening methods, such as yeast-two hybrid and co-immunoprecipitation, have identified potential binding partners^29^. Detailed analysis of these proteins will yield a better understanding of RACK1 function in plants.

## Methods

### Plant materials and growth conditions

*Arabidopsis* with a Col-0 ecotype was used in the study. The T-DNA mutants, *siz1-2* (SALK_065397)^7^, *hpy2-2* (SAIL_77_G06)^8^ and *rack1b-2* (SALK_145920)^34^ used in this study were described previously.

*Arabidopsis* seeds were surface sterilized with 70% ethanol and subsequently soaked in 10% NaClO for 10 min and then washed with sterilized H_2_O five times. Seeds were germinated on MS medium with 0.7% agar. For seed propagation, one-week-old seedlings were transferred to soil and grown in a phytotron at 22 °C with light intensity of 120 μmolm^−2^s^−1^ and a 16/8 h photoperiod.

### Generation of transgenic *Arabidopsis* plants

*RACK1B* coding sequences were amplified by PCR and cloned into pHB vector^68^ with Flag tag was fused to the N-terminus. The plasmids were transformed into *Agrobaterium tumefaciens* (EHA105) and plant transformation was performed by the floral dip method. Sequences of all the primers used in the study are listed in Supplementary Table S3.

### Antibodies

Mouse anti-HA was purchased from Youke Biotechnology (Shanghai, China), mouse anti-Myc and anti-GST, and rabbit anti-S tag antibodies were from GenScript (Nanjing, China). Rabbit anti-SUMO1 and anti-UBQ antibodies were from Agrisera (Vännäs, Sweden). Rabbit anti-TUBB2A antibody was from Sigma-Aldrich (Saint Louis, USA). The secondary antibodies, goat anti-mouse IgG-HRP (sc-2005) and goat anti-rabbit IgG-HRP (sc-2004) were from Santa Cruz (Dallas, TX, USA).

### Isolation of total RNA and first-strand synthesis of cDNA

*Arabidopsis* total RNA was isolated using TRIzol reagent (ThermoFisher, San Jose, CA, USA) according to the manufacturer’s instructions. One microgram of total RNA was used as template for first-strand cDNA synthesis using the PimeScript One Step RT-PCR Kit (Takara, Dalian, China). The cDNA was used as template for further PCR amplification of the coding sequences of indicated genes.

### Molecular cloning of *RACK1B* and site directed mutagenesis

*RACK1B* was amplified from cDNA by PCR and cloned into pMD18 with HA tag fused at N-terminus. RACK1B sequences with the following mutations, K50R, K272R, K276R, K281R, K291R, 3KR-K50, 3KR-K276, 3KR-K281, 3KR-K291 and 4KR, were synthesized (GenScript) and cloned into pUC57 with HA tag fused at N-terminus. These plasmids were used as starting plasmids for other constructions.

The His89 of SUMO1 was mutated to Arg to generate SUMO1^H89R^(GG). At the same time, Gly92 and Gly93 were mutated to Ala to generate SUMO1^H89R^(AA). SCE1A^K15R^ and SCE1A^C94S^ were generated by site directed mutagenesis. PCR was used to perform the site directed mutagenesis using specific primers and cDNA as temperate.

### Transient expression of proteins in protoplasts

Proteins were transiently expressed in *Arabidopsis* protoplasts. Four-week-old seedlings were used for mesophyll protoplast isolation and plasmid transfection according to a previously described method^69^. After incubation for 20 h in normal growth conditions, unless indicated otherwise, protoplasts were harvested for protein extraction.

### Protein purification by Ni^2+^-chromatography

Total proteins were extracted from protoplasts using lysis buffer A containing 25 mM Tris-HCl, 200 mM NaCl pH 7.8, 2 mM DTT, 1% Triton X-100 and 1 mM PMSF. Protein concentration was measured with the Bradford method and 2 mg of total proteins were used for further purification.

For purification of recombinant proteins, 50 mL *E. coli* culture were collected for protein extraction. Proteins with the hexa-histidine tag were purified using Ni^2+^-NTA sepharose (Qiagen, Venlo, Limburg, Netherlands) according to the manufacturer’s instructions. The purified protein were separated by SDS-PAGE and analyzed by immunoblotting.

### Immunoprecipitation of proteins and co-immunoprecipitation assay

Total proteins (1 mg) extracted from protoplasts were incubated with desired antibodies at 4 °C for 12 h with gentle shaking and then protein G agarose (Roche) slurry was added and incubation continued at 4 °C for another 4 h. The resin was collected and washed with 10-bed volumes of lysis buffer A three times. Proteins were then eluted with 2 × sampling buffer. For co-immunoprecipitation assays, the enriched proteins were separated by SDS-PAGE and detected by immunoblotting with another antibody. The chemiluminescence was visualized using a Tanon-5200 Chemiluminescent Imaging System (Tanon Science and Technology, Shanghai, China).

### RACK1B sumoylation assay in *Arabidopsis* mesophyll protoplasts

His-SUMO1(GG), Myc-RACK1B, Myc-RACK1B^K276R^, Myc-RACK1B^3KR-K276^ and Myc-RACK1B^4KR^ were amplified by PCR, and subsequently cloned into the expression vector, pGreenII 62-SK to generate 35S::His-SUMO1(GG), 35S::MYC-RACK1B, 35S::MYC-RACK1B^K276R^, 35S::MYC-RACK1B^3KR-K276^ and 35S::MYC-RACK1B^4KR^, respectively. His-SUMO1(GG) and different types of Myc-RACK1B proteins were transiently expressed in Arabidopsis protoplasts. The proteins sumoylated by His-SUMO1 were purified by Ni^2+^-chromatography. Sumoylation of RACK1B was detected by immunoblot analysis using anti-Myc antibodies.

### RACK1B ubiquitination assays in *Arabidopsis* mesophyll protoplasts

*His-UBQ(GG*) was amplified by PCR and cloned into pGreenII 62-SK to generate *35S::His-UBQ(GG*). *His-UBQ(GG*) was co-expressed with *Myc-RACK1B* in Col-0 protoplasts, which were then treated with 50 μM MG132 to block protein degradation or DMSO as control for 20 h. The ubiquitinated proteins were purified using Ni^2+^-chromatography and ubiquitination of RACK1B was detected by immuno blotting using anti-Myc antibodies.

### Sumoylation of RACK1B in imbibed seeds

The WT Col-0 and *Flag-RACK1B* transgenic seeds (*35S::Flag-RACK1B*/*rack1b-2*) in the background of the T-DNA insertion mutant *rack1b-2* were freshly harvested. For each individual sample, 50 mg seeds were used and imbibed in water for 0, 3, 6, 16 and 24 h. Proteins were extracted with lysis buffer A and filtrated through a 0.2 μM filter membrane (Millipore) to exclude the insoluble substances. Flag-RACK1B was immunoprecipitated with anti-DYKDDDDK beads (Clontech) and the sumoylation level of RACK1B was detected using anti-SUMO1 antibodies.

### *In vitro* sumoylation assays

For sumoylation assay in *E. coli* system, pACYCDuet-1 and pCDFDuet-1 were used to reconstitute the *Arabidopsis* SUMO pathway in *E. coli* as described^41^. pACYCDuet-1 carries two subunits of E1 (*SAE1B* and *SAE2*). *SAE1B* and *SAE2* were fused with an S-tag and His tag, respectively. pCDFDuet-1 carries a SUMO molecule [*SUMO1*^*H89R*^(*GG*), *SUMO1*^*H89R*^(*AA*), *SUMO3(GG*) or *SUMO5(GG*)] and E2 (*SCE1A, SCE1A*^*K15R*^or *SCE1A*^*C94S*^). SUMO and SCE1A were fused with a His tag and S-tag, respectively. HA-RACK1B was cloned into pET28a such that the His tag was fused at the N-terminus. All three plasmids were co-transformed into *E. coli* BL21 (DE3) competent cells. The transformants were inoculated and cultured at 37 °C to an OD_600_ of approximately 0.6. IPTG was added to a final concentration of 0.2 mM. After induction for 12 h at 25 °C, the cells were harvested and re-suspended in PBS buffer. Cells were lysed by sonication, centrifuged; and the supernatant was collected. The proteins were detected by immunoblot analysis after separation by SDS-PAGE.

For *in vitro* sumoylation assay, the coding sequences of *SUMO1, SAE1B, SAE2, SCE1, SIZ1* and *HPY2* were cloned into pET28a. The expression, purification and *in vitro* sumoylation assay were performed as described^7^,^70^.

### Identification of sumoylation sites using mass spectrometry

The crude extracts from *in vitro* sumoylation assays were purified by Ni^2+^-chromatography and separated by 10% SDS-PAGE. The gel was Coomassie Brilliant Blue-stained and the bands corresponding to sumoylated RACK1B were cut out (Supplemental Fig. 2). The gel slices were cleaned, desalted and concentrated via vacuum centrifugation and then digested with 5ng/μL trypsin at 37 °C for at least 20 h. Tryptic peptides were acidified with 100 μL extraction buffer (60% ACN, 0.1% TFA).

LC-MS/MS analyses were performed using QE mass spectrometry with a nano-electrospray ion source (ThermoFisher) coupled to a ThermoFisher Easy nLC1000 high performance liquid chromatography system equipped with a 0.15 mm × 150 mm Zorbax 300SB-C18 column. Peptides were separated using a linear gradient of 4–50% acetonitrile (0.1% formic acid) for 50 min, 50–100% acetonitrile (0.1% formic acid) from 50 min to 54 min, and 100% acetonitrile for 60 min. MS spectra were acquired in full-scan mode at a resolution of 100,000 and 10 MS/MS spectra were acquired per MS. MS/MS precursor selection met the criteria that the charge state be known and ≥ 2. Peptide sequences were searched using MASCOT 2.2 software against the *Arabidopsis* protein database and common contaminants. Search parameters included a precursor mass tolerance of 2.5 Da, fragment ion mass tolerance of 0.2 Da, up to two missed cleavages, oxidation of Met, variable modification of Lys residues by sumoylation (QTGG, +343.1492 *m/z* or pyroQTGG, +326.1226 *m/z*), and a filter by score ≥45.

### *In vitro* GST pull-down assay

To verify the interaction of RACK1B with SCE1A and SIZ1, coding sequences of *RACK1B, SCE1A* and *SIZ1* were cloned into pGS-21a (GenScript) to generate GST-RACK1B, GST-SCE1 and GST-SIZ1, respectively.

To verify the interaction between RACK1B and RAP2.6, HA tagged RAP2.6 was cloned into pET28a and pGreenII 62-SK to generate His-HA-RAP2.6 and *35S::HA-RAP2.6*, respectively. To examine the domain of RAP2.6 that interacted with RACK1B, HA-tagged truncated peptides of RAP2.6 were generated by PCR and cloned into pET28a to generate expression vectors.

GST-RACK1B, GST-SIZ1, His-HA-RACK1B or SCE1A-S was transformed into *E. coli* BL21 (DE3). His-HA-RAP2.6 and truncated peptides were expressed in *E. coli* Rosetta (DE3). Transformed strains were first grown at 37 °C until OD_600_ reached 0.6~0.8. Cultures were then induced with 0.1 mM IPTG for 2 h at 25 °C. Cells were harvested in binding buffer (50 mM Tris-HCl pH 7.8, 200 mM NaCl, 1% Triton X-100, and 10% glycerol) at a ratio of 1:10. For the SIZ1-RACK1B interaction, 500 μL crude extract of GST-SIZ1 for each individual trial was incubated with 30 μL glutathione-Sepharose beads (GenScript) for 1 h on ice. After washing three times with 20 volumes of binding buffer, the beads were incubated with 200 μL crude extract of His-HA-RACK1B for another 1 h on ice. After another wash step, the bound proteins were eluted with 2 × SDS-PAGE sampling buffer. The same binding conditions were used for RACK1B-SCE1A and RACK1B-RAP2.6 (or truncated RAP2.6) interactions, except that 100 μL crude extract of GST-RACK1B was used as bait protein and equal volumes of crude extracts of SCE1A-S and His-HA-RAP2.6 (or truncated mutants) were used as prey proteins. The interactions were detected by immunoblot analysis using the antibodies indicated.

### EMSA

Oligonucleotides 4 × CE1 and 4 × GCC element was synthesized and biotin labeled (Genewiz, Suzhou, China) with the sequences: 5′-GGAATTC TGCCACCGG TGCCACCGG TGCCACCGG TGCCACCGG TCTAGAGC-3′ and 5′-GGAATTC AGCCGCC AGCCGCC AGCCGCC AGCCGCC TCTAGAGC-3′, respectively^47^. The protein His-HA-RAP2.6 was expressed and induced as described above, and purified by Ni-NTA chromatography (Qiagen). In the binding assays, 1 μg His-RAP2.6 and 50 fmol biotin-labeled CE1 element, or 0.5 μg His-HA-RAP2.6 and 100 fmol biotin-labeled GCC-box was used. Purified His-RACK1B was added as indicated. The binding reaction (in a volume of 20 μl) was performed in binding buffer containing 10 mM Tris, 50 mM KCl, 1 mM DTT, 1 μg poly (dI·dC), pH 7.5. Binding reactions were incubated at room temperature for 20 min and analyzed by PAGE on 6% gels in 0.5 × TBE buffer. Gels were blotted on Hybond-N^+^ membranes (GE healthcare, Uppsala, Sweden) and signals were detected using a Light Shift Chemiluminescent EMSA Kit (ThermoFisher) according to the manufacturer’s instructions.

### Data availability

The authors declare that the data supporting the findings of the study are available within the article and its Supplementary Information files or are available from the corresponding author upon request.

## Additional Information

**How to cite this article**: Guo, R. and Sun, W. Sumoylation stabilizes RACK1B and enhance its interaction with RAP2.6 in the abscisic acid response. *Sci. Rep.* **7**, 44090; doi: 10.1038/srep44090 (2017).

**Publisher's note:** Springer Nature remains neutral with regard to jurisdictional claims in published maps and institutional affiliations.

## Supplementary Material

Supplementary Figures

Supplementary Table S1

Supplementary Table S2

Supplementary Table S3
