# Supplementary material for: Sumoylation stabilizes RACK1B and enhance its interaction with RAP2.6 in the abscisic acid response
Source: Sci Rep. 2017 Mar 8;7:44090. doi: 10.1038/srep44090 (PMC5341030; doi:10.1038/srep44090)
Supplement: Supplementary Figures [file srep44090-s1.pdf]

Sumoylation stabilizes RACK1B and enhance its interaction with RAP2.6 in the abscisic acid response.

Rongkai Guo[1,2] and Weining Sun[1,*]

Supplementary Information

Supplementary Figure 1

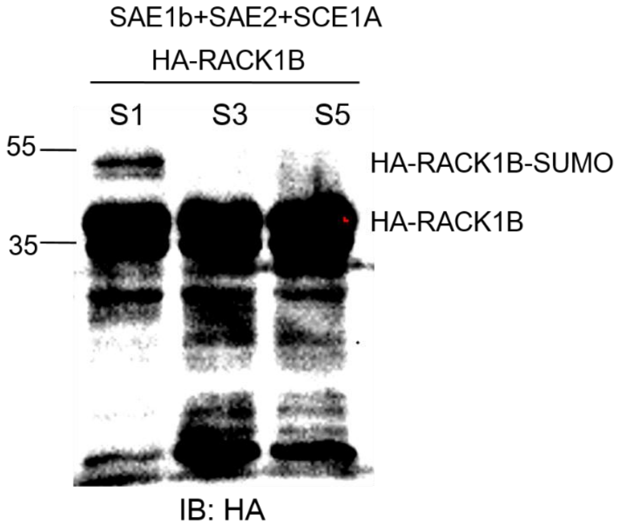

Supplementary Figure 1. Paralog specific sumoylation of RACK1B. Sumoylation of RACK1B by SUMO1, SUMO3 and SUMO5 was examined in *in vitro* sumoylation system where His-SUMO1(GG) was replaced by His-SUMO3(GG) or His-SUMO5(GG), respectively. The sumoylation of RACK1B was detected by immunoblotting with anti-HA antibodies.

Supplementary Figure 2

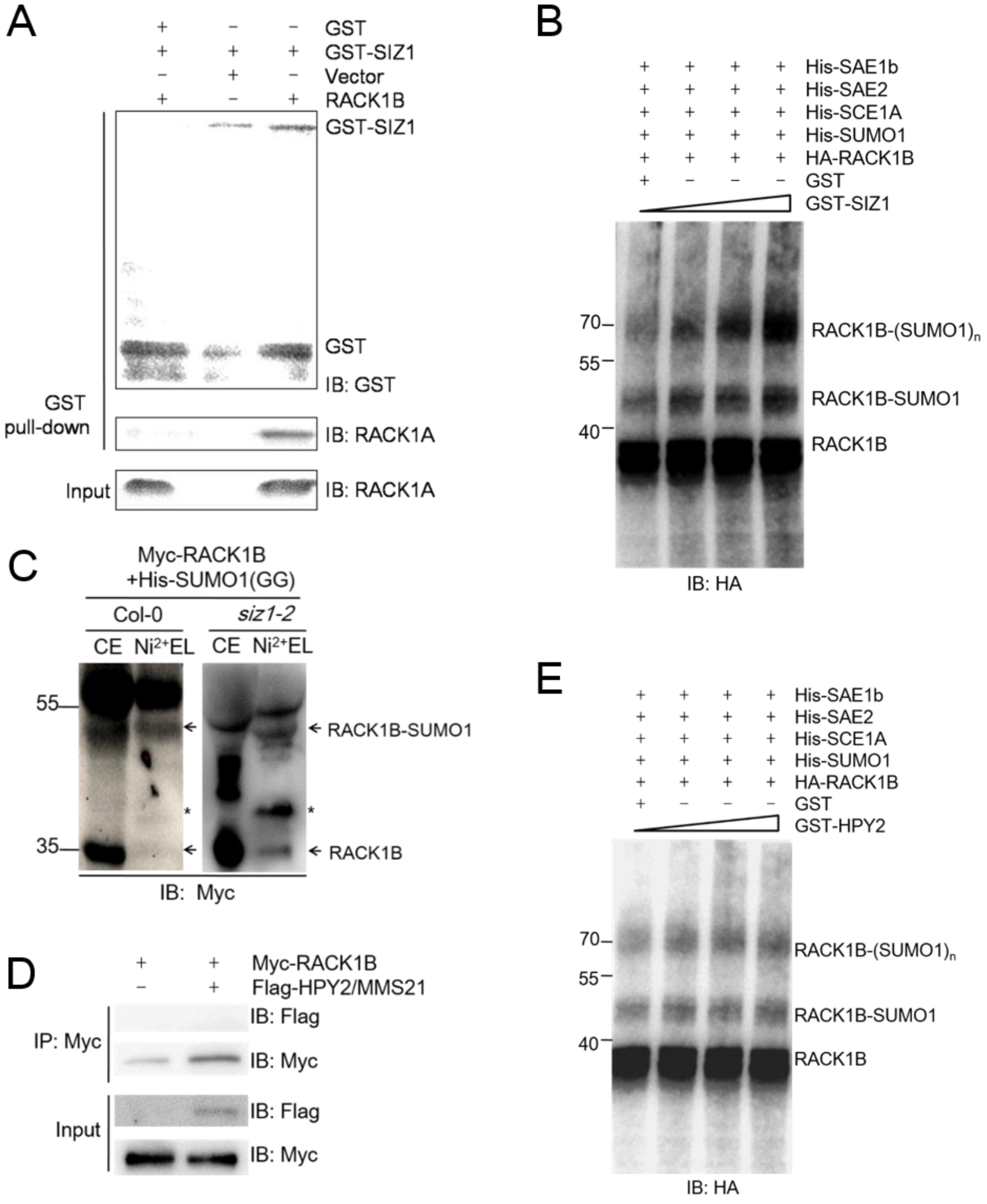

Supplementary Figure 2. Analysis of function of E3 ligase on RACK1B sumoylation. A) GST pull-down assay. GST-SIZ1 was used as bait to pull-down HA-RACK1B. GST-SIZ1 was purified with glutathione-sepharose slurry and thereafter incubated with the crude extract containing HA-RACK1B. Target proteins were eluted with 2 × sampling buffer and detected by immunoblot and with antibodies indicated. B) *In vitro* sumoylation assay. His-HA-RACK1B, His-SUMO1(GG), His-SAE1B, His-SAE2, His-SCE1A and GST-SIZ1 were individually purified, and

mixed with 0, 50, 100 and 200 ng of SIZ1 and incubated at 37°C for 3 h. The sumoylation level of RACK1B was detected with anti-HA antibodies. C) Sumoylation of RACK1B in a *siz1-2* background. The constructs, *35S::Myc-RACK1B* and *35S::His-SUMO1(GG)*, were co-transformed into Col-0 and *siz1-2* protoplasts. The existence of RACK1B sumoylation in the two backgrounds was detected in the crude extract (CE) or in elutes from Ni^{2+}-chromatography (Ni^{2+} EL) by immunoblot analysis with anti-Myc antibodies. * indicates a nonspecific immunoreactive band. D) *In vivo* co-immunoprecipitation assay. *35S::Myc-RACK1B* was co-expressed in Col-0 protoplasts with or without *35S::Flag-HPY2/MMS21*. One milligram of individual crude extract was subjected to a co-immunoprecipitation assay with anti-Myc antibodies. E) *In vitro* sumoylation assay. His-HA-RACK1B, His-SUMO1(GG), His-SAE1B, His-SAE2, His-SCE1A and GST-HPY2 were individually purified, and mixed with 0, 50, 100 and 200 ng of HPY2 and incubated at 37°C for 3 h. The sumoylation level of RACK1B was detected with anti-HA antibodies.

Supplementary Figure 3

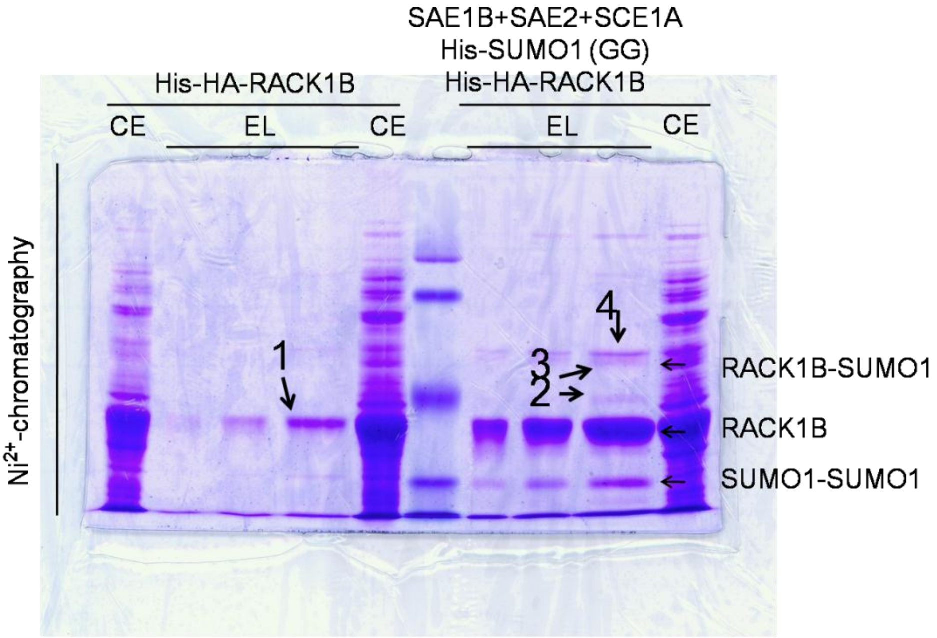

Supplementary Figure 3. Purification of sumoylated RACK1B in *in vitro* sumoylation system subjected to MS scan. His-HA-RACK1B was expressed in *E. coli* BL21 (DE3) (lane 1-4) and *E. coli* sumoylation system (lane 7-10), respectively. Hexa-histidin fused proteins were purified via Ni^{2+}-chromatography and separated on 10% SDS-PAGE. The arrow indicated bands that were cut from the gel and subjected to LC-MS/MS identification. The free RACK1B and sumoylated RACK1B were discovered at band #1 and #3, respectively. CE, crude extract; EL, elute from Ni^{2+}-NTA.

Supplementary Figure 4

A

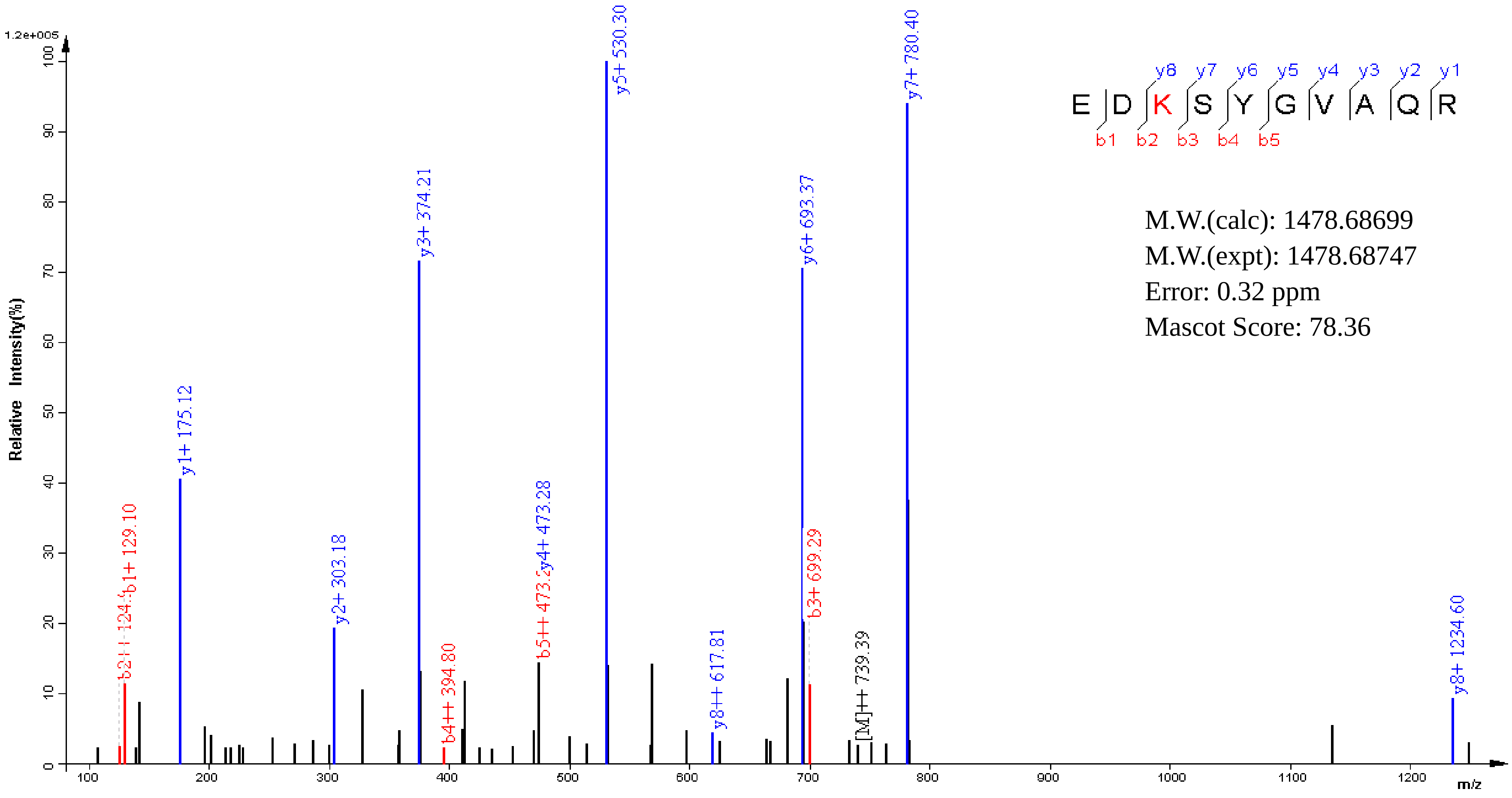

B

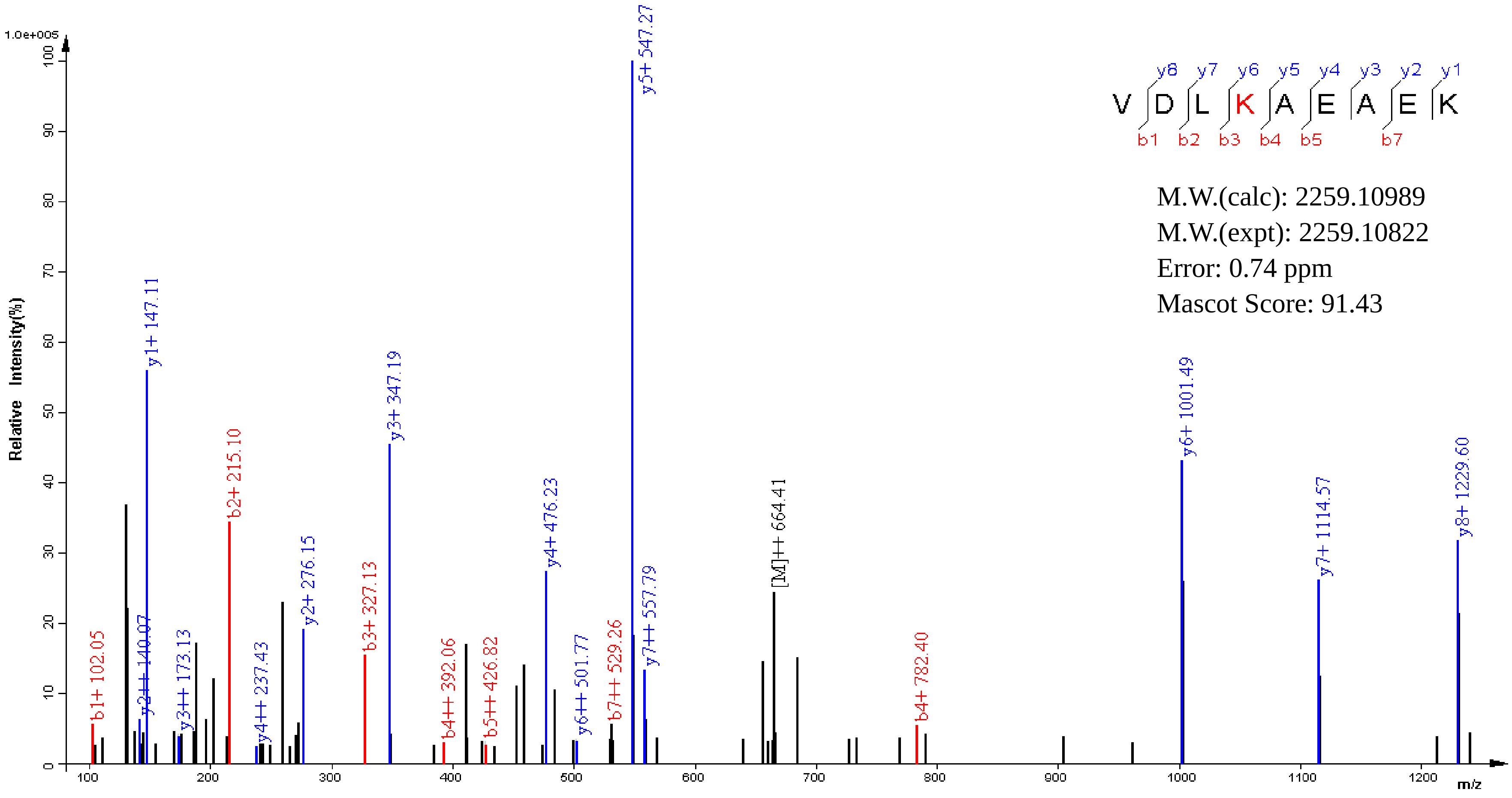
1.0e+005
Relative Intensity(%)
m/z
V D L K A E A E K
y8 y7 y6 y5 y4 y3 y2 y1
b1 b2 b3 b4 b5 b7
M.W.(calc): 2259.10989
M.W.(expt): 2259.10822
Error: 0.74 ppm
Mascot Score: 91.43
b1+ 102.05
y2++ 140.07
y1+ 147.11
y3++ 173.13
b2+ 215.10
y4++ 237.43
y2+ 276.15
b3+ 327.13
y3+ 347.19
b4++ 392.06
b5++ 426.82
y4+ 476.23
y6++ 501.77
b7++ 529.26
y5+ 547.27
y7++ 557.79
[M]++ 664.41
b4+ 782.40
y6+ 1001.49
y7+ 1114.57
y8+ 1229.60

C

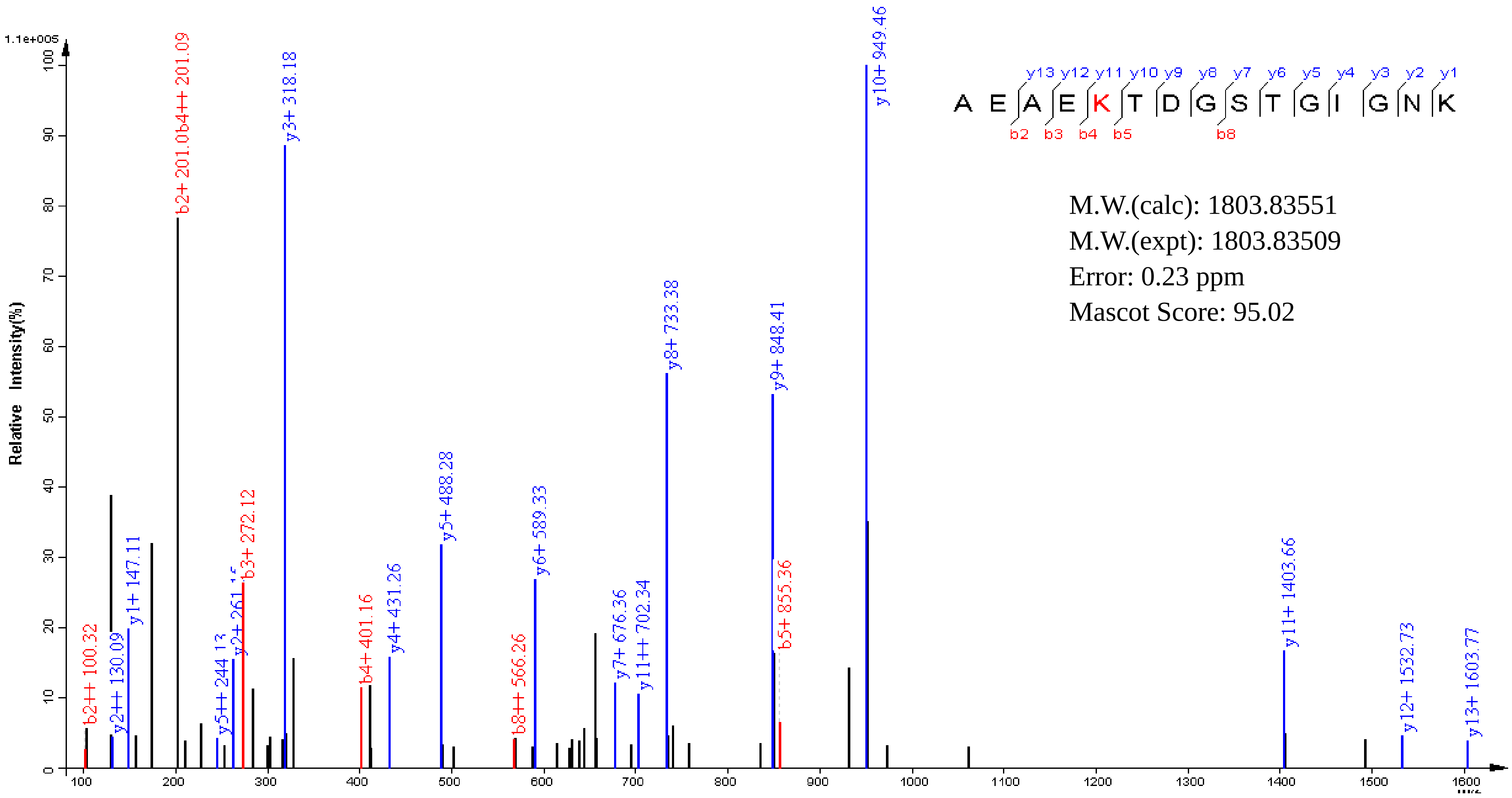

A E A E K T D G S T G I G N K
y13 y12 y11 y10 y9 y8 y7 y6 y5 y4 y3 y2 y1
b2 b3 b4 b5 b8
M.W.(calc): 1803.83551
M.W.(expt): 1803.83509
Error: 0.23 ppm
Mascot Score: 95.02
Relative Intensity(%)
1.1e+005
b2++ 100.32
y2++ 130.09
y1+ 147.11
b2+ 201.0b4++ 201.09
y5++ 244.13
y2+ 261.15
b3+ 272.12
y3+ 318.18
b4+ 401.16
y4+ 431.26
y5+ 488.28
b8++ 566.26
y6+ 589.33
y7+ 676.36
y11++ 702.34
y8+ 733.38
y9+ 848.41
b5+ 855.36
y10+ 949.46
y11+ 1403.66
y12+ 1532.73
y13+ 1603.77

D

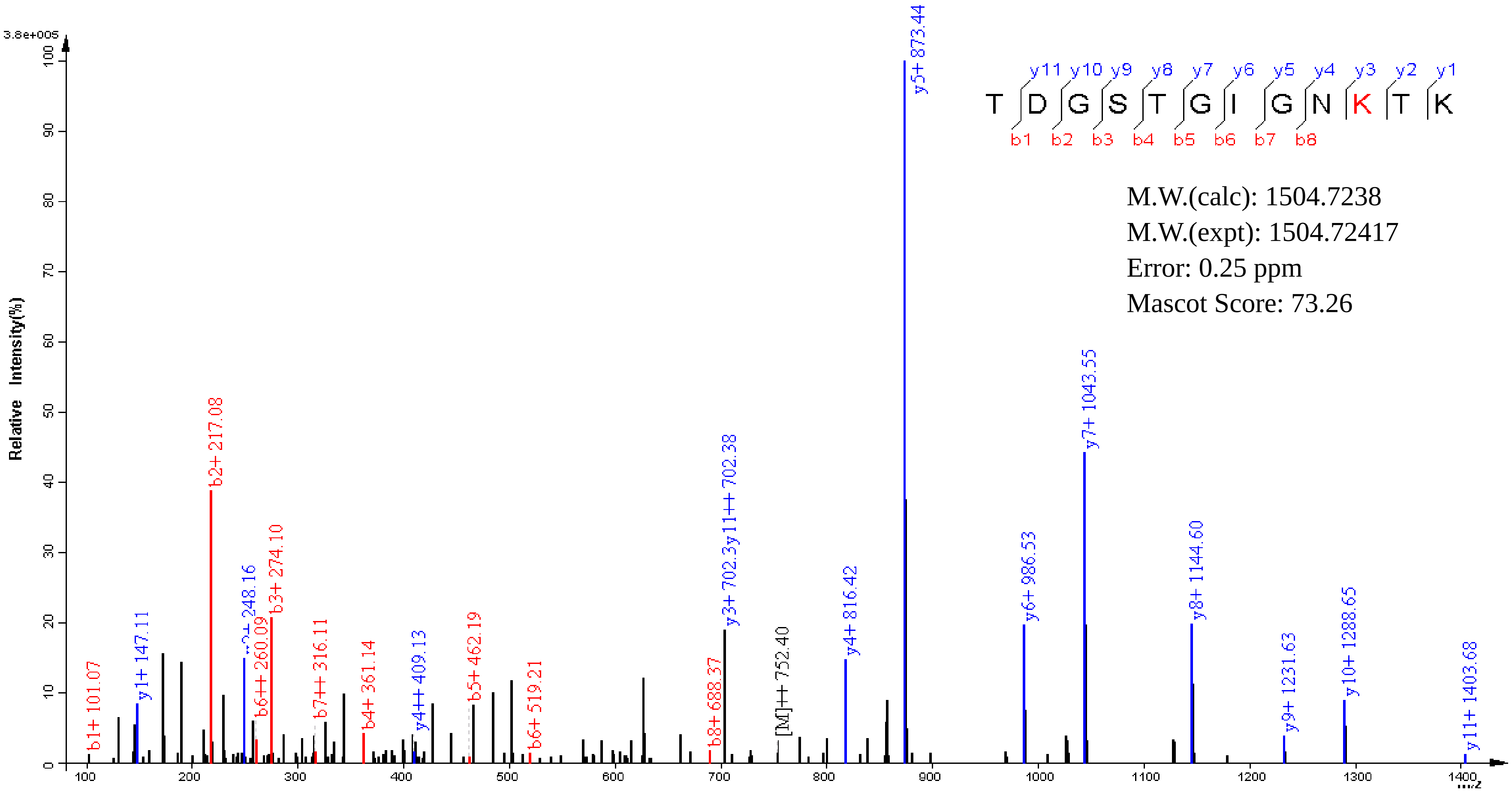

T D G S T G I G N K T K
y11 y10 y9 y8 y7 y6 y5 y4 y3 y2 y1
b1 b2 b3 b4 b5 b6 b7 b8
M.W.(calc): 1504.7238
M.W.(expt): 1504.72417
Error: 0.25 ppm
Mascot Score: 73.26
Relative Intensity(%)
3.8e+005
y5+ 873.44
y7+ 1043.55
b2+ 217.08
b3+ 274.10
y3+ 702.3y11++ 702.38
y6+ 986.53
y8+ 1144.60
y4+ 816.42
b6++ 260.09
y1+ 147.11
b7++ 316.11
b4+ 361.14
y4++ 409.13
b5+ 462.19
b6+ 519.21
b1+ 101.07
b8+ 688.37
[M]++ 752.40
y9+ 1231.63
y10+ 1288.65
y11+ 1403.68
m/z

Supplementary Figure 4. MS identification of SUMO1 residues on RACK1B. A) A representative RACK1B peptide (residues 48-57) containing an isopeptide-linked QTGG sequence attached to K50 which upon MS/MS analysis generated a SUMO footprint of pyroQTGG (K+ 326.1226 m/z). B) A representative RACK1B peptide (residues 273-281) containing an isopeptide-linked QTGG sequence attached to K276 which upon MS/MS analysis generated a SUMO footprint of QTGG (K+ 343.1492 m/z). C) A representative RACK1B peptide (residues 277-291) containing an isopeptide-linked QTGG sequence attached to K281 which upon MS/MS analysis generated a SUMO footprint of pyroQTGG (K+ 326.1226 m/z). D) A representative RACK1B peptide (residues 282-293) containing an isopeptide-linked QTGG sequence attached to K291 which upon MS/MS analysis generated a SUMO footprint of pyroQTGG (K+ 326.1226 m/z).

Supplementary Figure 5

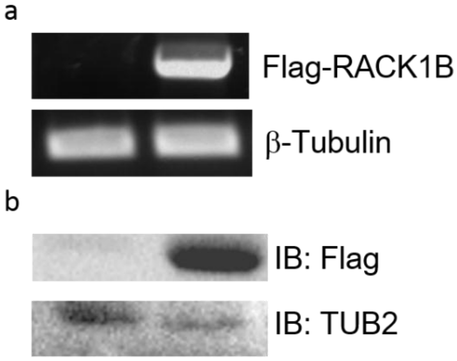

Supplementary Figure 5. Identification of RACK1B expression in *35S::Flag-RACK1B/rack1b-2* transgenic plants. The *rack1b-2* mutant was transformed with *35S::Flag-RACK1B* by using floraldip method. A) RT-PCRanalysis of *Flag-RACK1B* expression with primers (F: 5'-gattacaaaggatgacgacgataag-3' and R: 5'-ctagtaacgaccaataccccagaccc-3'). β-Tublin was used as internal control. B) Western blot analysis of Flag-RACK1B in transgenic plants with anti-Flag antibodies or anti-TUB2 antibodies as loading control.

Supplementary Figure 6

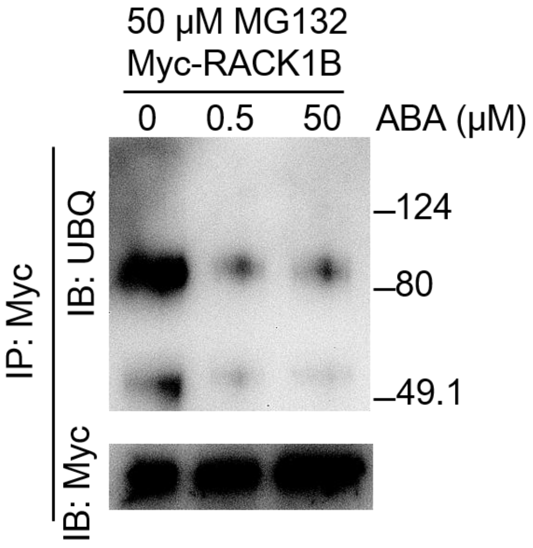

Supplementary Figure 6. ABA inhibited the ubiquitination of RACK1B. *In vivo* ubiquitinylation assay. *35S::Myc-RACK1B* was expressed in Col-0 protoplasts and treated with 0, 0.5 or 50 μM ABA for 2 h, after which 50 μM MG132 were added and incubated for another 18 h. The individual proteins were immuno precipitated using anti-Myc antibodies and detected by immunoblot with anti-UBQ antibodies.

Supplementary Figure 7

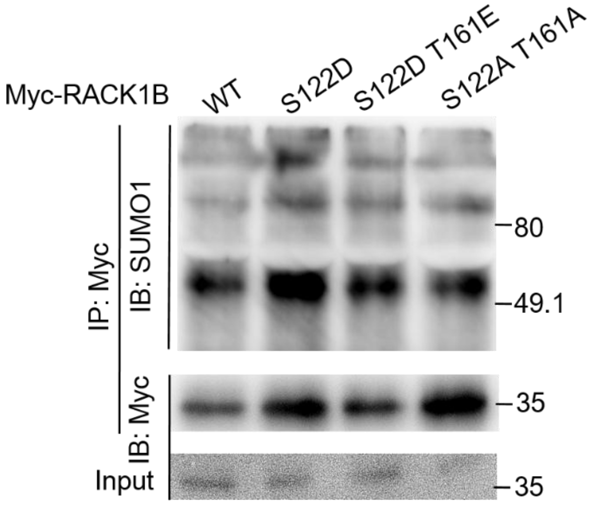

Supplementary Figure 7. Phosphorylation of RACK1B at S122 and T161 has no effect on its sumoylation. *35S::His-SUMO1(GG)* was co-transformed with *35S::Myc-RACK1B*, *35S::Myc-RACK1B*^{S122D}, *35S::Myc-RACK1B*$^{S122D/T161E}$, or *35S::Myc-RACK1B*$^{S122A/T161A}$ in Col-0 protoplasts and thereafter cultivated at normal growth conditions for 20 h. Individual proteins were subjected to immuno precipitation assay with anti-Myc antibodies. The sumoylation of RACK1B and RACK1B mutants were detected by immunoblot with antibodies indicated
