# Supplementary material for: Sumoylation stabilizes RACK1B and enhance its interaction with RAP2.6 in the abscisic acid response
Source: Sci Rep. 2017 Mar 8;7:44090. doi: 10.1038/srep44090 (PMC5341030; doi:10.1038/srep44090)
Supplement: Supplementary Table S1 [file srep44090-s2.pdf]

Supplementary Table S1. MS scan for sumoylation sites of RACK1B.

	Reference	PepCount	UniquePepCount	CoverPercen	MW	PI
	FileScan	Sequence	MH+	Diff(MH+)	Charge	Rank
$1-1	R14009_38	733	30	68.40%	35800.11	6.66
	R14009_38_3,5607	K.AEAEK#TDGSTGIGNK.T	1803.83551	-0.00168	2	1
	R14009_38_3,5647	K.AEAEK#TDGSTGIGNK.T	1803.83551	-0.00138	2	1
	R14009_38_3,5679	K.AEAEK#TDGSTGIGNK.T	1803.83551	-0.00082	2	1
	R14009_38_3,5712	K.AEAEK#TDGSTGIGNK.T	1803.83551	-0.00042	2	1
	R14009_38_3,5748	K.AEAEK#TDGSTGIGNK.T	1803.83551	-0.00142	2	1
	R14009_38_3,5783	K.AEAEK#TDGSTGIGNK.T	1803.83551	-0.00064	2	1
	R14009_38_3,3615	K.AEAEK@TDGSTGIGNK.T	1820.86206	0.00121	2	1
	R14009_38_3,3621	K.AEAEK@TDGSTGIGNK.T	1820.86206	-0.00046	3	1
	R14009_38_3,3650	K.AEAEK@TDGSTGIGNK.T	1820.86206	0.00205	2	1
	R14009_38_3,3661	K.AEAEK@TDGSTGIGNK.T	1820.86206	0.00014	3	1
	R14009_38_3,3709	K.AEAEK@TDGSTGIGNK.T	1820.86206	0.00127	2	1
	R14009_38_3,3763	K.AEAEK@TDGSTGIGNK.T	1820.86206	-0.00199	2	1
	R14009_38_3,3773	K.AEAEK@TDGSTGIGNK.T	1820.86206	0.0008	3	1
	R14009_38_3,3801	K.AEAEK@TDGSTGIGNK.T	1820.86206	0.00021	2	1
	R14009_38_3,3813	K.AEAEK@TDGSTGIGNK.T	1820.86206	0.00098	3	1
	R14009_38_3,3834	K.AEAEK@TDGSTGIGNK.T	1820.86206	0.00087	2	1
	R14009_38_3,3847	K.AEAEK@TDGSTGIGNK.T	1820.86206	0.00062	3	1
	R14009_38_3,3866	K.AEAEK@TDGSTGIGNK.T	1820.86206	0.00047	2	1
	R14009_38_3,3887	K.AEAEK@TDGSTGIGNK.T	1820.86206	0.00074	3	1
	R14009_38_3,3899	K.AEAEK@TDGSTGIGNK.T	1820.86206	0.00045	2	1
	R14009_38_3,2935	K.AEAEKTDGSTGIGNK.T	1477.71287	0.00081	2	1
	R14009_38_3,2975	K.AEAEKTDGSTGIGNK.T	1477.71287	0.00109	2	1
	R14009_38_3,3015	K.AEAEKTDGSTGIGNK.T	1477.71287	0.00081	2	1
	R14009_38_3,3050	K.AEAEKTDGSTGIGNK.T	1477.71287	0.00109	2	1
	R14009_38_3,3087	K.AEAEKTDGSTGIGNK.T	1477.71287	0.00107	2	1
	R14009_38_3,25140	K.DGVILLWDLAEGKK.L	1556.86825	-0.00099	2	1
	R14009_38_3,25173	K.DGVILLWDLAEGKK.L	1556.86825	-0.00152	3	1
	R14009_38_3,25181	K.DGVILLWDLAEGKK.L	1556.86825	-0.00081	2	1
	R14009_38_3,25213	K.DGVILLWDLAEGKK.L	1556.86825	-0.00125	3	1
	R14009_38_3,25220	K.DGVILLWDLAEGKK.L	1556.86825	-0.00029	2	1
	R14009_38_3,25253	K.DGVILLWDLAEGKK.L	1556.86825	-0.00047	2	1
	R14009_38_3,25254	K.DGVILLWDLAEGKK.L	1556.86825	-0.0014	3	1
	R14009_38_3,25286	K.DGVILLWDLAEGKK.L	1556.86825	-0.00069	2	1
	R14009_38_3,25287	K.DGVILLWDLAEGKK.L	1556.86825	-0.00291	3	1
	R14009_38_3,25319	K.DGVILLWDLAEGKK.L	1556.86825	-0.00067	2	1
	R14009_38_3,25320	K.DGVILLWDLAEGKK.L	1556.86825	-0.00327	3	1
	R14009_38_3,25352	K.DGVILLWDLAEGKK.L	1556.86825	-0.00071	2	1
	R14009_38_3,25353	K.DGVILLWDLAEGKK.L	1556.86825	-0.00338	3	1
	R14009_38_3,25385	K.DGVILLWDLAEGKK.L	1556.86825	-0.00123	2	1
	R14009_38_3,25386	K.DGVILLWDLAEGKK.L	1556.86825	-0.00476	3	1
	R14009_38_3,25418	K.DGVILLWDLAEGKK.L	1556.86825	-0.00073	2	1
	R14009_38_3,25419	K.DGVILLWDLAEGKK.L	1556.86825	-0.00372	3	1
	R14009_38_3,25451	K.DGVILLWDLAEGKK.L	1556.86825	-0.00073	2	1
	R14009_38_3,25452	K.DGVILLWDLAEGKK.L	1556.86825	-0.00354	3	1
	R14009_38_3,25484	K.DGVILLWDLAEGKK.L	1556.86825	-0.00049	2	1
	R14009_38_3,25485	K.DGVILLWDLAEGKK.L	1556.86825	-0.00374	3	1
	R14009_38_3,25517	K.DGVILLWDLAEGKK.L	1556.86825	-0.00041	2	1
	R14009_38_3,25518	K.DGVILLWDLAEGKK.L	1556.86825	-0.0026	3	1
	R14009_38_3,25550	K.DGVILLWDLAEGKK.L	1556.86825	-0.00073	2	1
	R14009_38_3,25551	K.DGVILLWDLAEGKK.L	1556.86825	-0.00221	3	1
	R14009_38_3,25583	K.DGVILLWDLAEGKK.L	1556.86825	-0.00075	2	1
	R14009_38_3,25584	K.DGVILLWDLAEGKK.L	1556.86825	-0.00174	3	1
	R14009_38_3,25616	K.DGVILLWDLAEGKK.L	1556.86825	-0.00053	2	1
	R14009_38_3,25617	K.DGVILLWDLAEGKK.L	1556.86825	-0.00161	3	1
	R14009_38_3,25649	K.DGVILLWDLAEGKK.L	1556.86825	-0.00041	2	1
	R14009_38_3,25651	K.DGVILLWDLAEGKK.L	1556.86825	-0.00098	3	1
	R14009_38_3,25684	K.DGVILLWDLAEGKK.L	1556.86825	-0.00023	2	1
	R14009_38_3,25685	K.DGVILLWDLAEGKK.L	1556.86825	-0.00194	3	1
	R14009_38_3,25717	K.DGVILLWDLAEGKK.L	1556.86825	-0.00017	2	1
	R14009_38_3,25718	K.DGVILLWDLAEGKK.L	1556.86825	-0.00161	3	1
	R14009_38_3,25750	K.DGVILLWDLAEGKK.L	1556.86825	-0.00013	2	1
	R14009_38_3,25751	K.DGVILLWDLAEGKK.L	1556.86825	-0.00084	3	1
	R14009_38_3,25783	K.DGVILLWDLAEGKK.L	1556.86825	-0.00015	2	1
	R14009_38_3,25785	K.DGVILLWDLAEGKK.L	1556.86825	-0.00077	3	1
	R14009_38_3,25816	K.DGVILLWDLAEGKK.L	1556.86825	-0.00027	2	1
	R14009_38_3,25828	K.DGVILLWDLAEGKK.L	1556.86825	-0.00093	3	1
	R14009_38_3,25850	K.DGVILLWDLAEGKK.L	1556.86825	-0.00031	2	1

FileScan	Score	DeltaScor	ExpectValue	Ions	Referenc	DIFF MODIFIED CANDIDA	PI	MissCleavag	Modification	MatchedTIC	NumProteaseTermir	Engine	Decoy
R14009_38_3,5607	56.51	0	1.12E-07	0|0	R14009_38		4.68	1	326.122635 pQTGG (K)	0	2	MASCOT	T
R14009_38_3,5647	68.4	0	7.23E-09	0|0	R14009_38		4.68	1	326.122635 pQTGG (K)	0	2	MASCOT	T
R14009_38_3,5679	79.28	0	5.90E-10	0|0	R14009_38		4.68	1	326.122635 pQTGG (K)	0	2	MASCOT	T
R14009_38_3,5712	95.02	0	1.57E-11	0|0	R14009_38		4.68	1	326.122635 pQTGG (K)	0	2	MASCOT	T
R14009_38_3,5748	62.75	0	2.65E-08	0|0	R14009_38		4.68	1	326.122635 pQTGG (K)	0	2	MASCOT	T
R14009_38_3,5783	48.07	0	7.80E-07	0|0	R14009_38		4.68	1	326.122635 pQTGG (K)	0	2	MASCOT	T
R14009_38_3,3615	21.81	0	3.30E-04	0|0	R14009_38		4.68	1	343.149185 QTGG (K)	0	2	MASCOT	T
R14009_38_3,3621	31	0	3.97E-05	0|0	R14009_38		4.68	1	343.149185 QTGG (K)	0	2	MASCOT	T
R14009_38_3,3650	27.17	0	9.59E-05	0|0	R14009_38		4.68	1	343.149185 QTGG (K)	0	2	MASCOT	T
R14009_38_3,3661	45.58	0	1.38E-06	0|0	R14009_38		4.68	1	343.149185 QTGG (K)	0	2	MASCOT	T
R14009_38_3,3709	21.29	0	3.72E-04	0|0	R14009_38		4.68	1	343.149185 QTGG (K)	0	2	MASCOT	T
R14009_38_3,3763	55.41	0	1.44E-07	0|0	R14009_38		4.68	1	343.149185 QTGG (K)	0	2	MASCOT	T
R14009_38_3,3773	41.23	0	3.77E-06	0|0	R14009_38		4.68	1	343.149185 QTGG (K)	0	2	MASCOT	T
R14009_38_3,3801	72.24	0	2.99E-09	0|0	R14009_38		4.68	1	343.149185 QTGG (K)	0	2	MASCOT	T
R14009_38_3,3813	47.81	0	8.28E-07	0|0	R14009_38		4.68	1	343.149185 QTGG (K)	0	2	MASCOT	T
R14009_38_3,3834	59.66	0	5.41E-08	0|0	R14009_38		4.68	1	343.149185 QTGG (K)	0	2	MASCOT	T
R14009_38_3,3847	45.6	0	1.38E-06	0|0	R14009_38		4.68	1	343.149185 QTGG (K)	0	2	MASCOT	T
R14009_38_3,3866	53.4	0	2.29E-07	0|0	R14009_38		4.68	1	343.149185 QTGG (K)	0	2	MASCOT	T
R14009_38_3,3887	59.95	0	5.06E-08	0|0	R14009_38		4.68	1	343.149185 QTGG (K)	0	2	MASCOT	T
R14009_38_3,3899	54.32	0	1.85E-07	0|0	R14009_38		4.68	1	343.149185 QTGG (K)	0	2	MASCOT	T
R14009_38_3,2935	25.14	0	1.53E-04	0|0	R14009_38		4.68	1		0	2	MASCOT	T
R14009_38_3,2975	96.74	0	1.06E-11	0|0	R14009_38		4.68	1		0	2	MASCOT	T
R14009_38_3,3015	106.55	0	1.11E-12	0|0	R14009_38		4.68	1		0	2	MASCOT	T
R14009_38_3,3050	99.34	0	5.82E-12	0|0	R14009_38		4.68	1		0	2	MASCOT	T
R14009_38_3,3087	62.23	0	2.99E-08	0|0	R14009_38		4.68	1		0	2	MASCOT	T
R14009_38_3,25140	32.51	0	2.81E-05	0|0	R14009_38		4.56	1		0	2	MASCOT	T
R14009_38_3,25173	26.87	0	1.03E-04	0|0	R14009_38		4.56	1		0	2	MASCOT	T
R14009_38_3,25181	60.43	0	4.53E-08	0|0	R14009_38		4.56	1		0	2	MASCOT	T
R14009_38_3,25213	44.02	0	1.98E-06	0|0	R14009_38		4.56	1		0	2	MASCOT	T
R14009_38_3,25220	56.64	0	1.08E-07	0|0	R14009_38		4.56	1		0	2	MASCOT	T
R14009_38_3,25253	60.16	0	4.82E-08	0|0	R14009_38		4.56	1		0	2	MASCOT	T
R14009_38_3,25254	48.62	0	6.87E-07	0|0	R14009_38		4.56	1		0	2	MASCOT	T
R14009_38_3,25286	58.23	0	7.52E-08	0|0	R14009_38		4.56	1		0	2	MASCOT	T
R14009_38_3,25287	49.31	0	5.86E-07	0|0	R14009_38		4.56	1		0	2	MASCOT	T
R14009_38_3,25319	60.02	0	4.98E-08	0|0	R14009_38		4.56	1		0	2	MASCOT	T
R14009_38_3,25320	44.3	0	1.86E-06	0|0	R14009_38		4.56	1		0	2	MASCOT	T
R14009_38_3,25352	58.24	0	7.50E-08	0|0	R14009_38		4.56	1		0	2	MASCOT	T
R14009_38_3,25353	60.33	0	4.63E-08	0|0	R14009_38		4.56	1		0	2	MASCOT	T
R14009_38_3,25385	60.06	0	4.93E-08	0|0	R14009_38		4.56	1		0	2	MASCOT	T
R14009_38_3,25386	64.05	0	1.97E-08	0|0	R14009_38		4.56	1		0	2	MASCOT	T
R14009_38_3,25418	60.02	0	4.98E-08	0|0	R14009_38		4.56	1		0	2	MASCOT	T
R14009_38_3,25419	63.99	0	2.00E-08	0|0	R14009_38		4.56	1		0	2	MASCOT	T
R14009_38_3,25451	60	0	5.00E-08	0|0	R14009_38		4.56	1		0	2	MASCOT	T
R14009_38_3,25452	64.09	0	1.95E-08	0|0	R14009_38		4.56	1		0	2	MASCOT	T
R14009_38_3,25484	59.52	0	5.58E-08	0|0	R14009_38		4.56	1		0	2	MASCOT	T
R14009_38_3,25485	57.03	0	9.91E-08	0|0	R14009_38		4.56	1		0	2	MASCOT	T
R14009_38_3,25517	60.05	0	4.94E-08	0|0	R14009_38		4.56	1		0	2	MASCOT	T
R14009_38_3,25518	51.2	0	3.79E-07	0|0	R14009_38		4.56	1		0	2	MASCOT	T
R14009_38_3,25550	60.15	0	4.83E-08	0|0	R14009_38		4.56	1		0	2	MASCOT	T
R14009_38_3,25551	68.24	0	7.50E-09	0|0	R14009_38		4.56	1		0	2	MASCOT	T
R14009_38_3,25583	71.85	0	3.27E-09	0|0	R14009_38		4.56	1		0	2	MASCOT	T
R14009_38_3,25584	46.48	0	1.12E-06	0|0	R14009_38		4.56	1		0	2	MASCOT	T
R14009_38_3,25616	60.28	0	4.69E-08	0|0	R14009_38		4.56	1		0	2	MASCOT	T
R14009_38_3,25617	51.17	0	3.82E-07	0|0	R14009_38		4.56	1		0	2	MASCOT	T
R14009_38_3,25649	67.72	0	8.45E-09	0|0	R14009_38		4.56	1		0	2	MASCOT	T
R14009_38_3,25651	51.37	0	3.65E-07	0|0	R14009_38		4.56	1		0	2	MASCOT	T
R14009_38_3,25684	58.43	0	7.18E-08	0|0	R14009_38		4.56	1		0	2	MASCOT	T
R14009_38_3,25685	44.76	0	1.67E-06	0|0	R14009_38		4.56	1		0	2	MASCOT	T
R14009_38_3,25717	62.52	0	2.80E-08	0|0	R14009_38		4.56	1		0	2	MASCOT	T
R14009_38_3,25718	41.92	0	3.21E-06	0|0	R14009_38		4.56	1		0	2	MASCOT	T
R14009_38_3,25750	64.75	0	1.67E-08	0|0	R14009_38		4.56	1		0	2	MASCOT	T
R14009_38_3,25751	43.46	0	2.25E-06	0|0	R14009_38		4.56	1		0	2	MASCOT	T
R14009_38_3,25783	60.64	0	4.31E-08	0|0	R14009_38		4.56	1		0	2	MASCOT	T
R14009_38_3,25785	44.92	0	1.61E-06	0|0	R14009_38		4.56	1		0	2	MASCOT	T
R14009_38_3,25816	71.97	0	3.18E-09	0|0	R14009_38		4.56	1		0	2	MASCOT	T
R14009_38_3,25828	46.16	0	1.21E-06	0|0	R14009_38		4.56	1		0	2	MASCOT	T
R14009_38_3,25850	56.68	0	1.07E-07	0|0	R14009_38		4.56	1		0	2	MASCOT	T

R14009_38_3,25861	K.DGVILLWDLAEGKK.L	1556.86825	-0.0003	3	1	57.86	0	8.18E-08	0	0	R14009_38	4.56	1	0	2	MASCOT	T
R14009_38_3,25882	K.DGVILLWDLAEGKK.L	1556.86825	-0.00003	2	1	69.78	0	5.26E-09	0	0	R14009_38	4.56	1	0	2	MASCOT	T
R14009_38_3,25897	K.DGVILLWDLAEGKK.L	1556.86825	-0.00039	3	1	30.94	0	4.03E-05	0	0	R14009_38	4.56	1	0	2	MASCOT	T
R14009_38_3,25915	K.DGVILLWDLAEGKK.L	1556.86825	0.00019	2	1	63.41	0	2.28E-08	0	0	R14009_38	4.56	1	0	2	MASCOT	T
R14009_38_3,25941	K.DGVILLWDLAEGKK.L	1556.86825	-0.00008	3	1	32.22	0	3.00E-05	0	0	R14009_38	4.56	1	0	2	MASCOT	T
R14009_38_3,25951	K.DGVILLWDLAEGKK.L	1556.86825	0.00065	2	1	76.84	0	1.04E-09	0	0	R14009_38	4.56	1	0	2	MASCOT	T
R14009_38_3,25987	K.DGVILLWDLAEGKK.L	1556.86825	-0.00008	3	1	35.04	0	1.57E-05	0	0	R14009_38	4.56	1	0	2	MASCOT	T
R14009_38_3,25995	K.DGVILLWDLAEGKK.L	1556.86825	0.00119	2	1	67.09	0	9.77E-09	0	0	R14009_38	4.56	1	0	2	MASCOT	T
R14009_38_3,26040	K.DGVILLWDLAEGKK.L	1556.86825	0.00351	2	1	68.29	0	7.41E-09	0	0	R14009_38	4.56	1	0	2	MASCOT	T
R14009_38_3,26085	K.DGVILLWDLAEGKK.L	1556.86825	-0.00757	2	1	49.85	0	5.18E-07	0	0	R14009_38	4.56	1	0	2	MASCOT	T
R14009_38_3,26129	K.DGVILLWDLAEGKK.L	1556.86825	-0.02305	2	1	84.76	0	1.67E-10	0	0	R14009_38	4.56	1	0	2	MASCOT	T
R14009_38_3,26174	K.DGVILLWDLAEGKK.L	1556.86825	-0.02175	2	1	72.66	0	2.71E-09	0	0	R14009_38	4.56	1	0	2	MASCOT	T
R14009_38_3,26219	K.DGVILLWDLAEGKK.L	1556.86825	-0.02347	2	1	42.13	0	3.06E-06	0	0	R14009_38	4.56	1	0	2	MASCOT	T
R14009_38_3,26273	K.DGVILLWDLAEGKK.L	1556.86825	-0.02905	2	1	45.49	0	1.41E-06	0	0	R14009_38	4.56	1	0	2	MASCOT	T
R14009_38_3,26317	K.DGVILLWDLAEGKK.L	1556.86825	-0.02797	2	1	65	0	1.58E-08	0	0	R14009_38	4.56	1	0	2	MASCOT	T
R14009_38_3,26360	K.DGVILLWDLAEGKK.L	1556.86825	-0.02693	2	1	46.43	0	1.14E-06	0	0	R14009_38	4.56	1	0	2	MASCOT	T
R14009_38_3,26413	K.DGVILLWDLAEGKK.L	1556.86825	-0.02051	2	1	49.16	0	6.07E-07	0	0	R14009_38	4.56	1	0	2	MASCOT	T
R14009_38_3,26460	K.DGVILLWDLAEGKK.L	1556.86825	-0.02511	2	1	51.51	0	3.53E-07	0	0	R14009_38	4.56	1	0	2	MASCOT	T
R14009_38_3,26503	K.DGVILLWDLAEGKK.L	1556.86825	-0.02879	2	1	37.93	0	8.05E-06	0	0	R14009_38	4.56	1	0	2	MASCOT	T
R14009_38_3,26547	K.DGVILLWDLAEGKK.L	1556.86825	-0.02829	2	1	48.94	0	6.38E-07	0	0	R14009_38	4.56	1	0	2	MASCOT	T
R14009_38_3,26590	K.DGVILLWDLAEGKK.L	1556.86825	-0.02581	2	1	44.89	0	1.62E-06	0	0	R14009_38	4.56	1	0	2	MASCOT	T
R14009_38_3,26633	K.DGVILLWDLAEGKK.L	1556.86825	-0.02667	2	1	45.74	0	1.33E-06	0	0	R14009_38	4.56	1	0	2	MASCOT	T
R14009_38_3,26680	K.DGVILLWDLAEGKK.L	1556.86825	-0.02539	2	1	64.49	0	1.78E-08	0	0	R14009_38	4.56	1	0	2	MASCOT	T
R14009_38_3,26723	K.DGVILLWDLAEGKK.L	1556.86825	-0.02817	2	1	48.44	0	7.16E-07	0	0	R14009_38	4.56	1	0	2	MASCOT	T
R14009_38_3,26766	K.DGVILLWDLAEGKK.L	1556.86825	-0.02805	2	1	59.97	0	5.03E-08	0	0	R14009_38	4.56	1	0	2	MASCOT	T
R14009_38_3,26808	K.DGVILLWDLAEGKK.L	1556.86825	-0.01789	2	1	57.49	0	8.91E-08	0	0	R14009_38	4.56	1	0	2	MASCOT	T
R14009_38_3,26855	K.DGVILLWDLAEGKK.L	1556.86825	-0.00625	2	1	34.88	0	1.63E-05	0	0	R14009_38	4.56	1	0	2	MASCOT	T
R14009_38_3,26898	K.DGVILLWDLAEGKK.L	1556.86825	-0.00583	2	1	42.66	0	2.71E-06	0	0	R14009_38	4.56	1	0	2	MASCOT	T
R14009_38_3,26955	K.DGVILLWDLAEGKK.L	1556.86825	-0.00295	2	1	36.96	0	1.01E-05	0	0	R14009_38	4.56	1	0	2	MASCOT	T
R14009_38_3,27238	K.DGVILLWDLAEGKK.L	1556.86825	0.00189	2	1	36.52	0	1.11E-05	0	0	R14009_38	4.56	1	0	2	MASCOT	T
R14009_38_3,27623	K.DGVILLWDLAEGKK.L	1556.86825	-0.00111	2	1	44.75	0	1.67E-06	0	0	R14009_38	4.56	1	0	2	MASCOT	T
R14009_38_3,27666	K.DGVILLWDLAEGKK.L	1556.86825	-0.00159	2	1	53.09	0	2.45E-07	0	0	R14009_38	4.56	1	0	2	MASCOT	T
R14009_38_3,27708	K.DGVILLWDLAEGKK.L	1556.86825	-0.00003	2	1	60.08	0	4.91E-08	0	0	R14009_38	4.56	1	0	2	MASCOT	T
R14009_38_3,27749	K.DGVILLWDLAEGKK.L	1556.86825	-0.00011	2	1	57.5	0	8.89E-08	0	0	R14009_38	4.56	1	0	2	MASCOT	T
R14009_38_3,27774	K.DGVILLWDLAEGKK.L	1556.86825	-0.00341	3	1	20.05	0	4.94E-04	0	0	R14009_38	4.56	1	0	2	MASCOT	T
R14009_38_3,27782	K.DGVILLWDLAEGKK.L	1556.86825	-0.00047	2	1	63.98	0	2.00E-08	0	0	R14009_38	4.56	1	0	2	MASCOT	T
R14009_38_3,27814	K.DGVILLWDLAEGKK.L	1556.86825	0.00011	2	1	59.55	0	5.55E-08	0	0	R14009_38	4.56	1	0	2	MASCOT	T
R14009_38_3,27848	K.DGVILLWDLAEGKK.L	1556.86825	-0.00049	2	1	59.08	0	6.18E-08	0	0	R14009_38	4.56	1	0	2	MASCOT	T
R14009_38_3,27881	K.DGVILLWDLAEGKK.L	1556.86825	-0.00031	2	1	67.63	0	8.63E-09	0	0	R14009_38	4.56	1	0	2	MASCOT	T
R14009_38_3,27915	K.DGVILLWDLAEGKK.L	1556.86825	0.00001	2	1	61.65	0	3.42E-08	0	0	R14009_38	4.56	1	0	2	MASCOT	T
R14009_38_3,27948	K.DGVILLWDLAEGKK.L	1556.86825	-0.00021	2	1	66.93	0	1.01E-08	0	0	R14009_38	4.56	1	0	2	MASCOT	T
R14009_38_3,27982	K.DGVILLWDLAEGKK.L	1556.86825	0.00105	2	1	52.76	0	2.65E-07	0	0	R14009_38	4.56	1	0	2	MASCOT	T
R14009_38_3,28019	K.DGVILLWDLAEGKK.L	1556.86825	-0.00133	2	1	28.81	0	6.58E-05	0	0	R14009_38	4.56	1	0	2	MASCOT	T
R14009_38_3,19114	K.DVLSVAFSTDNR.Q	1323.65393	-0.00222	2	1	65.09	0	1.55E-08	0	0	R14009_38	4.21	0	0	2	MASCOT	T
R14009_38_3,19156	K.DVLSVAFSTDNR.Q	1323.65393	-0.0022	2	1	78.96	0	6.35E-10	0	0	R14009_38	4.21	0	0	2	MASCOT	T
R14009_38_3,19200	K.DVLSVAFSTDNR.Q	1323.65393	-0.00016	2	1	71.88	0	3.24E-09	0	0	R14009_38	4.21	0	0	2	MASCOT	T
R14009_38_3,19242	K.DVLSVAFSTDNR.Q	1323.65393	0.00088	2	1	75.58	0	1.38E-09	0	0	R14009_38	4.21	0	0	2	MASCOT	T
R14009_38_3,19284	K.DVLSVAFSTDNR.Q	1323.65393	0.00066	2	1	63.4	0	2.29E-08	0	0	R14009_38	4.21	0	0	2	MASCOT	T
R14009_38_3,19328	K.DVLSVAFSTDNR.Q	1323.65393	0.00012	2	1	82.16	0	3.04E-10	0	0	R14009_38	4.21	0	0	2	MASCOT	T
R14009_38_3,19361	K.DVLSVAFSTDNR.Q	1323.65393	0.00026	2	1	76.17	0	1.21E-09	0	0	R14009_38	4.21	0	0	2	MASCOT	T
R14009_38_3,19395	K.DVLSVAFSTDNR.Q	1323.65393	0.00056	2	1	78.36	0	7.29E-10	0	0	R14009_38	4.21	0	0	2	MASCOT	T
R14009_38_3,19438	K.DVLSVAFSTDNR.Q	1323.65393	0.00094	2	1	65.75	0	1.33E-08	0	0	R14009_38	4.21	0	0	2	MASCOT	T
R14009_38_3,19470	K.DVLSVAFSTDNR.Q	1323.65393	0.00034	2	1	76.66	0	1.08E-09	0	0	R14009_38	4.21	0	0	2	MASCOT	T
R14009_38_3,19503	K.DVLSVAFSTDNR.Q	1323.65393	0.00092	2	1	75.5	0	1.41E-09	0	0	R14009_38	4.21	0	0	2	MASCOT	T
R14009_38_3,19536	K.DVLSVAFSTDNR.Q	1323.65393	0.00048	2	1	75.44	0	1.43E-09	0	0	R14009_38	4.21	0	0	2	MASCOT	T
R14009_38_3,19568	K.DVLSVAFSTDNR.Q	1323.65393	0.0003	2	1	75.02	0	1.57E-09	0	0	R14009_38	4.21	0	0	2	MASCOT	T
R14009_38_3,19601	K.DVLSVAFSTDNR.Q	1323.65393	0.0001	2	1	75.5	0	1.41E-09	0	0	R14009_38	4.21	0	0	2	MASCOT	T
R14009_38_3,19634	K.DVLSVAFSTDNR.Q	1323.65393	0.00048	2	1	63.31	0	2.33E-08	0	0	R14009_38	4.21	0	0	2	MASCOT	T
R14009_38_3,19667	K.DVLSVAFSTDNR.Q	1323.65393	0.00092	2	1	71.33	0	3.68E-09	0	0	R14009_38	4.21	0	0	2	MASCOT	T
R14009_38_3,19700	K.DVLSVAFSTDNR.Q	1323.65393	0.00116	2	1	76.67	0	1.08E-09	0	0	R14009_38	4.21	0	0	2	MASCOT	T
R14009_38_3,19734	K.DVLSVAFSTDNR.Q	1323.65393	0.00178	2	1	64.24	0	1.88E-08	0	0	R14009_38	4.21	0	0	2	MASCOT	T
R14009_38_3,19767	K.DVLSVAFSTDNR.Q	1323.65393	0.00136	2	1	68.98	0	6.32E-09	0	0	R14009_38	4.21	0	0	2	MASCOT	T
R14009_38_3,19798	K.DVLSVAFSTDNR.Q	1323.65393	0.00124	2	1	62.9	0	2.56E-08	0	0	R14009_38	4.21	0	0	2	MASCOT	T
R14009_38_3,19831	K.DVLSVAFSTDNR.Q	1323.65393	0.0017	2	1	63.84	0	2.07E-08	0	0	R14009_38	4.21	0	0	2	MASCOT	T
R14009_38_3,19864	K.DVLSVAFSTDNR.Q	1323.65393	0.00178	2	1	65.68	0	1.35E-08	0	0	R14009_38	4.21	0	0	2	MASCOT	T
R14009_38_3,19897	K.DVLSVAFSTDNR.Q	1323.65393	0.0017	2	1	61.16	0	3.83E-08	0	0	R14009_38	4.21	0	0	2	MASCOT	T
R14009_38_3,19930	K.DVLSVAFSTDNR.Q	1323.65393	0.00194	2	1	61.47	0	3.56E-08	0	0	R14009_38	4.21	0	0	2	MASCOT	T
R14009_38_3,19963	K.DVLSVAFSTDNR.Q	1323.65393	0.00156	2	1	61.21	0	3.78E-08	0	0	R14009_38	4.21	0	0	2	MASCOT	T
R14009_38_3,19996	K.DVLSVAFSTDNR.Q	1323.65393	0.00116	2	1	74.94	0	1.60E-09	0	0	R14009_38	4.21	0	0	2	MASCOT	T
R14009_38_3,20029	K.DVLSVAFSTDNR.Q	1323.65393	0.00092	2	1	76.42	0	1.14E-09	0	0	R14009_38	4.21	0	0	2	MASCOT	T
R14009_38_3,20062	K.DVLSVAFSTDNR.Q	1323.65393	0.00162	2	1	76.67	0	1.08E-09	0	0	R14009_38	4.21	0	0	2	MASCOT	T
R14009_38_3,20095	K.DVLSVAFSTDNR.Q	1323.65393	0.00128	2	1	62.87	0	2.58E-08	0	0	R14009_38	4.21	0	0	2	MASCOT	T
R14009_38_3,20128	K.DVLSVAFSTDNR.Q	1323.65393	0.00144	2	1	65.48	0	1.42E-08	0	0	R14009_38	4.21	0	0	2	MASCOT	T

R14009_38_3,20161	K.DVLSVAFSTDNR.Q	1323.65393	0.00126	2	1	63.36	0	2.31E-08	0	0	R14009_38	4.21	0	0	2	MASCOT	T
R14009_38_3,20195	K.DVLSVAFSTDNR.Q	1323.65393	0.0004	2	1	61.15	0	3.84E-08	0	0	R14009_38	4.21	0	0	2	MASCOT	T
R14009_38_3,20228	K.DVLSVAFSTDNR.Q	1323.65393	0.00046	2	1	65.72	0	1.34E-08	0	0	R14009_38	4.21	0	0	2	MASCOT	T
R14009_38_3,20262	K.DVLSVAFSTDNR.Q	1323.65393	0.00092	2	1	74.99	0	1.58E-09	0	0	R14009_38	4.21	0	0	2	MASCOT	T
R14009_38_3,20295	K.DVLSVAFSTDNR.Q	1323.65393	0.00036	2	1	68.98	0	6.32E-09	0	0	R14009_38	4.21	0	0	2	MASCOT	T
R14009_38_3,20329	K.DVLSVAFSTDNR.Q	1323.65393	0.001	2	1	74.97	0	1.59E-09	0	0	R14009_38	4.21	0	0	2	MASCOT	T
R14009_38_3,20364	K.DVLSVAFSTDNR.Q	1323.65393	0.00036	2	1	76.47	0	1.13E-09	0	0	R14009_38	4.21	0	0	2	MASCOT	T
R14009_38_3,20408	K.DVLSVAFSTDNR.Q	1323.65393	0.00054	2	1	82.08	0	3.10E-10	0	0	R14009_38	4.21	0	0	2	MASCOT	T
R14009_38_3,20449	K.DVLSVAFSTDNR.Q	1323.65393	0.00066	2	1	64.93	0	1.61E-08	0	0	R14009_38	4.21	0	0	2	MASCOT	T
R14009_38_3,20482	K.DVLSVAFSTDNR.Q	1323.65393	0.00042	2	1	71.35	0	3.66E-09	0	0	R14009_38	4.21	0	0	2	MASCOT	T
R14009_38_3,20516	K.DVLSVAFSTDNR.Q	1323.65393	-0.00044	2	1	79.31	0	5.86E-10	0	0	R14009_38	4.21	0	0	2	MASCOT	T
R14009_38_3,20550	K.DVLSVAFSTDNR.Q	1323.65393	0.00076	2	1	75.41	0	1.44E-09	0	0	R14009_38	4.21	0	0	2	MASCOT	T
R14009_38_3,20638	K.DVLSVAFSTDNR.Q	1323.65393	0.0016	2	1	69.32	0	5.85E-09	0	0	R14009_38	4.21	0	0	2	MASCOT	T
R14009_38_3,20682	K.DVLSVAFSTDNR.Q	1323.65393	0.00252	2	1	64.15	0	1.92E-08	0	0	R14009_38	4.21	0	0	2	MASCOT	T
R14009_38_3,20726	K.DVLSVAFSTDNR.Q	1323.65393	0.00176	2	1	78.19	0	7.59E-10	0	0	R14009_38	4.21	0	0	2	MASCOT	T
R14009_38_3,20771	K.DVLSVAFSTDNR.Q	1323.65393	0.0017	2	1	78.14	0	7.67E-10	0	0	R14009_38	4.21	0	0	2	MASCOT	T
R14009_38_3,20816	K.DVLSVAFSTDNR.Q	1323.65393	0.00162	2	1	64.99	0	1.58E-08	0	0	R14009_38	4.21	0	0	2	MASCOT	T
R14009_38_3,20859	K.DVLSVAFSTDNR.Q	1323.65393	0.0009	2	1	67.16	0	9.62E-09	0	0	R14009_38	4.21	0	0	2	MASCOT	T
R14009_38_3,20913	K.DVLSVAFSTDNR.Q	1323.65393	-0.00022	2	1	78.17	0	7.62E-10	0	0	R14009_38	4.21	0	0	2	MASCOT	T
R14009_38_3,21291	K.DVLSVAFSTDNR.Q	1323.65393	-0.00144	2	1	76.71	0	1.07E-09	0	0	R14009_38	4.21	0	0	2	MASCOT	T
R14009_38_3,21365	K.DVLSVAFSTDNR.Q	1323.65393	-0.00088	2	1	78.19	0	7.59E-10	0	0	R14009_38	4.21	0	0	2	MASCOT	T
R14009_38_3,21466	K.DVLSVAFSTDNR.Q	1323.65393	-0.00044	2	1	71.73	0	3.36E-09	0	0	R14009_38	4.21	0	0	2	MASCOT	T
R14009_38_3,21510	K.DVLSVAFSTDNR.Q	1323.65393	-0.00032	2	1	79.89	0	5.13E-10	0	0	R14009_38	4.21	0	0	2	MASCOT	T
R14009_38_3,21552	K.DVLSVAFSTDNR.Q	1323.65393	0.00024	2	1	70.67	0	4.29E-09	0	0	R14009_38	4.21	0	0	2	MASCOT	T
R14009_38_3,21595	K.DVLSVAFSTDNR.Q	1323.65393	0.00044	2	1	74.19	0	1.91E-09	0	0	R14009_38	4.21	0	0	2	MASCOT	T
R14009_38_3,21639	K.DVLSVAFSTDNR.Q	1323.65393	0.00022	2	1	71.88	0	3.24E-09	0	0	R14009_38	4.21	0	0	2	MASCOT	T
R14009_38_3,21685	K.DVLSVAFSTDNR.Q	1323.65393	0.00004	2	1	83.87	0	2.05E-10	0	0	R14009_38	4.21	0	0	2	MASCOT	T
R14009_38_3,21729	K.DVLSVAFSTDNR.Q	1323.65393	0.00038	2	1	67.92	0	8.07E-09	0	0	R14009_38	4.21	0	0	2	MASCOT	T
R14009_38_3,21774	K.DVLSVAFSTDNR.Q	1323.65393	0.00004	2	1	74.82	0	1.65E-09	0	0	R14009_38	4.21	0	0	2	MASCOT	T
R14009_38_3,21817	K.DVLSVAFSTDNR.Q	1323.65393	-0.00012	2	1	73.02	0	2.49E-09	0	0	R14009_38	4.21	0	0	2	MASCOT	T
R14009_38_3,21861	K.DVLSVAFSTDNR.Q	1323.65393	0.00004	2	1	75.55	0	1.39E-09	0	0	R14009_38	4.21	0	0	2	MASCOT	T
R14009_38_3,21906	K.DVLSVAFSTDNR.Q	1323.65393	0.00062	2	1	66.46	0	1.13E-08	0	0	R14009_38	4.21	0	0	2	MASCOT	T
R14009_38_3,21950	K.DVLSVAFSTDNR.Q	1323.65393	-0.00028	2	1	68.42	0	7.19E-09	0	0	R14009_38	4.21	0	0	2	MASCOT	T
R14009_38_3,21993	K.DVLSVAFSTDNR.Q	1323.65393	-0.0003	2	1	78.42	0	7.19E-10	0	0	R14009_38	4.21	0	0	2	MASCOT	T
R14009_38_3,22036	K.DVLSVAFSTDNR.Q	1323.65393	-0.00016	2	1	83.36	0	2.31E-10	0	0	R14009_38	4.21	0	0	2	MASCOT	T
R14009_38_3,22079	K.DVLSVAFSTDNR.Q	1323.65393	0.00056	2	1	81.27	0	3.73E-10	0	0	R14009_38	4.21	0	0	2	MASCOT	T
R14009_38_3,22123	K.DVLSVAFSTDNR.Q	1323.65393	0.0008	2	1	73.81	0	2.08E-09	0	0	R14009_38	4.21	0	0	2	MASCOT	T
R14009_38_3,22168	K.DVLSVAFSTDNR.Q	1323.65393	0.0002	2	1	81.79	0	3.31E-10	0	0	R14009_38	4.21	0	0	2	MASCOT	T
R14009_38_3,22212	K.DVLSVAFSTDNR.Q	1323.65393	0.00062	2	1	73.09	0	2.45E-09	0	0	R14009_38	4.21	0	0	2	MASCOT	T
R14009_38_3,22256	K.DVLSVAFSTDNR.Q	1323.65393	0.00014	2	1	76.39	0	1.15E-09	0	0	R14009_38	4.21	0	0	2	MASCOT	T
R14009_38_3,22301	K.DVLSVAFSTDNR.Q	1323.65393	0.00044	2	1	75.2	0	1.51E-09	0	0	R14009_38	4.21	0	0	2	MASCOT	T
R14009_38_3,22346	K.DVLSVAFSTDNR.Q	1323.65393	0.00028	2	1	85.74	0	1.33E-10	0	0	R14009_38	4.21	0	0	2	MASCOT	T
R14009_38_3,22389	K.DVLSVAFSTDNR.Q	1323.65393	0.00014	2	1	78.4	0	7.23E-10	0	0	R14009_38	4.21	0	0	2	MASCOT	T
R14009_38_3,22435	K.DVLSVAFSTDNR.Q	1323.65393	-0.00044	2	1	73.36	0	2.31E-09	0	0	R14009_38	4.21	0	0	2	MASCOT	T
R14009_38_3,22480	K.DVLSVAFSTDNR.Q	1323.65393	0.00004	2	1	86.8	0	1.04E-10	0	0	R14009_38	4.21	0	0	2	MASCOT	T
R14009_38_3,22524	K.DVLSVAFSTDNR.Q	1323.65393	-0.00012	2	1	74.11	0	1.94E-09	0	0	R14009_38	4.21	0	0	2	MASCOT	T
R14009_38_3,22566	K.DVLSVAFSTDNR.Q	1323.65393	-0.00022	2	1	85.82	0	1.31E-10	0	0	R14009_38	4.21	0	0	2	MASCOT	T
R14009_38_3,22610	K.DVLSVAFSTDNR.Q	1323.65393	-0.00086	2	1	70.7	0	4.26E-09	0	0	R14009_38	4.21	0	0	2	MASCOT	T
R14009_38_3,22653	K.DVLSVAFSTDNR.Q	1323.65393	0	2	1	78.82	0	6.56E-10	0	0	R14009_38	4.21	0	0	2	MASCOT	T
R14009_38_3,22698	K.DVLSVAFSTDNR.Q	1323.65393	0.00036	2	1	70.73	0	4.23E-09	0	0	R14009_38	4.21	0	0	2	MASCOT	T
R14009_38_3,22741	K.DVLSVAFSTDNR.Q	1323.65393	0.00036	2	1	72.63	0	2.73E-09	0	0	R14009_38	4.21	0	0	2	MASCOT	T
R14009_38_3,22785	K.DVLSVAFSTDNR.Q	1323.65393	-0.00008	2	1	79.28	0	5.90E-10	0	0	R14009_38	4.21	0	0	2	MASCOT	T
R14009_38_3,22830	K.DVLSVAFSTDNR.Q	1323.65393	0.00046	2	1	70.78	0	4.18E-09	0	0	R14009_38	4.21	0	0	2	MASCOT	T
R14009_38_3,22875	K.DVLSVAFSTDNR.Q	1323.65393	-0.0007	2	1	64.62	0	1.73E-08	0	0	R14009_38	4.21	0	0	2	MASCOT	T
R14009_38_3,22920	K.DVLSVAFSTDNR.Q	1323.65393	-0.00002	2	1	95.65	0	1.36E-11	0	0	R14009_38	4.21	0	0	2	MASCOT	T
R14009_38_3,22962	K.DVLSVAFSTDNR.Q	1323.65393	0.00038	2	1	73.11	0	2.44E-09	0	0	R14009_38	4.21	0	0	2	MASCOT	T
R14009_38_3,23344	K.DVLSVAFSTDNR.Q	1323.65393	0.00208	2	1	74.47	0	1.79E-09	0	0	R14009_38	4.21	0	0	2	MASCOT	T
R14009_38_3,23403	K.DVLSVAFSTDNR.Q	1323.65393	0.00164	2	1	78.81	0	6.58E-10	0	0	R14009_38	4.21	0	0	2	MASCOT	T
R14009_38_3,23445	K.DVLSVAFSTDNR.Q	1323.65393	0.00186	2	1	66.73	0	1.06E-08	0	0	R14009_38	4.21	0	0	2	MASCOT	T
R14009_38_3,23490	K.DVLSVAFSTDNR.Q	1323.65393	0.00046	2	1	83.63	0	2.17E-10	0	0	R14009_38	4.21	0	0	2	MASCOT	T
R14009_38_3,23532	K.DVLSVAFSTDNR.Q	1323.65393	-0.00024	2	1	85.39	0	1.45E-10	0	0	R14009_38	4.21	0	0	2	MASCOT	T
R14009_38_3,23578	K.DVLSVAFSTDNR.Q	1323.65393	0.00002	2	1	70.98	0	3.99E-09	0	0	R14009_38	4.21	0	0	2	MASCOT	T
R14009_38_3,23623	K.DVLSVAFSTDNR.Q	1323.65393	0.00026	2	1	81.21	0	3.78E-10	0	0	R14009_38	4.21	0	0	2	MASCOT	T
R14009_38_3,23675	K.DVLSVAFSTDNR.Q	1323.65393	0.00024	2	1	84.78	0	1.66E-10	0	0	R14009_38	4.21	0	0	2	MASCOT	T
R14009_38_3,23720	K.DVLSVAFSTDNR.Q	1323.65393	0.00062	2	1	77.2	0	9.53E-10	0	0	R14009_38	4.21	0	0	2	MASCOT	T
R14009_38_3,23767	K.DVLSVAFSTDNR.Q	1323.65393	0.00016	2	1	87.8	0	8.30E-11	0	0	R14009_38	4.21	0	0	2	MASCOT	T
R14009_38_3,23809	K.DVLSVAFSTDNR.Q	1323.65393	-0.00004	2	1	85.82	0	1.31E-10	0	0	R14009_38	4.21	0	0	2	MASCOT	T
R14009_38_3,23855	K.DVLSVAFSTDNR.Q	1323.65393	-0.00118	2	1	81.38	0	3.64E-10	0	0	R14009_38	4.21	0	0	2	MASCOT	T
R14009_38_3,23898	K.DVLSVAFSTDNR.Q	1323.65393	-0.00074	2	1	78.62	0	6.87E-10	0	0	R14009_38	4.21	0	0	2	MASCOT	T
R14009_38_3,23942	K.DVLSVAFSTDNR.Q	1323.65393	0.0005	2	1	74.02	0	1.98E-09	0	0	R14009_38	4.21	0	0	2	MASCOT	T
R14009_38_3,23987	K.DVLSVAFSTDNR.Q	1323.65393	-0.00044	2	1	71.63	0	3.44E-09	0	0	R14009_38	4.21	0	0	2	MASCOT	T
R14009_38_3,24064	K.DVLSVAFSTDNR.Q	1323.65393	-0.00056	2	1	66.92	0	1.02E-08	0	0	R14009_38	4.21	0	0	2	MASCOT	T
R14009_38_3,24102	K.DVLSVAFSTDNR.Q	1323.65393	0.00014	2	1	66.04	0	1.24E-08	0	0	R14009_38	4.21	0	0	2	MASCOT	T

R14009_38_3,24139	K.DVLSVAFSTDNR.Q	1323.65393	-0.00012	2	1	46.12	0	1.22E-06	0	0	R14009_38	4.21	0	0	2	MASCOT	T
R14009_38_3,24183	K.DVLSVAFSTDNR.Q	1323.65393	0.00022	2	1	57.67	0	8.55E-08	0	0	R14009_38	4.21	0	0	2	MASCOT	T
R14009_38_3,24227	K.DVLSVAFSTDNR.Q	1323.65393	-0.00006	2	1	88.36	0	7.29E-11	0	0	R14009_38	4.21	0	0	2	MASCOT	T
R14009_38_3,24268	K.DVLSVAFSTDNR.Q	1323.65393	-0.00058	2	1	53.25	0	2.37E-07	0	0	R14009_38	4.21	0	0	2	MASCOT	T
R14009_38_3,24315	K.DVLSVAFSTDNR.Q	1323.65393	0.00014	2	1	76.97	0	1.00E-09	0	0	R14009_38	4.21	0	0	2	MASCOT	T
R14009_38_3,24360	K.DVLSVAFSTDNR.Q	1323.65393	0.00006	2	1	62	0	3.15E-08	0	0	R14009_38	4.21	0	0	2	MASCOT	T
R14009_38_3,24404	K.DVLSVAFSTDNR.Q	1323.65393	0	2	1	61.38	0	3.64E-08	0	0	R14009_38	4.21	0	0	2	MASCOT	T
R14009_38_3,24448	K.DVLSVAFSTDNR.Q	1323.65393	-0.00108	2	1	65.56	0	1.39E-08	0	0	R14009_38	4.21	0	0	2	MASCOT	T
R14009_38_3,24492	K.DVLSVAFSTDNR.Q	1323.65393	-0.0013	2	1	51.18	0	3.81E-07	0	0	R14009_38	4.21	0	0	2	MASCOT	T
R14009_38_3,24532	K.DVLSVAFSTDNR.Q	1323.65393	-0.00096	2	1	74.04	0	1.97E-09	0	0	R14009_38	4.21	0	0	2	MASCOT	T
R14009_38_3,24580	K.DVLSVAFSTDNR.Q	1323.65393	0.00056	2	1	59.47	0	5.65E-08	0	0	R14009_38	4.21	0	0	2	MASCOT	T
R14009_38_3,24623	K.DVLSVAFSTDNR.Q	1323.65393	0	2	1	63.38	0	2.30E-08	0	0	R14009_38	4.21	0	0	2	MASCOT	T
R14009_38_3,24667	K.DVLSVAFSTDNR.Q	1323.65393	-0.00018	2	1	62.72	0	2.67E-08	0	0	R14009_38	4.21	0	0	2	MASCOT	T
R14009_38_3,24710	K.DVLSVAFSTDNR.Q	1323.65393	0.00048	2	1	45.77	0	1.32E-06	0	0	R14009_38	4.21	0	0	2	MASCOT	T
R14009_38_3,24754	K.DVLSVAFSTDNR.Q	1323.65393	0.00228	2	1	60.07	0	4.92E-08	0	0	R14009_38	4.21	0	0	2	MASCOT	T
R14009_38_3,24798	K.DVLSVAFSTDNR.Q	1323.65393	0.0011	2	1	60.87	0	4.09E-08	0	0	R14009_38	4.21	0	0	2	MASCOT	T
R14009_38_3,24842	K.DVLSVAFSTDNR.Q	1323.65393	0.00098	2	1	71.89	0	3.24E-09	0	0	R14009_38	4.21	0	0	2	MASCOT	T
R14009_38_3,24885	K.DVLSVAFSTDNR.Q	1323.65393	-0.00024	2	1	55.66	0	1.36E-07	0	0	R14009_38	4.21	0	0	2	MASCOT	T
R14009_38_3,24932	K.DVLSVAFSTDNR.Q	1323.65393	0.00054	2	1	68.97	0	6.34E-09	0	0	R14009_38	4.21	0	0	2	MASCOT	T
R14009_38_3,24975	K.DVLSVAFSTDNR.Q	1323.65393	-0.00032	2	1	66.04	0	1.24E-08	0	0	R14009_38	4.21	0	0	2	MASCOT	T
R14009_38_3,25017	K.DVLSVAFSTDNR.Q	1323.65393	-0.00088	2	1	54.42	0	1.81E-07	0	0	R14009_38	4.21	0	0	2	MASCOT	T
R14009_38_3,25061	K.DVLSVAFSTDNR.Q	1323.65393	-0.00082	2	1	58.79	0	6.61E-08	0	0	R14009_38	4.21	0	0	2	MASCOT	T
R14009_38_3,25105	K.DVLSVAFSTDNR.Q	1323.65393	-0.00026	2	1	63.42	0	2.27E-08	0	0	R14009_38	4.21	0	0	2	MASCOT	T
R14009_38_3,25150	K.DVLSVAFSTDNR.Q	1323.65393	-0.00062	2	1	51.18	0	3.81E-07	0	0	R14009_38	4.21	0	0	2	MASCOT	T
R14009_38_3,25193	K.DVLSVAFSTDNR.Q	1323.65393	-0.0006	2	1	76.67	0	1.08E-09	0	0	R14009_38	4.21	0	0	2	MASCOT	T
R14009_38_3,25236	K.DVLSVAFSTDNR.Q	1323.65393	-0.00094	2	1	54.02	0	1.98E-07	0	0	R14009_38	4.21	0	0	2	MASCOT	T
R14009_38_3,25280	K.DVLSVAFSTDNR.Q	1323.65393	0.00022	2	1	68.04	0	7.85E-09	0	0	R14009_38	4.21	0	0	2	MASCOT	T
R14009_38_3,25325	K.DVLSVAFSTDNR.Q	1323.65393	-0.0003	2	1	53.18	0	2.40E-07	0	0	R14009_38	4.21	0	0	2	MASCOT	T
R14009_38_3,25370	K.DVLSVAFSTDNR.Q	1323.65393	0.00022	2	1	81.92	0	3.21E-10	0	0	R14009_38	4.21	0	0	2	MASCOT	T
R14009_38_3,25414	K.DVLSVAFSTDNR.Q	1323.65393	0.00036	2	1	41.75	0	3.34E-06	0	0	R14009_38	4.21	0	0	2	MASCOT	T
R14009_38_3,25457	K.DVLSVAFSTDNR.Q	1323.65393	0.00014	2	1	51.02	0	3.95E-07	0	0	R14009_38	4.21	0	0	2	MASCOT	T
R14009_38_3,25502	K.DVLSVAFSTDNR.Q	1323.65393	-0.00022	2	1	62.73	0	2.67E-08	0	0	R14009_38	4.21	0	0	2	MASCOT	T
R14009_38_3,25545	K.DVLSVAFSTDNR.Q	1323.65393	-0.00074	2	1	56.29	0	1.17E-07	0	0	R14009_38	4.21	0	0	2	MASCOT	T
R14009_38_3,25588	K.DVLSVAFSTDNR.Q	1323.65393	-0.00068	2	1	66.03	0	1.25E-08	0	0	R14009_38	4.21	0	0	2	MASCOT	T
R14009_38_3,25632	K.DVLSVAFSTDNR.Q	1323.65393	-0.00052	2	1	61.62	0	3.44E-08	0	0	R14009_38	4.21	0	0	2	MASCOT	T
R14009_38_3,25676	K.DVLSVAFSTDNR.Q	1323.65393	-0.00038	2	1	55.28	0	1.48E-07	0	0	R14009_38	4.21	0	0	2	MASCOT	T
R14009_38_3,25721	K.DVLSVAFSTDNR.Q	1323.65393	-0.00032	2	1	56.78	0	1.05E-07	0	0	R14009_38	4.21	0	0	2	MASCOT	T
R14009_38_3,25767	K.DVLSVAFSTDNR.Q	1323.65393	-0.00042	2	1	64.97	0	1.59E-08	0	0	R14009_38	4.21	0	0	2	MASCOT	T
R14009_38_3,25811	K.DVLSVAFSTDNR.Q	1323.65393	-0.00046	2	1	52.39	0	2.88E-07	0	0	R14009_38	4.21	0	0	2	MASCOT	T
R14009_38_3,25854	K.DVLSVAFSTDNR.Q	1323.65393	-0.00004	2	1	55.35	0	1.46E-07	0	0	R14009_38	4.21	0	0	2	MASCOT	T
R14009_38_3,25908	K.DVLSVAFSTDNR.Q	1323.65393	-0.00022	2	1	65.93	0	1.28E-08	0	0	R14009_38	4.21	0	0	2	MASCOT	T
R14009_38_3,25955	K.DVLSVAFSTDNR.Q	1323.65393	0.00074	2	1	50.48	0	4.48E-07	0	0	R14009_38	4.21	0	0	2	MASCOT	T
R14009_38_3,25999	K.DVLSVAFSTDNR.Q	1323.65393	0.00176	2	1	67.22	0	9.48E-09	0	0	R14009_38	4.21	0	0	2	MASCOT	T
R14009_38_3,26043	K.DVLSVAFSTDNR.Q	1323.65393	0.0017	2	1	58.26	0	7.46E-08	0	0	R14009_38	4.21	0	0	2	MASCOT	T
R14009_38_3,26086	K.DVLSVAFSTDNR.Q	1323.65393	-0.00468	2	1	67.14	0	9.66E-09	0	0	R14009_38	4.21	0	0	2	MASCOT	T
R14009_38_3,26131	K.DVLSVAFSTDNR.Q	1323.65393	-0.01832	2	1	77.98	0	7.96E-10	0	0	R14009_38	4.21	0	0	2	MASCOT	T
R14009_38_3,26173	K.DVLSVAFSTDNR.Q	1323.65393	-0.01724	2	1	55.21	0	1.51E-07	0	0	R14009_38	4.21	0	0	2	MASCOT	T
R14009_38_3,26218	K.DVLSVAFSTDNR.Q	1323.65393	-0.01888	2	1	65.19	0	1.51E-08	0	0	R14009_38	4.21	0	0	2	MASCOT	T
R14009_38_3,26262	K.DVLSVAFSTDNR.Q	1323.65393	-0.02374	2	1	76.1	0	1.23E-09	0	0	R14009_38	4.21	0	0	2	MASCOT	T
R14009_38_3,26307	K.DVLSVAFSTDNR.Q	1323.65393	-0.023	2	1	52.42	0	2.86E-07	0	0	R14009_38	4.21	0	0	2	MASCOT	T
R14009_38_3,26348	K.DVLSVAFSTDNR.Q	1323.65393	-0.02366	2	1	59.97	0	5.03E-08	0	0	R14009_38	4.21	0	0	2	MASCOT	T
R14009_38_3,26392	K.DVLSVAFSTDNR.Q	1323.65393	-0.02468	2	1	54.34	0	1.84E-07	0	0	R14009_38	4.21	0	0	2	MASCOT	T
R14009_38_3,26438	K.DVLSVAFSTDNR.Q	1323.65393	-0.02428	2	1	68.67	0	6.79E-09	0	0	R14009_38	4.21	0	0	2	MASCOT	T
R14009_38_3,26480	K.DVLSVAFSTDNR.Q	1323.65393	-0.0194	2	1	56.88	0	1.03E-07	0	0	R14009_38	4.21	0	0	2	MASCOT	T
R14009_38_3,26612	K.DVLSVAFSTDNR.Q	1323.65393	-0.0256	2	1	56.44	0	1.13E-07	0	0	R14009_38	4.21	0	0	2	MASCOT	T
R14009_38_3,26670	K.DVLSVAFSTDNR.Q	1323.65393	-0.02474	2	1	45.12	0	1.54E-06	0	0	R14009_38	4.21	0	0	2	MASCOT	T
R14009_38_3,26724	K.DVLSVAFSTDNR.Q	1323.65393	-0.02248	2	1	40.03	0	4.97E-06	0	0	R14009_38	4.21	0	0	2	MASCOT	T
R14009_38_3,26765	K.DVLSVAFSTDNR.Q	1323.65393	-0.02296	2	1	45.61	0	1.37E-06	0	0	R14009_38	4.21	0	0	2	MASCOT	T
R14009_38_3,26806	K.DVLSVAFSTDNR.Q	1323.65393	-0.01484	2	1	57.13	0	9.68E-08	0	0	R14009_38	4.21	0	0	2	MASCOT	T
R14009_38_3,26842	K.DVLSVAFSTDNR.Q	1323.65393	-0.00532	2	1	55.79	0	1.32E-07	0	0	R14009_38	4.21	0	0	2	MASCOT	T
R14009_38_3,26888	K.DVLSVAFSTDNR.Q	1323.65393	-0.0029	2	1	70.45	0	4.51E-09	0	0	R14009_38	4.21	0	0	2	MASCOT	T
R14009_38_3,26932	K.DVLSVAFSTDNR.Q	1323.65393	-0.00266	2	1	41.68	0	3.40E-06	0	0	R14009_38	4.21	0	0	2	MASCOT	T
R14009_38_3,26972	K.DVLSVAFSTDNR.Q	1323.65393	0.002	2	1	42.34	0	2.92E-06	0	0	R14009_38	4.21	0	0	2	MASCOT	T
R14009_38_3,27018	K.DVLSVAFSTDNR.Q	1323.65393	-0.0052	2	1	55.61	0	1.37E-07	0	0	R14009_38	4.21	0	0	2	MASCOT	T
R14009_38_3,27061	K.DVLSVAFSTDNR.Q	1323.65393	-0.00414	2	1	64.78	0	1.66E-08	0	0	R14009_38	4.21	0	0	2	MASCOT	T
R14009_38_3,27106	K.DVLSVAFSTDNR.Q	1323.65393	-0.00512	2	1	58.5	0	7.06E-08	0	0	R14009_38	4.21	0	0	2	MASCOT	T
R14009_38_3,27151	K.DVLSVAFSTDNR.Q	1323.65393	-0.0049	2	1	61.38	0	3.64E-08	0	0	R14009_38	4.21	0	0	2	MASCOT	T
R14009_38_3,27193	K.DVLSVAFSTDNR.Q	1323.65393	0.00158	2	1	55.07	0	1.56E-07	0	0	R14009_38	4.21	0	0	2	MASCOT	T
R14009_38_3,27236	K.DVLSVAFSTDNR.Q	1323.65393	0.00266	2	1	44.32	0	1.85E-06	0	0	R14009_38	4.21	0	0	2	MASCOT	T
R14009_38_3,27284	K.DVLSVAFSTDNR.Q	1323.65393	0.00198	2	1	58.53	0	7.01E-08	0	0	R14009_38	4.21	0	0	2	MASCOT	T
R14009_38_3,27327	K.DVLSVAFSTDNR.Q	1323.65393	0.00102	2	1	65.8	0	1.32E-08	0	0	R14009_38	4.21	0	0	2	MASCOT	T
R14009_38_3,27370	K.DVLSVAFSTDNR.Q	1323.65393	0.00036	2	1	49.32	0	5.85E-07	0	0	R14009_38	4.21	0	0	2	MASCOT	T
R14009_38_3,27545	K.DVLSVAFSTDNR.Q	1323.65393	0.00064	2	1	44.5	0	1.77E-06	0	0	R14009_38	4.21	0	0	2	MASCOT	T

R14009_38_3,27587	K.DVLSVAFSTDNR.Q	1323.65393	0.00008	2	1	48.89	0	6.46E-07	0	0	R14009_38	4.21	0		0	2	MASCOT	T
R14009_38_3,27634	K.DVLSVAFSTDNR.Q	1323.65393	-0.0005	2	1	59.48	0	5.64E-08	0	0	R14009_38	4.21	0		0	2	MASCOT	T
R14009_38_3,27678	K.DVLSVAFSTDNR.Q	1323.65393	-0.00042	2	1	55.81	0	1.31E-07	0	0	R14009_38	4.21	0		0	2	MASCOT	T
R14009_38_3,27718	K.DVLSVAFSTDNR.Q	1323.65393	0.00036	2	1	52.98	0	2.52E-07	0	0	R14009_38	4.21	0		0	2	MASCOT	T
R14009_38_3,27755	K.DVLSVAFSTDNR.Q	1323.65393	-0.00074	2	1	47.24	0	9.44E-07	0	0	R14009_38	4.21	0		0	2	MASCOT	T
R14009_38_3,27800	K.DVLSVAFSTDNR.Q	1323.65393	-0.00004	2	1	41.84	0	3.27E-06	0	0	R14009_38	4.21	0		0	2	MASCOT	T
R14009_38_3,27842	K.DVLSVAFSTDNR.Q	1323.65393	-0.00114	2	1	62.15	0	3.05E-08	0	0	R14009_38	4.21	0		0	2	MASCOT	T
R14009_38_3,27896	K.DVLSVAFSTDNR.Q	1323.65393	0.0003	2	1	33.13	0	2.43E-05	0	0	R14009_38	4.21	0		0	2	MASCOT	T
R14009_38_3,27943	K.DVLSVAFSTDNR.Q	1323.65393	-0.00012	2	1	45.88	0	1.29E-06	0	0	R14009_38	4.21	0		0	2	MASCOT	T
R14009_38_3,27985	K.DVLSVAFSTDNR.Q	1323.65393	0.00074	2	1	53	0	2.51E-07	0	0	R14009_38	4.21	0		0	2	MASCOT	T
R14009_38_3,28032	K.DVLSVAFSTDNR.Q	1323.65393	0.0037	2	1	46.57	0	1.10E-06	0	0	R14009_38	4.21	0		0	2	MASCOT	T
R14009_38_3,6731	K.EDK#SYGVAQR.R	1478.68699	0.00141	2	1	61.16	0	3.83E-08	0	0	R14009_38	6.17	1	326.122635 pQTGG (K)	0	2	MASCOT	T
R14009_38_3,6773	K.EDK#SYGVAQR.R	1478.68699	0.00065	2	1	78.36	0	7.29E-10	0	0	R14009_38	6.17	1	326.122635 pQTGG (K)	0	2	MASCOT	T
R14009_38_3,6815	K.EDK#SYGVAQR.R	1478.68699	0.00089	2	1	50.56	0	4.40E-07	0	0	R14009_38	6.17	1	326.122635 pQTGG (K)	0	2	MASCOT	T
R14009_38_3,6850	K.EDK#SYGVAQR.R	1478.68699	0.00051	2	1	45.11	0	1.54E-06	0	0	R14009_38	6.17	1	326.122635 pQTGG (K)	0	2	MASCOT	T
R14009_38_3,4637	K.EDK@SYGVAQR.R	1495.71354	-0.00398	2	1	29.15	0	6.08E-05	0	0	R14009_38	6.17	1	343.149185 QTGG (K)	0	2	MASCOT	T
R14009_38_3,4669	K.EDK@SYGVAQR.R	1495.71354	-0.00134	2	1	34.35	0	1.84E-05	0	0	R14009_38	6.17	1	343.149185 QTGG (K)	0	2	MASCOT	T
R14009_38_3,4701	K.EDK@SYGVAQR.R	1495.71354	0.00062	2	1	34.55	0	1.75E-05	0	0	R14009_38	6.17	1	343.149185 QTGG (K)	0	2	MASCOT	T
R14009_38_3,4737	K.EDK@SYGVAQR.R	1495.71354	0.00048	2	1	48.31	0	7.38E-07	0	0	R14009_38	6.17	1	343.149185 QTGG (K)	0	2	MASCOT	T
R14009_38_3,3680	K.EDKSYGVAQR.R	1152.56436	0.00045	2	1	42.62	0	2.74E-06	0	0	R14009_38	6.17	1		0	2	MASCOT	T
R14009_38_3,3693	K.EDKSYGVAQR.R	1152.56436	0.00053	3	1	30.57	0	4.39E-05	0	0	R14009_38	6.17	1		0	2	MASCOT	T
R14009_38_3,3712	K.EDKSYGVAQR.R	1152.56436	0.00033	2	1	61.81	0	3.30E-08	0	0	R14009_38	6.17	1		0	2	MASCOT	T
R14009_38_3,3737	K.EDKSYGVAQR.R	1152.56436	0.00029	3	1	37.29	0	9.33E-06	0	0	R14009_38	6.17	1		0	2	MASCOT	T
R14009_38_3,3745	K.EDKSYGVAQR.R	1152.56436	0.00027	2	1	48.85	0	6.52E-07	0	0	R14009_38	6.17	1		0	2	MASCOT	T
R14009_38_3,3778	K.EDKSYGVAQR.R	1152.56436	0.00037	2	1	60.37	0	4.59E-08	0	0	R14009_38	6.17	1		0	2	MASCOT	T
R14009_38_3,3783	K.EDKSYGVAQR.R	1152.56436	0.00029	3	1	37.31	0	9.29E-06	0	0	R14009_38	6.17	1		0	2	MASCOT	T
R14009_38_3,3811	K.EDKSYGVAQR.R	1152.56436	0.00037	2	1	41.84	0	3.27E-06	0	0	R14009_38	6.17	1		0	2	MASCOT	T
R14009_38_3,3828	K.EDKSYGVAQR.R	1152.56436	0.00068	3	1	36.63	0	1.09E-05	0	0	R14009_38	6.17	1		0	2	MASCOT	T
R14009_38_3,3846	K.EDKSYGVAQR.R	1152.56436	-0.00023	2	1	44.43	0	1.80E-06	0	0	R14009_38	6.17	1		0	2	MASCOT	T
R14009_38_3,3891	K.EDKSYGVAQR.R	1152.56436	-0.00019	2	1	43.62	0	2.17E-06	0	0	R14009_38	6.17	1		0	2	MASCOT	T
R14009_38_3,3934	K.EDKSYGVAQR.R	1152.56436	-0.00057	2	1	26.29	0	1.17E-04	0	0	R14009_38	6.17	1		0	2	MASCOT	T
R14009_38_3,3968	K.EDKSYGVAQR.R	1152.56436	-0.00086	2	1	28.75	0	6.67E-05	0	0	R14009_38	6.17	1		0	2	MASCOT	T
R14009_38_3,9752	K.EWVSCVR.F	935.44037	0.00022	2	1	25.47	0	1.42E-04	0	0	R14009_38	6.09	0		0	2	MASCOT	T
R14009_38_3,9796	K.EWVSCVR.F	935.44037	-0.00004	2	1	29.53	0	5.57E-05	0	0	R14009_38	6.09	0		0	2	MASCOT	T
R14009_38_3,9839	K.EWVSCVR.F	935.44037	0.00048	2	1	31.64	0	3.43E-05	0	0	R14009_38	6.09	0		0	2	MASCOT	T
R14009_38_3,9880	K.EWVSCVR.F	935.44037	0.00044	2	1	33.35	0	2.31E-05	0	0	R14009_38	6.09	0		0	2	MASCOT	T
R14009_38_3,9924	K.EWVSCVR.F	935.44037	0.00016	2	1	33.44	0	2.26E-05	0	0	R14009_38	6.09	0		0	2	MASCOT	T
R14009_38_3,9969	K.EWVSCVR.F	935.44037	-0.00006	2	1	34.43	0	1.80E-05	0	0	R14009_38	6.09	0		0	2	MASCOT	T
R14009_38_3,10015	K.EWVSCVR.F	935.44037	-0.0007	2	1	33.25	0	2.37E-05	0	0	R14009_38	6.09	0		0	2	MASCOT	T
R14009_38_3,21435	K.KLYSLEAGSIIHSLCFSPNR	2292.18048	0.00068	3	1	56.47	0	1.13E-07	0	0	R14009_38	8.21	1		0	2	MASCOT	T
R14009_38_3,21478	K.KLYSLEAGSIIHSLCFSPNR	2292.18048	0.00086	3	1	83.36	0	2.31E-10	0	0	R14009_38	8.21	1		0	2	MASCOT	T
R14009_38_3,21499	K.KLYSLEAGSIIHSLCFSPNR	2292.18048	-0.00117	2	1	77.31	0	9.29E-10	0	0	R14009_38	8.21	1		0	2	MASCOT	T
R14009_38_3,21519	K.KLYSLEAGSIIHSLCFSPNR	2292.18048	0.00083	3	1	82.16	0	3.04E-10	0	0	R14009_38	8.21	1		0	2	MASCOT	T
R14009_38_3,21543	K.KLYSLEAGSIIHSLCFSPNR	2292.18048	-0.00193	2	1	77.06	0	9.84E-10	0	0	R14009_38	8.21	1		0	2	MASCOT	T
R14009_38_3,21562	K.KLYSLEAGSIIHSLCFSPNR	2292.18048	0.00275	3	1	74.42	0	1.81E-09	0	0	R14009_38	8.21	1		0	2	MASCOT	T
R14009_38_3,21607	K.KLYSLEAGSIIHSLCFSPNR	2292.18048	0.0014	3	1	56.54	0	1.11E-07	0	0	R14009_38	8.21	1		0	2	MASCOT	T
R14009_38_3,21654	K.KLYSLEAGSIIHSLCFSPNR	2292.18048	0.0029	3	1	42.74	0	2.66E-06	0	0	R14009_38	8.21	1		0	2	MASCOT	T
R14009_38_3,6896	K.LTKEDK#SYGVAQR.R	1820.91369	-0.00191	2	1	26.78	0	1.05E-04	0	0	R14009_38	8.5	2	326.122635 pQTGG (K)	0	2	MASCOT	T
R14009_38_3,5005	K.LTKEDK@SYGVAQR.R	1837.94024	0.00036	2	1	28.96	0	6.35E-05	0	0	R14009_38	8.5	2	343.149185 QTGG (K)	0	2	MASCOT	T
R14009_38_3,12610	K.LWNTLGECK.Y	1120.54555	-0.00038	2	1	41.68	0	6.79E-06	0	0	R14009_38	5.99	0		0	2	MASCOT	T
R14009_38_3,12650	K.LWNTLGECK.Y	1120.54555	-0.00024	2	1	50.02	0	9.95E-07	0	0	R14009_38	5.99	0		0	2	MASCOT	T
R14009_38_3,12681	K.LWNTLGECK.Y	1120.54555	-0.00008	2	1	50.16	0	9.64E-07	0	0	R14009_38	5.99	0		0	2	MASCOT	T
R14009_38_3,12714	K.LWNTLGECK.Y	1120.54555	-0.00068	2	1	50.12	0	9.73E-07	0	0	R14009_38	5.99	0		0	2	MASCOT	T
R14009_38_3,12747	K.LWNTLGECK.Y	1120.54555	-0.00102	2	1	50.24	0	9.46E-07	0	0	R14009_38	5.99	0		0	2	MASCOT	T
R14009_38_3,12780	K.LWNTLGECK.Y	1120.54555	-0.00106	2	1	50.24	0	9.46E-07	0	0	R14009_38	5.99	0		0	2	MASCOT	T
R14009_38_3,12813	K.LWNTLGECK.Y	1120.54555	-0.00078	2	1	50.16	0	9.64E-07	0	0	R14009_38	5.99	0		0	2	MASCOT	T
R14009_38_3,12847	K.LWNTLGECK.Y	1120.54555	-0.00024	2	1	50.11	0	9.75E-07	0	0	R14009_38	5.99	0		0	2	MASCOT	T
R14009_38_3,12882	K.LWNTLGECK.Y	1120.54555	-0.0004	2	1	45.59	0	2.76E-06	0	0	R14009_38	5.99	0		0	2	MASCOT	T
R14009_38_3,23139	K.LYSLEAGSIIHSLCFSPNR.	2164.08552	-0.00222	2	1	93.44	0	4.53E-11	0	0	R14009_38	6.74	0		0	2	MASCOT	T
R14009_38_3,23162	K.LYSLEAGSIIHSLCFSPNR.	2164.08552	0.00524	3	1	65.64	0	2.73E-08	0	0	R14009_38	6.74	0		0	2	MASCOT	T
R14009_38_3,23179	K.LYSLEAGSIIHSLCFSPNR.	2164.08552	-0.0031	2	1	105.59	0	2.76E-12	0	0	R14009_38	6.74	0		0	2	MASCOT	T
R14009_38_3,23199	K.LYSLEAGSIIHSLCFSPNR.	2164.08552	0.00056	3	1	78.34	0	1.47E-09	0	0	R14009_38	6.74	0		0	2	MASCOT	T
R14009_38_3,23221	K.LYSLEAGSIIHSLCFSPNR.	2164.08552	-0.00136	2	1	127.77	0	1.67E-14	0	0	R14009_38	6.74	0		0	2	MASCOT	T
R14009_38_3,23231	K.LYSLEAGSIIHSLCFSPNR.	2164.08552	-0.00022	3	1	82.27	0	5.93E-10	0	0	R14009_38	6.74	0		0	2	MASCOT	T
R14009_38_3,23253	K.LYSLEAGSIIHSLCFSPNR.	2164.08552	-0.00176	2	1	123.05	0	4.95E-14	0	0	R14009_38	6.74	0		0	2	MASCOT	T
R14009_38_3,23264	K.LYSLEAGSIIHSLCFSPNR.	2164.08552	-0.00031	3	1	78.1	0	1.55E-09	0	0	R14009_38	6.74	0		0	2	MASCOT	T
R14009_38_3,23286	K.LYSLEAGSIIHSLCFSPNR.	2164.08552	-0.00142	2	1	114.79	0	3.32E-13	0	0	R14009_38	6.74	0		0	2	MASCOT	T
R14009_38_3,23297	K.LYSLEAGSIIHSLCFSPNR.	2164.08552	-0.00007	3	1	74.63	0	3.44E-09	0	0	R14009_38	6.74	0		0	2	MASCOT	T
R14009_38_3,23319	K.LYSLEAGSIIHSLCFSPNR.	2164.08552	-0.00194	2	1	122.42	0	5.73E-14	0	0	R14009_38	6.74	0		0	2	MASCOT	T
R14009_38_3,23330	K.LYSLEAGSIIHSLCFSPNR.	2164.08552	-0.00036	3	1	90.31	0	9.31E-11	0	0	R14009_38	6.74	0		0	2	MASCOT	T
R14009_38_3,23351	K.LYSLEAGSIIHSLCFSPNR.	2164.08552	-0.0018	2	1	96.45	0	2.26E-11	0	0	R14009_38	6.74	0		0	2	MASCOT	T
R14009_38_3,23364	K.LYSLEAGSIIHSLCFSPNR.	2164.08552	-0.00058	3	1	64.88	0	3.25E-08	0	0	R14009_38	6.74	0		0	2	MASCOT	T
R14009_38_3,23384	K.LYSLEAGSIIHSLCFSPNR.	2164.08552	-0.00218	2	1	116.6	0	2.19E-13	0	0	R14009_38	6.74	0		0	2	MASCOT	T

R14009_38_3,23398	K.LYSLEAGSIIHSLCFSPNR.	2164.08552	-0.00106	3	1	61.34	0	7.35E-08	0	0	R14009_38	6.74	0	0	2 MASCOT	T
R14009_38_3,23417	K.LYSLEAGSIIHSLCFSPNR.	2164.08552	-0.0021	2	1	111.04	0	7.87E-13	0	0	R14009_38	6.74	0	0	2 MASCOT	T
R14009_38_3,23431	K.LYSLEAGSIIHSLCFSPNR.	2164.08552	-0.00016	3	1	66.68	0	2.15E-08	0	0	R14009_38	6.74	0	0	2 MASCOT	T
R14009_38_3,23453	K.LYSLEAGSIIHSLCFSPNR.	2164.08552	-0.00336	2	1	95.07	0	3.11E-11	0	0	R14009_38	6.74	0	0	2 MASCOT	T
R14009_38_3,23465	K.LYSLEAGSIIHSLCFSPNR.	2164.08552	-0.00081	3	1	49.06	0	1.24E-06	0	0	R14009_38	6.74	0	0	2 MASCOT	T
R14009_38_3,23488	K.LYSLEAGSIIHSLCFSPNR.	2164.08552	-0.00372	2	1	58.15	0	1.53E-07	0	0	R14009_38	6.74	0	0	2 MASCOT	T
R14009_38_3,23533	K.LYSLEAGSIIHSLCFSPNR.	2164.08552	-0.0043	2	1	34.3	0	3.72E-05	0	0	R14009_38	6.74	0	0	2 MASCOT	T
R14009_38_3,16501	K.SIILWK.L	759.47632	-0.00075	2	1	33.59	0	2.19E-05	0	0	R14009_38	8.47	0	0	2 MASCOT	T
R14009_38_3,16531	K.SIILWK.L	759.47632	-0.00123	2	1	33.68	0	2.14E-05	0	0	R14009_38	8.47	0	0	2 MASCOT	T
R14009_38_3,16564	K.SIILWK.L	759.47632	-0.00043	2	1	33.91	0	2.03E-05	0	0	R14009_38	8.47	0	0	2 MASCOT	T
R14009_38_3,16597	K.SIILWK.L	759.47632	-0.00033	2	1	33.94	0	2.02E-05	0	0	R14009_38	8.47	0	0	2 MASCOT	T
R14009_38_3,16630	K.SIILWK.L	759.47632	-0.00101	2	1	33.88	0	2.05E-05	0	0	R14009_38	8.47	0	0	2 MASCOT	T
R14009_38_3,16663	K.SIILWK.L	759.47632	-0.00173	2	1	33.76	0	2.10E-05	0	0	R14009_38	8.47	0	0	2 MASCOT	T
R14009_38_3,16696	K.SIILWK.L	759.47632	-0.00103	2	1	33.68	0	2.14E-05	0	0	R14009_38	8.47	0	0	2 MASCOT	T
R14009_38_3,16729	K.SIILWK.L	759.47632	-0.00109	2	1	33.34	0	2.32E-05	0	0	R14009_38	8.47	0	0	2 MASCOT	T
R14009_38_3,16762	K.SIILWK.L	759.47632	-0.00049	2	1	32.84	0	2.60E-05	0	0	R14009_38	8.47	0	0	2 MASCOT	T
R14009_38_3,7673	K.SVVEDLK.V	789.43526	-0.00067	2	1	34.18	0	1.91E-05	0	0	R14009_38	4.37	0	0	2 MASCOT	T
R14009_38_3,7717	K.SVVEDLK.V	789.43526	-0.00011	2	1	36.75	0	1.06E-05	0	0	R14009_38	4.37	0	0	2 MASCOT	T
R14009_38_3,7747	K.SVVEDLK.V	789.43526	-0.00019	2	1	36.86	0	1.03E-05	0	0	R14009_38	4.37	0	0	2 MASCOT	T
R14009_38_3,7780	K.SVVEDLK.V	789.43526	-0.00035	2	1	35.5	0	1.41E-05	0	0	R14009_38	4.37	0	0	2 MASCOT	T
R14009_38_3,7813	K.SVVEDLK.V	789.43526	-0.00079	2	1	35.58	0	1.38E-05	0	0	R14009_38	4.37	0	0	2 MASCOT	T
R14009_38_3,7846	K.SVVEDLK.V	789.43526	-0.00069	2	1	36.91	0	1.02E-05	0	0	R14009_38	4.37	0	0	2 MASCOT	T
R14009_38_3,7879	K.SVVEDLK.V	789.43526	-0.00085	2	1	35.51	0	1.41E-05	0	0	R14009_38	4.37	0	0	2 MASCOT	T
R14009_38_3,7912	K.SVVEDLK.V	789.43526	-0.00051	2	1	36.87	0	1.03E-05	0	0	R14009_38	4.37	0	0	2 MASCOT	T
R14009_38_3,7945	K.SVVEDLK.V	789.43526	-0.00003	2	1	35.5	0	1.41E-05	0	0	R14009_38	4.37	0	0	2 MASCOT	T
R14009_38_3,7979	K.SVVEDLK.V	789.43526	0.00017	2	1	36.91	0	1.02E-05	0	0	R14009_38	4.37	0	0	2 MASCOT	T
R14009_38_3,8012	K.SVVEDLK.V	789.43526	0.00047	2	1	35.86	0	1.30E-05	0	0	R14009_38	4.37	0	0	2 MASCOT	T
R14009_38_3,18497	K.SVVEDLK@VDLKAEAEK.T	2116.11317	-0.00597	2	1	22.68	0	5.40E-04	0	0	R14009_38	4.51	2 343.149185 QTGG (K)	0	2 MASCOT	T
R14009_38_3,18541	K.SVVEDLK@VDLKAEAEK.T	2116.11317	-0.00509	2	1	34.74	0	3.36E-05	0	0	R14009_38	4.51	2 343.149185 QTGG (K)	0	2 MASCOT	T
R14009_38_3,15392	K.SVVEDLKVDLK.A	1244.70964	-0.00085	2	1	61.7	0	3.38E-08	0	0	R14009_38	4.56	1	0	2 MASCOT	T
R14009_38_3,15426	K.SVVEDLKVDLK.A	1244.70964	0.00054	3	1	33.34	0	2.32E-05	0	0	R14009_38	4.56	1	0	2 MASCOT	T
R14009_38_3,15468	K.SVVEDLKVDLK.A	1244.70964	0.00021	3	1	46.71	0	1.07E-06	0	0	R14009_38	4.56	1	0	2 MASCOT	T
R14009_38_3,15477	K.SVVEDLKVDLK.A	1244.70964	-0.00345	2	1	62.79	0	2.63E-08	0	0	R14009_38	4.56	1	0	2 MASCOT	T
R14009_38_3,15508	K.SVVEDLKVDLK.A	1244.70964	-0.00012	3	1	46.87	0	1.03E-06	0	0	R14009_38	4.56	1	0	2 MASCOT	T
R14009_38_3,15541	K.SVVEDLKVDLK.A	1244.70964	0.00003	3	1	49.12	0	6.12E-07	0	0	R14009_38	4.56	1	0	2 MASCOT	T
R14009_38_3,15574	K.SVVEDLKVDLK.A	1244.70964	-0.00006	3	1	45.49	0	1.41E-06	0	0	R14009_38	4.56	1	0	2 MASCOT	T
R14009_38_3,15609	K.SVVEDLKVDLK.A	1244.70964	-0.00033	3	1	49.24	0	5.96E-07	0	0	R14009_38	4.56	1	0	2 MASCOT	T
R14009_38_3,15642	K.SVVEDLKVDLK.A	1244.70964	0.00039	3	1	47.76	0	8.37E-07	0	0	R14009_38	4.56	1	0	2 MASCOT	T
R14009_38_3,15676	K.SVVEDLKVDLK.A	1244.70964	0.00012	3	1	47.81	0	8.28E-07	0	0	R14009_38	4.56	1	0	2 MASCOT	T
R14009_38_3,15712	K.SVVEDLKVDLK.A	1244.70964	0.00018	3	1	44.02	0	1.98E-06	0	0	R14009_38	4.56	1	0	2 MASCOT	T
R14009_38_3,15759	K.SVVEDLKVDLK.A	1244.70964	0.00057	3	1	45.88	0	1.29E-06	0	0	R14009_38	4.56	1	0	2 MASCOT	T
R14009_38_3,18121	K.SVVEDLKVDLK@AEAEK.T	2116.11317	-0.00793	2	1	36.5	0	2.24E-05	0	0	R14009_38	4.51	2 343.149185 QTGG (K)	0	2 MASCOT	T
R14009_38_3,18133	K.SVVEDLKVDLK@AEAEK.T	2116.11317	-0.00266	3	1	22.08	0	6.19E-04	0	0	R14009_38	4.51	2 343.149185 QTGG (K)	0	2 MASCOT	T
R14009_38_3,18179	K.SVVEDLKVDLK@AEAEK.T	2116.11317	-0.00669	2	1	47.58	0	1.75E-06	0	0	R14009_38	4.51	2 343.149185 QTGG (K)	0	2 MASCOT	T
R14009_38_3,18267	K.SVVEDLKVDLK@AEAEK.T	2116.11317	-0.00493	2	1	30.68	0	8.55E-05	0	0	R14009_38	4.51	2 343.149185 QTGG (K)	0	2 MASCOT	T
R14009_38_3,18311	K.SVVEDLKVDLK@AEAEK.T	2116.11317	-0.00551	2	1	45.33	0	2.93E-06	0	0	R14009_38	4.51	2 343.149185 QTGG (K)	0	2 MASCOT	T
R14009_38_3,18364	K.SVVEDLKVDLK@AEAEK.T	2116.11317	-0.00623	2	1	45.45	0	2.85E-06	0	0	R14009_38	4.51	2 343.149185 QTGG (K)	0	2 MASCOT	T
R14009_38_3,18409	K.SVVEDLKVDLK@AEAEK.T	2116.11317	-0.00579	2	1	34.38	0	3.65E-05	0	0	R14009_38	4.51	2 343.149185 QTGG (K)	0	2 MASCOT	T
R14009_38_3,3967	K.SYGVAQR.R	780.39987	-0.00019	2	1	28.91	0	6.43E-05	0	0	R14009_38	8.46	0	0	2 MASCOT	T
R14009_38_3,3997	K.SYGVAQR.R	780.39987	-0.00032	2	1	29.44	0	5.69E-05	0	0	R14009_38	8.46	0	0	2 MASCOT	T
R14009_38_3,4030	K.SYGVAQR.R	780.39987	-0.00033	2	1	28.47	0	7.11E-05	0	0	R14009_38	8.46	0	0	2 MASCOT	T
R14009_38_3,4063	K.SYGVAQR.R	780.39987	-0.00027	2	1	29.12	0	6.12E-05	0	0	R14009_38	8.46	0	0	2 MASCOT	T
R14009_38_3,4096	K.SYGVAQR.R	780.39987	-0.00045	2	1	31.67	0	3.40E-05	0	0	R14009_38	8.46	0	0	2 MASCOT	T
R14009_38_3,4129	K.SYGVAQR.R	780.39987	-0.00047	2	1	28.56	0	6.97E-05	0	0	R14009_38	8.46	0	0	2 MASCOT	T
R14009_38_3,4162	K.SYGVAQR.R	780.39987	-0.00015	2	1	28.62	0	6.87E-05	0	0	R14009_38	8.46	0	0	2 MASCOT	T
R14009_38_3,4195	K.SYGVAQR.R	780.39987	-0.00032	2	1	28.49	0	7.08E-05	0	0	R14009_38	8.46	0	0	2 MASCOT	T
R14009_38_3,4228	K.SYGVAQR.R	780.39987	0.00009	2	1	28.13	0	7.69E-05	0	0	R14009_38	8.46	0	0	2 MASCOT	T
R14009_38_3,4263	K.SYGVAQR.R	780.39987	0.00003	2	1	27.44	0	9.02E-05	0	0	R14009_38	8.46	0	0	2 MASCOT	T
R14009_38_3,4966	K.TDGSTGIGNK#TK.V	1504.7238	0.00037	2	1	73.26	0	2.36E-09	0	0	R14009_38	8.26	1 326.122635 pQTGG (K)	0	2 MASCOT	T
R14009_38_3,4998	K.TDGSTGIGNK#TK.V	1504.7238	0.00025	2	1	61.69	0	3.39E-08	0	0	R14009_38	8.26	1 326.122635 pQTGG (K)	0	2 MASCOT	T
R14009_38_3,5064	K.TDGSTGIGNK#TK.V	1504.7238	-0.00055	2	1	61.74	0	3.35E-08	0	0	R14009_38	8.26	1 326.122635 pQTGG (K)	0	2 MASCOT	T
R14009_38_3,5099	K.TDGSTGIGNK#TK.V	1504.7238	-0.00087	2	1	45.18	0	1.52E-06	0	0	R14009_38	8.26	1 326.122635 pQTGG (K)	0	2 MASCOT	T
R14009_38_3,3075	K.TDGSTGIGNK@TK.V	1521.75035	0.00113	2	1	25.93	0	1.28E-04	0	0	R14009_38	8.26	1 343.149185 QTGG (K)	0	2 MASCOT	T
R14009_38_3,4881	K.TDGSTGIGNKTK#.V ! K.	1504.7238	0.00067	2	1	53.95	0	2.01E-07	0	0	R14009_38 ! R14009_38	8.26	1 326.122635 pQTGG (K)	0	2 MASCOT	T
R14009_38_3,4923	K.TDGSTGIGNKTK#.V ! K.	1504.7238	0.00065	2	1	65.37	0	1.45E-08	0	0	R14009_38 ! R14009_38	8.26	1 326.122635 pQTGG (K)	0	2 MASCOT	T
R14009_38_3,5030	K.TDGSTGIGNKTK#.V ! K.	1504.7238	0.00073	2	1	64.75	0	1.67E-08	0	0	R14009_38 ! R14009_38	8.26	1 326.122635 pQTGG (K)	0	2 MASCOT	T
R14009_38_3,5133	K.TDGSTGIGNKTK#.V ! K.	1504.7238	-0.00039	2	1	41.79	0	3.31E-06	0	0	R14009_38 ! R14009_38	8.26	1 326.122635 pQTGG (K)	0	2 MASCOT	T
R14009_38_3,8820	K.VDLK#AEAEK.T	1328.6692	0.0016	2	1	61.36	0	3.66E-08	0	0	R14009_38	4.68	1 326.122635 pQTGG (K)	0	2 MASCOT	T
R14009_38_3,8861	K.VDLK#AEAEK.T	1328.6692	0.00084	2	1	62.87	0	2.58E-08	0	0	R14009_38	4.68	1 326.122635 pQTGG (K)	0	2 MASCOT	T
R14009_38_3,8891	K.VDLK#AEAEK.T	1328.6692	0.00146	2	1	62.75	0	2.65E-08	0	0	R14009_38	4.68	1 326.122635 pQTGG (K)	0	2 MASCOT	T
R14009_38_3,8924	K.VDLK#AEAEK.T	1328.6692	0.00146	2	1	58.56	0	6.97E-08	0	0	R14009_38	4.68	1 326.122635 pQTGG (K)	0	2 MASCOT	T
R14009_38_3,8957	K.VDLK#AEAEK.T	1328.6692	0.0011	2	1	58.39	0	7.24E-08	0	0	R14009_38	4.68	1 326.122635 pQTGG (K)	0	2 MASCOT	T
R14009_38_3,8990	K.VDLK#AEAEK.T	1328.6692	0.00074	2	1	62.48	0	2.82E-08	0	0	R14009_38	4.68	1 326.122635 pQTGG (K)	0	2 MASCOT	T

R14009_38_3,9023	K.VDLK#AEAEK.T	1328.6692	0.00072	2	1	58.47	0	7.11E-08	0	0	R14009_38	4.68	1	326.122635 pQTGG (K)	0	2	MASCOT	T
R14009_38_3,9056	K.VDLK#AEAEK.T	1328.6692	0.00008	2	1	54.52	0	1.77E-07	0	0	R14009_38	4.68	1	326.122635 pQTGG (K)	0	2	MASCOT	T
R14009_38_3,9089	K.VDLK#AEAEK.T	1328.6692	-0.00004	2	1	54.8	0	1.66E-07	0	0	R14009_38	4.68	1	326.122635 pQTGG (K)	0	2	MASCOT	T
R14009_38_3,9122	K.VDLK#AEAEK.T	1328.6692	0.00022	2	1	59.03	0	6.25E-08	0	0	R14009_38	4.68	1	326.122635 pQTGG (K)	0	2	MASCOT	T
R14009_38_3,9161	K.VDLK#AEAEK.T	1328.6692	0.0002	2	1	50.77	0	4.19E-07	0	0	R14009_38	4.68	1	326.122635 pQTGG (K)	0	2	MASCOT	T
R14009_38_3,12105	K.VDLK#AEAEKTDGSTGIGNK	2259.10989	-0.00133	3	1	65.88	0	1.29E-08	0	0	R14009_38	4.78	2	326.122635 pQTGG (K)	0	2	MASCOT	T
R14009_38_3,12116	K.VDLK#AEAEKTDGSTGIGNK	2259.10989	-0.00013	2	1	71.13	0	3.85E-09	0	0	R14009_38	4.78	2	326.122635 pQTGG (K)	0	2	MASCOT	T
R14009_38_3,12147	K.VDLK#AEAEKTDGSTGIGNK	2259.10989	-0.00007	3	1	73.31	0	2.33E-09	0	0	R14009_38	4.78	2	326.122635 pQTGG (K)	0	2	MASCOT	T
R14009_38_3,12158	K.VDLK#AEAEKTDGSTGIGNK	2259.10989	-0.00167	2	1	82.31	0	2.94E-10	0	0	R14009_38	4.78	2	326.122635 pQTGG (K)	0	2	MASCOT	T
R14009_38_3,12191	K.VDLK#AEAEKTDGSTGIGNK	2259.10989	-0.00178	3	1	73.78	0	2.09E-09	0	0	R14009_38	4.78	2	326.122635 pQTGG (K)	0	2	MASCOT	T
R14009_38_3,6533	K.VDLK@AEAEK.T	1345.69575	-0.00031	2	1	26.91	0	1.02E-04	0	0	R14009_38	4.68	1	343.149185 QTGG (K)	0	2	MASCOT	T
R14009_38_3,6570	K.VDLK@AEAEK.T	1345.69575	0.00021	2	1	23.21	0	2.39E-04	0	0	R14009_38	4.68	1	343.149185 QTGG (K)	0	2	MASCOT	T
R14009_38_3,6603	K.VDLK@AEAEK.T	1345.69575	0.00025	2	1	20.84	0	4.12E-04	0	0	R14009_38	4.68	1	343.149185 QTGG (K)	0	2	MASCOT	T
R14009_38_3,6625	K.VDLK@AEAEK.T	1345.69575	0.00011	3	1	20.11	0	4.87E-04	0	0	R14009_38	4.68	1	343.149185 QTGG (K)	0	2	MASCOT	T
R14009_38_3,6636	K.VDLK@AEAEK.T	1345.69575	0.00021	2	1	23.36	0	2.31E-04	0	0	R14009_38	4.68	1	343.149185 QTGG (K)	0	2	MASCOT	T
R14009_38_3,6669	K.VDLK@AEAEK.T	1345.69575	-0.00007	2	1	20.9	0	4.06E-04	0	0	R14009_38	4.68	1	343.149185 QTGG (K)	0	2	MASCOT	T
R14009_38_3,6702	K.VDLK@AEAEK.T	1345.69575	-0.00029	2	1	23.33	0	2.32E-04	0	0	R14009_38	4.68	1	343.149185 QTGG (K)	0	2	MASCOT	T
R14009_38_3,6735	K.VDLK@AEAEK.T	1345.69575	0.00111	2	1	23.27	0	2.35E-04	0	0	R14009_38	4.68	1	343.149185 QTGG (K)	0	2	MASCOT	T
R14009_38_3,6769	K.VDLK@AEAEK.T	1345.69575	0.00051	2	1	23.31	0	2.33E-04	0	0	R14009_38	4.68	1	343.149185 QTGG (K)	0	2	MASCOT	T
R14009_38_3,6802	K.VDLK@AEAEK.T	1345.69575	0.00077	2	1	23.23	0	2.38E-04	0	0	R14009_38	4.68	1	343.149185 QTGG (K)	0	2	MASCOT	T
R14009_38_3,6838	K.VDLK@AEAEK.T	1345.69575	0.00061	2	1	21.44	0	3.59E-04	0	0	R14009_38	4.68	1	343.149185 QTGG (K)	0	2	MASCOT	T
R14009_38_3,10324	K.VDLK@AEAEKTDGSTGIGNK	2276.13644	-0.00214	2	1	33.13	0	2.43E-05	0	0	R14009_38	4.78	2	343.149185 QTGG (K)	0	2	MASCOT	T
R14009_38_3,10330	K.VDLK@AEAEKTDGSTGIGNK	2276.13644	0.00103	3	1	82.45	0	2.84E-10	0	0	R14009_38	4.78	2	343.149185 QTGG (K)	0	2	MASCOT	T
R14009_38_3,10336	K.VDLK@AEAEKTDGSTGIGNK	2276.13644	-0.00081	4	1	37.68	0	8.53E-06	0	0	R14009_38	4.78	2	343.149185 QTGG (K)	0	2	MASCOT	T
R14009_38_3,10368	K.VDLK@AEAEKTDGSTGIGNK	2276.13644	-0.00146	2	1	72.49	0	2.82E-09	0	0	R14009_38	4.78	2	343.149185 QTGG (K)	0	2	MASCOT	T
R14009_38_3,10375	K.VDLK@AEAEKTDGSTGIGNK	2276.13644	0.00271	3	1	82.77	0	2.64E-10	0	0	R14009_38	4.78	2	343.149185 QTGG (K)	0	2	MASCOT	T
R14009_38_3,10377	K.VDLK@AEAEKTDGSTGIGNK	2276.13644	0.00143	4	1	59.57	0	5.52E-08	0	0	R14009_38	4.78	2	343.149185 QTGG (K)	0	2	MASCOT	T
R14009_38_3,10409	K.VDLK@AEAEKTDGSTGIGNK	2276.13644	-0.00132	2	1	89.63	0	5.44E-11	0	0	R14009_38	4.78	2	343.149185 QTGG (K)	0	2	MASCOT	T
R14009_38_3,10417	K.VDLK@AEAEKTDGSTGIGNK	2276.13644	0.00139	3	1	91.43	0	3.60E-11	0	0	R14009_38	4.78	2	343.149185 QTGG (K)	0	2	MASCOT	T
R14009_38_3,10419	K.VDLK@AEAEKTDGSTGIGNK	2276.13644	-0.00013	4	1	50.59	0	4.36E-07	0	0	R14009_38	4.78	2	343.149185 QTGG (K)	0	2	MASCOT	T
R14009_38_3,10451	K.VDLK@AEAEKTDGSTGIGNK	2276.13644	0.00178	3	1	86.09	0	1.23E-10	0	0	R14009_38	4.78	2	343.149185 QTGG (K)	0	2	MASCOT	T
R14009_38_3,10452	K.VDLK@AEAEKTDGSTGIGNK	2276.13644	-0.00068	2	1	81.84	0	3.27E-10	0	0	R14009_38	4.78	2	343.149185 QTGG (K)	0	2	MASCOT	T
R14009_38_3,10465	K.VDLK@AEAEKTDGSTGIGNK	2276.13644	-0.00065	4	1	49.72	0	5.33E-07	0	0	R14009_38	4.78	2	343.149185 QTGG (K)	0	2	MASCOT	T
R14009_38_3,10496	K.VDLK@AEAEKTDGSTGIGNK	2276.13644	-0.00146	3	1	63.11	0	2.44E-08	0	0	R14009_38	4.78	2	343.149185 QTGG (K)	0	2	MASCOT	T
R14009_38_3,5400	K.VDLKAEAEK.T	1002.54657	-0.00026	2	1	56.51	0	1.12E-07	0	0	R14009_38	4.68	1		0	2	MASCOT	T
R14009_38_3,5439	K.VDLKAEAEK.T	1002.54657	-0.00062	2	1	53.65	0	2.16E-07	0	0	R14009_38	4.68	1		0	2	MASCOT	T
R14009_38_3,5472	K.VDLKAEAEK.T	1002.54657	-0.00048	2	1	54.81	0	1.65E-07	0	0	R14009_38	4.68	1		0	2	MASCOT	T
R14009_38_3,10795	K.VWNLQNCK.L	1061.51967	-0.00108	2	1	24.46	0	1.79E-04	0	0	R14009_38	8.19	0		0	2	MASCOT	T
R14009_38_3,10835	K.VWNLQNCK.L	1061.51967	-0.00088	2	1	32.05	0	3.12E-05	0	0	R14009_38	8.19	0		0	2	MASCOT	T
R14009_38_3,10867	K.VWNLQNCK.L	1061.51967	-0.00042	2	1	42.57	0	2.77E-06	0	0	R14009_38	8.19	0		0	2	MASCOT	T
R14009_38_3,10900	K.VWNLQNCK.L	1061.51967	-0.00002	2	1	45.69	0	1.35E-06	0	0	R14009_38	8.19	0		0	2	MASCOT	T
R14009_38_3,10933	K.VWNLQNCK.L	1061.51967	0.00006	2	1	42.77	0	2.64E-06	0	0	R14009_38	8.19	0		0	2	MASCOT	T
R14009_38_3,10966	K.VWNLQNCK.L	1061.51967	0.00048	2	1	46.04	0	1.24E-06	0	0	R14009_38	8.19	0		0	2	MASCOT	T
R14009_38_3,11000	K.VWNLQNCK.L	1061.51967	0.0003	2	1	45.99	0	1.26E-06	0	0	R14009_38	8.19	0		0	2	MASCOT	T
R14009_38_3,11033	K.VWNLQNCK.L	1061.51967	0.0003	2	1	46.08	0	1.23E-06	0	0	R14009_38	8.19	0		0	2	MASCOT	T
R14009_38_3,11065	K.VWNLQNCK.L	1061.51967	0.00036	2	1	45.97	0	1.26E-06	0	0	R14009_38	8.19	0		0	2	MASCOT	T
R14009_38_3,11099	K.VWNLQNCK.L	1061.51967	-0.00042	2	1	49.13	0	6.11E-07	0	0	R14009_38	8.19	0		0	2	MASCOT	T
R14009_38_3,5168	K.YTISEADGHK.E	1120.52691	-0.00019	2	1	61.19	0	7.60E-08	0	0	R14009_38	5.32	0		0	2	MASCOT	T
R14009_38_3,5208	K.YTISEADGHK.E	1120.52691	-0.00025	2	1	61.04	0	7.87E-08	0	0	R14009_38	5.32	0		0	2	MASCOT	T
R14009_38_3,5241	K.YTISEADGHK.E	1120.52691	-0.00071	2	1	40.23	0	9.48E-06	0	0	R14009_38	5.32	0		0	2	MASCOT	T
R14009_38_3,5273	K.YTISEADGHK.E	1120.52691	-0.00031	2	1	29.16	0	1.21E-04	0	0	R14009_38	5.32	0		0	2	MASCOT	T
R14009_38_3,5714	K.YTISEADGHK.E	1120.52691	0.00011	2	1	35.02	0	3.15E-05	0	0	R14009_38	5.32	0		0	2	MASCOT	T
R14009_38_3,5745	K.YTISEADGHK.E	1120.52691	-0.00031	2	1	61.34	0	7.35E-08	0	0	R14009_38	5.32	0		0	2	MASCOT	T
R14009_38_3,5769	K.YTISEADGHK.E	1120.52691	-0.00036	3	1	46.61	0	2.18E-06	0	0	R14009_38	5.32	0		0	2	MASCOT	T
R14009_38_3,5778	K.YTISEADGHK.E	1120.52691	-0.00083	2	1	61.68	0	6.79E-08	0	0	R14009_38	5.32	0		0	2	MASCOT	T
R14009_38_3,5800	K.YTISEADGHK.E	1120.52691	-0.00048	3	1	46.83	0	2.07E-06	0	0	R14009_38	5.32	0		0	2	MASCOT	T
R14009_38_3,5811	K.YTISEADGHK.E	1120.52691	-0.00195	2	1	61.77	0	6.65E-08	0	0	R14009_38	5.32	0		0	2	MASCOT	T
R14009_38_3,5833	K.YTISEADGHK.E	1120.52691	-0.00066	3	1	43.21	0	4.78E-06	0	0	R14009_38	5.32	0		0	2	MASCOT	T
R14009_38_3,5844	K.YTISEADGHK.E	1120.52691	-0.00209	2	1	61.78	0	6.64E-08	0	0	R14009_38	5.32	0		0	2	MASCOT	T
R14009_38_3,5866	K.YTISEADGHK.E	1120.52691	-0.00039	3	1	46.88	0	2.05E-06	0	0	R14009_38	5.32	0		0	2	MASCOT	T
R14009_38_3,5877	K.YTISEADGHK.E	1120.52691	-0.00247	2	1	61.75	0	6.68E-08	0	0	R14009_38	5.32	0		0	2	MASCOT	T
R14009_38_3,5899	K.YTISEADGHK.E	1120.52691	0.00006	3	1	46.65	0	2.16E-06	0	0	R14009_38	5.32	0		0	2	MASCOT	T
R14009_38_3,5910	K.YTISEADGHK.E	1120.52691	-0.00197	2	1	57.66	0	1.71E-07	0	0	R14009_38	5.32	0		0	2	MASCOT	T
R14009_38_3,5932	K.YTISEADGHK.E	1120.52691	-0.00003	3	1	46.68	0	2.15E-06	0	0	R14009_38	5.32	0		0	2	MASCOT	T
R14009_38_3,5943	K.YTISEADGHK.E	1120.52691	-0.00101	2	1	61.7	0	6.76E-08	0	0	R14009_38	5.32	0		0	2	MASCOT	T
R14009_38_3,5966	K.YTISEADGHK.E	1120.52691	-0.00042	3	1	27.04	0	1.98E-04	0	0	R14009_38	5.32	0		0	2	MASCOT	T
R14009_38_3,5979	K.YTISEADGHK.E	1120.52691	-0.00067	2	1	61.62	0	6.89E-08	0	0	R14009_38	5.32	0		0	2	MASCOT	T
R14009_38_3,6012	K.YTISEADGHK.E	1120.52691	-0.00085	2	1	61.61	0	6.90E-08	0	0	R14009_38	5.32	0		0	2	MASCOT	T
R14009_38_3,12502	K.YTISEADGHKEWVSCVR.F	2036.94945	-0.00154	3	1	47.55	0	8.79E-07	0	0	R14009_38	5.45	1		0	2	MASCOT	T
R14009_38_3,12514	K.YTISEADGHKEWVSCVR.F	2036.94945	-0.00002	2	1	110.02	0	4.98E-13	0	0	R14009_38	5.45	1		0	2	MASCOT	T
R14009_38_3,12540	K.YTISEADGHKEWVSCVR.F	2036.94945	-0.00064	3	1	66.79	0	1.05E-08	0	0	R14009_38	5.45	1		0	2	MASCOT	T
R14009_38_3,12550	K.YTISEADGHKEWVSCVR.F	2036.94945	-0.00142	2	1	104.22	0	1.89E-12	0	0	R14009_38	5.45	1		0	2	MASCOT	T
R14009_38_3,12552	K.YTISEADGHKEWVSCVR.F	2036.94945	-0.00189	4	1	29.11	0	6.14E-05	0	0	R14009_38	5.45	1		0	2	MASCOT	T

R14009_38_3,12572	K.YTISEADGHKEWVSCVR.F	2036.94945	-0.00088	3	1	55.85	0	1.30E-07	0	0	R14009_38	5.45	1	0	2	MASCOT	T
R14009_38_3,12583	K.YTISEADGHKEWVSCVR.F	2036.94945	-0.00145	4	1	34.58	0	1.74E-05	0	0	R14009_38	5.45	1	0	2	MASCOT	T
R14009_38_3,12584	K.YTISEADGHKEWVSCVR.F	2036.94945	-0.00288	2	1	143.99	0	2.00E-16	0	0	R14009_38	5.45	1	0	2	MASCOT	T
R14009_38_3,12605	K.YTISEADGHKEWVSCVR.F	2036.94945	0.00002	3	1	55.78	0	1.32E-07	0	0	R14009_38	5.45	1	0	2	MASCOT	T
R14009_38_3,12615	K.YTISEADGHKEWVSCVR.F	2036.94945	-0.00198	2	1	108.5	0	7.06E-13	0	0	R14009_38	5.45	1	0	2	MASCOT	T
R14009_38_3,12617	K.YTISEADGHKEWVSCVR.F	2036.94945	-0.00141	4	1	37.84	0	8.22E-06	0	0	R14009_38	5.45	1	0	2	MASCOT	T
R14009_38_3,12638	K.YTISEADGHKEWVSCVR.F	2036.94945	-0.00037	3	1	78.71	0	6.73E-10	0	0	R14009_38	5.45	1	0	2	MASCOT	T
R14009_38_3,12648	K.YTISEADGHKEWVSCVR.F	2036.94945	-0.00186	2	1	150.81	0	4.15E-17	0	0	R14009_38	5.45	1	0	2	MASCOT	T
R14009_38_3,12649	K.YTISEADGHKEWVSCVR.F	2036.94945	-0.00081	4	1	36.38	0	1.15E-05	0	0	R14009_38	5.45	1	0	2	MASCOT	T
R14009_38_3,12671	K.YTISEADGHKEWVSCVR.F	2036.94945	-0.0004	3	1	67.1	0	9.75E-09	0	0	R14009_38	5.45	1	0	2	MASCOT	T
R14009_38_3,12682	K.YTISEADGHKEWVSCVR.F	2036.94945	-0.00198	2	1	116.76	0	1.05E-13	0	0	R14009_38	5.45	1	0	2	MASCOT	T
R14009_38_3,12683	K.YTISEADGHKEWVSCVR.F	2036.94945	-0.00161	4	1	31.81	0	3.30E-05	0	0	R14009_38	5.45	1	0	2	MASCOT	T
R14009_38_3,12704	K.YTISEADGHKEWVSCVR.F	2036.94945	-0.00019	3	1	70.71	0	4.25E-09	0	0	R14009_38	5.45	1	0	2	MASCOT	T
R14009_38_3,12716	K.YTISEADGHKEWVSCVR.F	2036.94945	-0.00232	2	1	115.81	0	1.31E-13	0	0	R14009_38	5.45	1	0	2	MASCOT	T
R14009_38_3,12717	K.YTISEADGHKEWVSCVR.F	2036.94945	-0.00041	4	1	37.46	0	8.97E-06	0	0	R14009_38	5.45	1	0	2	MASCOT	T
R14009_38_3,12739	K.YTISEADGHKEWVSCVR.F	2036.94945	-0.00019	3	1	49.69	0	5.37E-07	0	0	R14009_38	5.45	1	0	2	MASCOT	T
R14009_38_3,12752	K.YTISEADGHKEWVSCVR.F	2036.94945	-0.00372	2	1	103.76	0	2.10E-12	0	0	R14009_38	5.45	1	0	2	MASCOT	T
R14009_38_3,12753	K.YTISEADGHKEWVSCVR.F	2036.94945	-0.00145	4	1	26.33	0	1.16E-04	0	0	R14009_38	5.45	1	0	2	MASCOT	T
R14009_38_3,12772	K.YTISEADGHKEWVSCVR.F	2036.94945	-0.00061	3	1	53.58	0	2.19E-07	0	0	R14009_38	5.45	1	0	2	MASCOT	T
R14009_38_3,12797	K.YTISEADGHKEWVSCVR.F	2036.94945	-0.00278	2	1	60.49	0	4.47E-08	0	0	R14009_38	5.45	1	0	2	MASCOT	T
R14009_38_3,12807	K.YTISEADGHKEWVSCVR.F	2036.94945	-0.00088	3	1	47.1	0	9.75E-07	0	0	R14009_38	5.45	1	0	2	MASCOT	T
R14009_38_3,12842	K.YTISEADGHKEWVSCVR.F	2036.94945	-0.0016	2	1	53.01	0	2.50E-07	0	0	R14009_38	5.45	1	0	2	MASCOT	T
R14009_38_3,12851	K.YTISEADGHKEWVSCVR.F	2036.94945	-0.00061	3	1	48.13	0	7.69E-07	0	0	R14009_38	5.45	1	0	2	MASCOT	T
R14009_38_3,12886	K.YTISEADGHKEWVSCVR.F	2036.94945	-0.00082	2	1	50.42	0	4.54E-07	0	0	R14009_38	5.45	1	0	2	MASCOT	T
R14009_38_3,12894	K.YTISEADGHKEWVSCVR.F	2036.94945	-0.00067	3	1	28.62	0	6.87E-05	0	0	R14009_38	5.45	1	0	2	MASCOT	T
R14009_38_3,13031	K.YTISEADGHKEWVSCVR.F	2036.94945	-0.0034	3	1	34.87	0	1.63E-05	0	0	R14009_38	5.45	1	0	2	MASCOT	T
R14009_38_3,13097	K.YTISEADGHKEWVSCVR.F	2036.94945	-0.00628	3	1	45.2	0	1.51E-06	0	0	R14009_38	5.45	1	0	2	MASCOT	T
R14009_38_3,8655	M.AEGLVLK.G	729.4505	0.00023	2	1	40.1	0	4.89E-06	0	0	R14009_38	6.05	0	0	2	MASCOT	T
R14009_38_3,8710	M.AEGLVLK.G	729.4505	0.00011	2	1	23.94	0	2.02E-04	0	0	R14009_38	6.05	0	0	2	MASCOT	T
R14009_38_3,8756	M.AEGLVLK.G	729.4505	-0.00039	2	1	32.39	0	2.88E-05	0	0	R14009_38	6.05	0	0	2	MASCOT	T
R14009_38_3,8801	M.AEGLVLK.G	729.4505	-0.00013	2	1	25	0	1.58E-04	0	0	R14009_38	6.05	0	0	2	MASCOT	T
R14009_38_3,14532	R.DKSIILWK.L	1002.59822	-0.00065	2	1	29.36	0	5.79E-05	0	0	R14009_38	8.59	1	0	2	MASCOT	T
R14009_38_3,14562	R.DKSIILWK.L	1002.59822	-0.00053	2	1	43.69	0	2.14E-06	0	0	R14009_38	8.59	1	0	2	MASCOT	T
R14009_38_3,14592	R.DKSIILWK.L	1002.59822	0.00091	3	1	25.22	0	1.50E-04	0	0	R14009_38	8.59	1	0	2	MASCOT	T
R14009_38_3,14595	R.DKSIILWK.L	1002.59822	-0.00171	2	1	38.28	0	7.43E-06	0	0	R14009_38	8.59	1	0	2	MASCOT	T
R14009_38_3,14628	R.DKSIILWK.L	1002.59822	-0.00191	2	1	37.98	0	7.96E-06	0	0	R14009_38	8.59	1	0	2	MASCOT	T
R14009_38_3,14642	R.DKSIILWK.L	1002.59822	0.00082	3	1	30.88	0	4.08E-05	0	0	R14009_38	8.59	1	0	2	MASCOT	T
R14009_38_3,14661	R.DKSIILWK.L	1002.59822	-0.00305	2	1	39.51	0	5.60E-06	0	0	R14009_38	8.59	1	0	2	MASCOT	T
R14009_38_3,14675	R.DKSIILWK.L	1002.59822	0.0007	3	1	26	0	1.26E-04	0	0	R14009_38	8.59	1	0	2	MASCOT	T
R14009_38_3,14694	R.DKSIILWK.L	1002.59822	-0.00179	2	1	43.84	0	2.07E-06	0	0	R14009_38	8.59	1	0	2	MASCOT	T
R14009_38_3,14707	R.DKSIILWK.L	1002.59822	0.00028	3	1	28.25	0	7.48E-05	0	0	R14009_38	8.59	1	0	2	MASCOT	T
R14009_38_3,14727	R.DKSIILWK.L	1002.59822	-0.00349	2	1	37.56	0	8.77E-06	0	0	R14009_38	8.59	1	0	2	MASCOT	T
R14009_38_3,14740	R.DKSIILWK.L	1002.59822	0.00079	3	1	26.76	0	1.05E-04	0	0	R14009_38	8.59	1	0	2	MASCOT	T
R14009_38_3,14760	R.DKSIILWK.L	1002.59822	-0.00323	2	1	37.25	0	9.42E-06	0	0	R14009_38	8.59	1	0	2	MASCOT	T
R14009_38_3,14773	R.DKSIILWK.L	1002.59822	0.00037	3	1	24.16	0	1.92E-04	0	0	R14009_38	8.59	1	0	2	MASCOT	T
R14009_38_3,14793	R.DKSIILWK.L	1002.59822	-0.00317	2	1	48.64	0	6.84E-07	0	0	R14009_38	8.59	1	0	2	MASCOT	T
R14009_38_3,14806	R.DKSIILWK.L	1002.59822	0.00049	3	1	32.83	0	2.61E-05	0	0	R14009_38	8.59	1	0	2	MASCOT	T
R14009_38_3,14826	R.DKSIILWK.L	1002.59822	-0.00265	2	1	42.21	0	3.01E-06	0	0	R14009_38	8.59	1	0	2	MASCOT	T
R14009_38_3,14839	R.DKSIILWK.L	1002.59822	0.00055	3	1	23.58	0	2.19E-04	0	0	R14009_38	8.59	1	0	2	MASCOT	T
R14009_38_3,14859	R.DKSIILWK.L	1002.59822	-0.00287	2	1	48.65	0	6.82E-07	0	0	R14009_38	8.59	1	0	2	MASCOT	T
R14009_38_3,14872	R.DKSIILWK.L	1002.59822	0.00058	3	1	28.85	0	6.52E-05	0	0	R14009_38	8.59	1	0	2	MASCOT	T
R14009_38_3,14892	R.DKSIILWK.L	1002.59822	-0.00309	2	1	36.42	0	1.14E-05	0	0	R14009_38	8.59	1	0	2	MASCOT	T
R14009_38_3,14907	R.DKSIILWK.L	1002.59822	0.00061	3	1	28.17	0	7.62E-05	0	0	R14009_38	8.59	1	0	2	MASCOT	T
R14009_38_3,14925	R.DKSIILWK.L	1002.59822	-0.00169	2	1	38.31	0	7.38E-06	0	0	R14009_38	8.59	1	0	2	MASCOT	T
R14009_38_3,14958	R.DKSIILWK.L	1002.59822	-0.00059	2	1	38.73	0	6.70E-06	0	0	R14009_38	8.59	1	0	2	MASCOT	T
R14009_38_3,14993	R.DKSIILWK.L	1002.59822	-0.00025	2	1	40.46	0	4.50E-06	0	0	R14009_38	8.59	1	0	2	MASCOT	T
R14009_38_3,15028	R.DKSIILWK.L	1002.59822	-0.00089	2	1	38.82	0	6.56E-06	0	0	R14009_38	8.59	1	0	2	MASCOT	T
R14009_38_3,23851	R.FSPNTLVPTIVSASWDK.T	1861.96945	0.007	2	1	45.3	0	1.48E-06	0	0	R14009_38	5.84	0	0	2	MASCOT	T
R14009_38_3,23896	R.FSPNTLVPTIVSASWDK.T	1861.96945	0.00246	2	1	41.7	0	3.38E-06	0	0	R14009_38	5.84	0	0	2	MASCOT	T
R14009_38_3,23940	R.FSPNTLVPTIVSASWDK.T	1861.96945	-0.00008	2	1	38.95	0	6.37E-06	0	0	R14009_38	5.84	0	0	2	MASCOT	T
R14009_38_3,23983	R.FSPNTLVPTIVSASWDK.T	1861.96945	-0.00006	2	1	54.72	0	1.69E-07	0	0	R14009_38	5.84	0	0	2	MASCOT	T
R14009_38_3,24027	R.FSPNTLVPTIVSASWDK.T	1861.96945	-0.00118	2	1	53.73	0	2.12E-07	0	0	R14009_38	5.84	0	0	2	MASCOT	T
R14009_38_3,24432	R.FSPNTLVPTIVSASWDK.T	1861.96945	-0.00138	2	1	68.84	0	6.53E-09	0	0	R14009_38	5.84	0	0	2	MASCOT	T
R14009_38_3,24464	R.FSPNTLVPTIVSASWDK.T	1861.96945	-0.00162	2	1	57.19	0	9.55E-08	0	0	R14009_38	5.84	0	0	2	MASCOT	T
R14009_38_3,24497	R.FSPNTLVPTIVSASWDK.T	1861.96945	-0.00088	2	1	68.77	0	6.64E-09	0	0	R14009_38	5.84	0	0	2	MASCOT	T
R14009_38_3,24530	R.FSPNTLVPTIVSASWDK.T	1861.96945	-0.00074	2	1	79.2	0	6.01E-10	0	0	R14009_38	5.84	0	0	2	MASCOT	T
R14009_38_3,24561	R.FSPNTLVPTIVSASWDK.T	1861.96945	0.00096	2	1	83.79	0	2.09E-10	0	0	R14009_38	5.84	0	0	2	MASCOT	T
R14009_38_3,24566	R.FSPNTLVPTIVSASWDK.T	1861.96945	0.00135	3	1	34.49	0	1.78E-05	0	0	R14009_38	5.84	0	0	2	MASCOT	T
R14009_38_3,24594	R.FSPNTLVPTIVSASWDK.T	1861.96945	-0.00002	2	1	82.82	0	2.61E-10	0	0	R14009_38	5.84	0	0	2	MASCOT	T
R14009_38_3,24610	R.FSPNTLVPTIVSASWDK.T	1861.96945	0.00036	3	1	45.56	0	1.39E-06	0	0	R14009_38	5.84	0	0	2	MASCOT	T
R14009_38_3,24627	R.FSPNTLVPTIVSASWDK.T	1861.96945	-0.00012	2	1	69.59	0	5.50E-09	0	0	R14009_38	5.84	0	0	2	MASCOT	T
R14009_38_3,24655	R.FSPNTLVPTIVSASWDK.T	1861.96945	-0.00174	3	1	43.55	0	2.21E-06	0	0	R14009_38	5.84	0	0	2	MASCOT	T
R14009_38_3,24660	R.FSPNTLVPTIVSASWDK.T	1861.96945	0.00014	2	1	83	0	2.51E-10	0	0	R14009_38	5.84	0	0	2	MASCOT	T

R14009_38_3,24693	R.FSPNTLVPTIVSASWDK.T	1861.96945	0.00044	2	1	78.31	0	7.38E-10	0	0	R14009_38	5.84	0	0	2	MASCOT	T
R14009_38_3,24696	R.FSPNTLVPTIVSASWDK.T	1861.96945	-0.00096	3	1	40.4	0	4.56E-06	0	0	R14009_38	5.84	0	0	2	MASCOT	T
R14009_38_3,24726	R.FSPNTLVPTIVSASWDK.T	1861.96945	0.0008	2	1	76.97	0	1.00E-09	0	0	R14009_38	5.84	0	0	2	MASCOT	T
R14009_38_3,24727	R.FSPNTLVPTIVSASWDK.T	1861.96945	-0.00066	3	1	42.05	0	3.12E-06	0	0	R14009_38	5.84	0	0	2	MASCOT	T
R14009_38_3,24759	R.FSPNTLVPTIVSASWDK.T	1861.96945	0.00082	2	1	79.71	0	5.35E-10	0	0	R14009_38	5.84	0	0	2	MASCOT	T
R14009_38_3,24760	R.FSPNTLVPTIVSASWDK.T	1861.96945	-0.00015	3	1	43.67	0	2.15E-06	0	0	R14009_38	5.84	0	0	2	MASCOT	T
R14009_38_3,24791	R.FSPNTLVPTIVSASWDK.T	1861.96945	-0.0055	2	1	83.96	0	2.01E-10	0	0	R14009_38	5.84	0	0	2	MASCOT	T
R14009_38_3,24792	R.FSPNTLVPTIVSASWDK.T	1861.96945	-0.0006	3	1	43.33	0	2.32E-06	0	0	R14009_38	5.84	0	0	2	MASCOT	T
R14009_38_3,24824	R.FSPNTLVPTIVSASWDK.T	1861.96945	0.00098	2	1	83.19	0	2.40E-10	0	0	R14009_38	5.84	0	0	2	MASCOT	T
R14009_38_3,24826	R.FSPNTLVPTIVSASWDK.T	1861.96945	-0.00075	3	1	43.25	0	2.37E-06	0	0	R14009_38	5.84	0	0	2	MASCOT	T
R14009_38_3,24857	R.FSPNTLVPTIVSASWDK.T	1861.96945	-0.00584	2	1	83.93	0	2.02E-10	0	0	R14009_38	5.84	0	0	2	MASCOT	T
R14009_38_3,24858	R.FSPNTLVPTIVSASWDK.T	1861.96945	-0.00045	3	1	38.47	0	7.11E-06	0	0	R14009_38	5.84	0	0	2	MASCOT	T
R14009_38_3,24892	R.FSPNTLVPTIVSASWDK.T	1861.96945	-0.00021	3	1	64.65	0	1.71E-08	0	0	R14009_38	5.84	0	0	2	MASCOT	T
R14009_38_3,24893	R.FSPNTLVPTIVSASWDK.T	1861.96945	-0.00688	2	1	82.7	0	2.69E-10	0	0	R14009_38	5.84	0	0	2	MASCOT	T
R14009_38_3,24923	R.FSPNTLVPTIVSASWDK.T	1861.96945	-0.00642	2	1	83.88	0	2.05E-10	0	0	R14009_38	5.84	0	0	2	MASCOT	T
R14009_38_3,24927	R.FSPNTLVPTIVSASWDK.T	1861.96945	-0.00096	3	1	41.76	0	3.33E-06	0	0	R14009_38	5.84	0	0	2	MASCOT	T
R14009_38_3,24956	R.FSPNTLVPTIVSASWDK.T	1861.96945	-0.00536	2	1	84.86	0	1.63E-10	0	0	R14009_38	5.84	0	0	2	MASCOT	T
R14009_38_3,24971	R.FSPNTLVPTIVSASWDK.T	1861.96945	0.00024	3	1	53.17	0	2.41E-07	0	0	R14009_38	5.84	0	0	2	MASCOT	T
R14009_38_3,24989	R.FSPNTLVPTIVSASWDK.T	1861.96945	-0.00004	2	1	76.66	0	1.08E-09	0	0	R14009_38	5.84	0	0	2	MASCOT	T
R14009_38_3,25020	R.FSPNTLVPTIVSASWDK.T	1861.96945	0.00072	3	1	28.47	0	7.11E-05	0	0	R14009_38	5.84	0	0	2	MASCOT	T
R14009_38_3,25022	R.FSPNTLVPTIVSASWDK.T	1861.96945	-0.0008	2	1	87.9	0	8.11E-11	0	0	R14009_38	5.84	0	0	2	MASCOT	T
R14009_38_3,25055	R.FSPNTLVPTIVSASWDK.T	1861.96945	-0.00194	2	1	71.97	0	3.18E-09	0	0	R14009_38	5.84	0	0	2	MASCOT	T
R14009_38_3,25088	R.FSPNTLVPTIVSASWDK.T	1861.96945	-0.00158	2	1	78.22	0	7.53E-10	0	0	R14009_38	5.84	0	0	2	MASCOT	T
R14009_38_3,25122	R.FSPNTLVPTIVSASWDK.T	1861.96945	-0.00218	2	1	73.19	0	2.40E-09	0	0	R14009_38	5.84	0	0	2	MASCOT	T
R14009_38_3,25156	R.FSPNTLVPTIVSASWDK.T	1861.96945	-0.0031	2	1	33.75	0	2.11E-05	0	0	R14009_38	5.84	0	0	2	MASCOT	T
R14009_38_3,25188	R.FSPNTLVPTIVSASWDK.T	1861.96945	-0.00274	2	1	31.27	0	3.73E-05	0	0	R14009_38	5.84	0	0	2	MASCOT	T
R14009_38_3,25226	R.FSPNTLVPTIVSASWDK.T	1861.96945	-0.00056	2	1	36.61	0	1.09E-05	0	0	R14009_38	5.84	0	0	2	MASCOT	T
R14009_38_3,25269	R.FSPNTLVPTIVSASWDK.T	1861.96945	-0.00264	2	1	24.03	0	1.98E-04	0	0	R14009_38	5.84	0	0	2	MASCOT	T
R14009_38_3,25381	R.FSPNTLVPTIVSASWDK.T	1861.96945	0.0021	2	1	38.71	0	6.73E-06	0	0	R14009_38	5.84	0	0	2	MASCOT	T
R14009_38_3,25424	R.FSPNTLVPTIVSASWDK.T	1861.96945	-0.0004	2	1	46.03	0	1.25E-06	0	0	R14009_38	5.84	0	0	2	MASCOT	T
R14009_38_3,13282	R.IWDLESK.S	890.46179	0.00091	2	1	24.08	0	1.95E-04	0	0	R14009_38	4.37	0	0	2	MASCOT	T
R14009_38_3,13322	R.IWDLESK.S	890.46179	-0.00011	2	1	28.57	0	6.95E-05	0	0	R14009_38	4.37	0	0	2	MASCOT	T
R14009_38_3,13366	R.IWDLESK.S	890.46179	-0.00025	2	1	28.67	0	6.79E-05	0	0	R14009_38	4.37	0	0	2	MASCOT	T
R14009_38_3,13408	R.IWDLESK.S	890.46179	0.00003	2	1	28.79	0	6.61E-05	0	0	R14009_38	4.37	0	0	2	MASCOT	T
R14009_38_3,13440	R.IWDLESK.S	890.46179	0.00125	2	1	28.79	0	6.61E-05	0	0	R14009_38	4.37	0	0	2	MASCOT	T
R14009_38_3,13473	R.IWDLESK.S	890.46179	0.00201	2	1	28.76	0	6.65E-05	0	0	R14009_38	4.37	0	0	2	MASCOT	T
R14009_38_3,13506	R.IWDLESK.S	890.46179	0.00241	2	1	28.82	0	6.56E-05	0	0	R14009_38	4.37	0	0	2	MASCOT	T
R14009_38_3,13539	R.IWDLESK.S	890.46179	0.00295	2	1	26.71	0	1.07E-04	0	0	R14009_38	4.37	0	0	2	MASCOT	T
R14009_38_3,13572	R.IWDLESK.S	890.46179	0.00305	2	1	28.79	0	6.61E-05	0	0	R14009_38	4.37	0	0	2	MASCOT	T
R14009_38_3,13605	R.IWDLESK.S	890.46179	0.00317	2	1	28.77	0	6.64E-05	0	0	R14009_38	4.37	0	0	2	MASCOT	T
R14009_38_3,13638	R.IWDLESK.S	890.46179	0.00269	2	1	28.8	0	6.59E-05	0	0	R14009_38	4.37	0	0	2	MASCOT	T
R14009_38_3,13671	R.IWDLESK.S	890.46179	0.00267	2	1	28.02	0	7.89E-05	0	0	R14009_38	4.37	0	0	2	MASCOT	T
R14009_38_3,13704	R.IWDLESK.S	890.46179	0.00197	2	1	28.79	0	6.61E-05	0	0	R14009_38	4.37	0	0	2	MASCOT	T
R14009_38_3,13738	R.IWDLESK.S	890.46179	0.00113	2	1	28.7	0	6.74E-05	0	0	R14009_38	4.37	0	0	2	MASCOT	T
R14009_38_3,13771	R.IWDLESK.S	890.46179	0.00007	2	1	28.47	0	7.11E-05	0	0	R14009_38	4.37	0	0	2	MASCOT	T
R14009_38_3,13805	R.IWDLESK.S	890.46179	0.00015	2	1	28.59	0	6.92E-05	0	0	R14009_38	4.37	0	0	2	MASCOT	T
R14009_38_3,13839	R.IWDLESK.S	890.46179	-0.00005	2	1	30.29	0	4.68E-05	0	0	R14009_38	4.37	0	0	2	MASCOT	T
R14009_38_3,13871	R.IWDLESK.S	890.46179	-0.00029	2	1	23.54	0	2.21E-04	0	0	R14009_38	4.37	0	0	2	MASCOT	T
R14009_38_3,13909	R.IWDLESK.S	890.46179	0.00001	2	1	26.76	0	1.05E-04	0	0	R14009_38	4.37	0	0	2	MASCOT	T
R14009_38_3,14172	R.LWDLATGESTR.R	1248.62189	-0.0001	2	1	38.11	0	7.73E-06	0	0	R14009_38	4.37	0	0	2	MASCOT	T
R14009_38_3,15393	R.LWDLATGESTR.R	1248.62189	-0.00106	2	1	53.99	0	2.00E-07	0	0	R14009_38	4.37	0	0	2	MASCOT	T
R14009_38_3,15431	R.LWDLATGESTR.R	1248.62189	-0.0004	2	1	43.71	0	2.13E-06	0	0	R14009_38	4.37	0	0	2	MASCOT	T
R14009_38_3,15464	R.LWDLATGESTR.R	1248.62189	-0.00114	2	1	47.29	0	9.33E-07	0	0	R14009_38	4.37	0	0	2	MASCOT	T
R14009_38_3,15497	R.LWDLATGESTR.R	1248.62189	-0.00156	2	1	49.2	0	6.01E-07	0	0	R14009_38	4.37	0	0	2	MASCOT	T
R14009_38_3,15530	R.LWDLATGESTR.R	1248.62189	-0.00228	2	1	47.39	0	9.12E-07	0	0	R14009_38	4.37	0	0	2	MASCOT	T
R14009_38_3,15587	R.LWDLATGESTR.R	1248.62189	-0.00336	2	1	47.42	0	9.06E-07	0	0	R14009_38	4.37	0	0	2	MASCOT	T
R14009_38_3,15764	R.LWDLATGESTR.R	1248.62189	-0.00376	2	1	47	0	9.98E-07	0	0	R14009_38	4.37	0	0	2	MASCOT	T
R14009_38_3,15794	R.LWDLATGESTR.R	1248.62189	-0.0032	2	1	48.6	0	6.90E-07	0	0	R14009_38	4.37	0	0	2	MASCOT	T
R14009_38_3,15827	R.LWDLATGESTR.R	1248.62189	-0.0009	2	1	49.27	0	5.92E-07	0	0	R14009_38	4.37	0	0	2	MASCOT	T
R14009_38_3,15861	R.LWDLATGESTR.R	1248.62189	0.00004	2	1	53.29	0	2.34E-07	0	0	R14009_38	4.37	0	0	2	MASCOT	T
R14009_38_3,15894	R.LWDLATGESTR.R	1248.62189	0.00004	2	1	53.91	0	2.03E-07	0	0	R14009_38	4.37	0	0	2	MASCOT	T
R14009_38_3,15928	R.LWDLATGESTR.R	1248.62189	0.00024	2	1	62.64	0	2.72E-08	0	0	R14009_38	4.37	0	0	2	MASCOT	T
R14009_38_3,15964	R.LWDLATGESTR.R	1248.62189	0.00104	2	1	52.18	0	3.03E-07	0	0	R14009_38	4.37	0	0	2	MASCOT	T
R14009_38_3,16012	R.LWDLATGESTR.R	1248.62189	0.00142	2	1	62.11	0	3.08E-08	0	0	R14009_38	4.37	0	0	2	MASCOT	T
R14009_38_3,16055	R.LWDLATGESTR.R	1248.62189	0.00252	2	1	47.7	0	8.49E-07	0	0	R14009_38	4.37	0	0	2	MASCOT	T
R14009_38_3,16098	R.LWDLATGESTR.R	1248.62189	0.00274	2	1	62.24	0	2.99E-08	0	0	R14009_38	4.37	0	0	2	MASCOT	T
R14009_38_3,16144	R.LWDLATGESTR.R	1248.62189	0.00282	2	1	44.34	0	1.84E-06	0	0	R14009_38	4.37	0	0	2	MASCOT	T
R14009_38_3,16185	R.LWDLATGESTR.R	1248.62189	0.0029	2	1	37.02	0	9.93E-06	0	0	R14009_38	4.37	0	0	2	MASCOT	T
R14009_38_3,16231	R.LWDLATGESTR.R	1248.62189	0.00388	2	1	20.29	0	4.68E-04	0	0	R14009_38	4.37	0	0	2	MASCOT	T
R14009_38_3,16274	R.LWDLATGESTR.R	1248.62189	0.00308	2	1	33.76	0	2.10E-05	0	0	R14009_38	4.37	0	0	2	MASCOT	T
R14009_38_3,16375	R.LWDLATGESTR.R	1248.62189	0.0023	2	1	46.43	0	1.14E-06	0	0	R14009_38	4.37	0	0	2	MASCOT	T
R14009_38_3,16416	R.LWDLATGESTR.R	1248.62189	0.00248	2	1	35.99	0	1.26E-05	0	0	R14009_38	4.37	0	0	2	MASCOT	T
R14009_38_3,13321	R.LWDLATGESTRR.F	1404.72299	0.00069	2	1	40.86	0	4.10E-06	0	0	R14009_38	6.07	1	0	2	MASCOT	T

R14009_38_3,13332	R.LWDLATGESTRR.F	1404.72299	-0.00158	3	1	25.9	0	1.29E-04	0|0	R14009_38	6.07	1	0	2 MASCOT	T
R14009_38_3,13352	R.LWDLATGESTRR.F	1404.72299	0.00129	2	1	39.15	0	6.08E-06	0|0	R14009_38	6.07	1	0	2 MASCOT	T
R14009_38_3,13365	R.LWDLATGESTRR.F	1404.72299	-0.00212	3	1	24.9	0	1.62E-04	0|0	R14009_38	6.07	1	0	2 MASCOT	T
R14009_38_3,13385	R.LWDLATGESTRR.F	1404.72299	0.00133	2	1	44.5	0	1.77E-06	0|0	R14009_38	6.07	1	0	2 MASCOT	T
R14009_38_3,13396	R.LWDLATGESTRR.F	1404.72299	-0.00164	3	1	26.74	0	1.06E-04	0|0	R14009_38	6.07	1	0	2 MASCOT	T
R14009_38_3,13418	R.LWDLATGESTRR.F	1404.72299	0.00305	2	1	35.62	0	1.37E-05	0|0	R14009_38	6.07	1	0	2 MASCOT	T
R14009_38_3,13429	R.LWDLATGESTRR.F	1404.72299	0.00088	3	1	24.86	0	1.63E-04	0|0	R14009_38	6.07	1	0	2 MASCOT	T
R14009_38_3,13451	R.LWDLATGESTRR.F	1404.72299	0.00517	2	1	39.05	0	6.22E-06	0|0	R14009_38	6.07	1	0	2 MASCOT	T
R14009_38_3,13462	R.LWDLATGESTRR.F	1404.72299	0.00304	3	1	24.91	0	1.61E-04	0|0	R14009_38	6.07	1	0	2 MASCOT	T
R14009_38_3,13484	R.LWDLATGESTRR.F	1404.72299	0.00653	2	1	41.33	0	3.68E-06	0|0	R14009_38	6.07	1	0	2 MASCOT	T
R14009_38_3,13495	R.LWDLATGESTRR.F	1404.72299	0.00457	3	1	24.88	0	1.63E-04	0|0	R14009_38	6.07	1	0	2 MASCOT	T
R14009_38_3,13517	R.LWDLATGESTRR.F	1404.72299	0.00791	2	1	39.41	0	5.73E-06	0|0	R14009_38	6.07	1	0	2 MASCOT	T
R14009_38_3,13528	R.LWDLATGESTRR.F	1404.72299	0.00547	3	1	23.93	0	2.02E-04	0|0	R14009_38	6.07	1	0	2 MASCOT	T
R14009_38_3,13550	R.LWDLATGESTRR.F	1404.72299	0.01003	2	1	36.65	0	1.08E-05	0|0	R14009_38	6.07	1	0	2 MASCOT	T
R14009_38_3,13561	R.LWDLATGESTRR.F	1404.72299	0.0064	3	1	26.4	0	1.15E-04	0|0	R14009_38	6.07	1	0	2 MASCOT	T
R14009_38_3,13583	R.LWDLATGESTRR.F	1404.72299	0.01005	2	1	38.65	0	6.82E-06	0|0	R14009_38	6.07	1	0	2 MASCOT	T
R14009_38_3,13595	R.LWDLATGESTRR.F	1404.72299	0.00775	3	1	26.78	0	1.05E-04	0|0	R14009_38	6.07	1	0	2 MASCOT	T
R14009_38_3,13616	R.LWDLATGESTRR.F	1404.72299	0.00957	2	1	46.71	0	1.07E-06	0|0	R14009_38	6.07	1	0	2 MASCOT	T
R14009_38_3,13628	R.LWDLATGESTRR.F	1404.72299	0.00802	3	1	30.95	0	4.02E-05	0|0	R14009_38	6.07	1	0	2 MASCOT	T
R14009_38_3,13650	R.LWDLATGESTRR.F	1404.72299	0.00965	2	1	31.36	0	3.66E-05	0|0	R14009_38	6.07	1	0	2 MASCOT	T
R14009_38_3,13666	R.LWDLATGESTRR.F	1404.72299	0.00775	3	1	24.56	0	1.75E-04	0|0	R14009_38	6.07	1	0	2 MASCOT	T
R14009_38_3,13689	R.LWDLATGESTRR.F	1404.72299	0.00853	2	1	29.35	0	5.81E-05	0|0	R14009_38	6.07	1	0	2 MASCOT	T
R14009_38_3,15481	R.NTLAGHSGYLNTVAVSPDGS	2633.26242	0.0047	3	1	61.56	0	3.49E-08	0|0	R14009_38	6.73	0	0	2 MASCOT	T
R14009_38_3,15521	R.NTLAGHSGYLNTVAVSPDGS	2633.26242	0.00155	3	1	79.71	0	5.35E-10	0|0	R14009_38	6.73	0	0	2 MASCOT	T
R14009_38_3,15554	R.NTLAGHSGYLNTVAVSPDGS	2633.26242	0.00164	3	1	79.16	0	6.07E-10	0|0	R14009_38	6.73	0	0	2 MASCOT	T
R14009_38_3,15588	R.NTLAGHSGYLNTVAVSPDGS	2633.26242	0.00083	3	1	89.62	0	5.46E-11	0|0	R14009_38	6.73	0	0	2 MASCOT	T
R14009_38_3,15620	R.NTLAGHSGYLNTVAVSPDGS	2633.26242	-0.00022	3	1	88.95	0	6.37E-11	0|0	R14009_38	6.73	0	0	2 MASCOT	T
R14009_38_3,15652	R.NTLAGHSGYLNTVAVSPDGS	2633.26242	-0.00049	3	1	94.46	0	1.79E-11	0|0	R14009_38	6.73	0	0	2 MASCOT	T
R14009_38_3,15685	R.NTLAGHSGYLNTVAVSPDGS	2633.26242	-0.00043	3	1	83.4	0	2.29E-10	0|0	R14009_38	6.73	0	0	2 MASCOT	T
R14009_38_3,15718	R.NTLAGHSGYLNTVAVSPDGS	2633.26242	-0.00094	3	1	92.62	0	2.74E-11	0|0	R14009_38	6.73	0	0	2 MASCOT	T
R14009_38_3,15732	R.NTLAGHSGYLNTVAVSPDGS	2633.26242	-0.00175	2	1	40.46	0	4.50E-06	0|0	R14009_38	6.73	0	0	2 MASCOT	T
R14009_38_3,15752	R.NTLAGHSGYLNTVAVSPDGS	2633.26242	-0.00256	3	1	87.42	0	9.06E-11	0|0	R14009_38	6.73	0	0	2 MASCOT	T
R14009_38_3,15785	R.NTLAGHSGYLNTVAVSPDGS	2633.26242	-0.00313	3	1	93.65	0	2.16E-11	0|0	R14009_38	6.73	0	0	2 MASCOT	T
R14009_38_3,15818	R.NTLAGHSGYLNTVAVSPDGS	2633.26242	-0.00283	3	1	84.84	0	1.64E-10	0|0	R14009_38	6.73	0	0	2 MASCOT	T
R14009_38_3,15851	R.NTLAGHSGYLNTVAVSPDGS	2633.26242	-0.00121	3	1	99.06	0	6.21E-12	0|0	R14009_38	6.73	0	0	2 MASCOT	T
R14009_38_3,15896	R.NTLAGHSGYLNTVAVSPDGS	2633.26242	-0.00106	3	1	99.46	0	5.66E-12	0|0	R14009_38	6.73	0	0	2 MASCOT	T
R14009_38_3,15929	R.NTLAGHSGYLNTVAVSPDGS	2633.26242	-0.00205	3	1	91.06	0	3.92E-11	0|0	R14009_38	6.73	0	0	2 MASCOT	T
R14009_38_3,15963	R.NTLAGHSGYLNTVAVSPDGS	2633.26242	-0.0001	3	1	89.41	0	5.73E-11	0|0	R14009_38	6.73	0	0	2 MASCOT	T
R14009_38_3,16008	R.NTLAGHSGYLNTVAVSPDGS	2633.26242	-0.00139	3	1	86.85	0	1.03E-10	0|0	R14009_38	6.73	0	0	2 MASCOT	T
R14009_38_3,16052	R.NTLAGHSGYLNTVAVSPDGS	2633.26242	-0.001	3	1	75.98	0	1.26E-09	0|0	R14009_38	6.73	0	0	2 MASCOT	T
R14009_38_3,16095	R.NTLAGHSGYLNTVAVSPDGS	2633.26242	-0.00031	3	1	80.15	0	4.83E-10	0|0	R14009_38	6.73	0	0	2 MASCOT	T
R14009_38_3,16141	R.NTLAGHSGYLNTVAVSPDGS	2633.26242	-0.00028	3	1	69.43	0	5.70E-09	0|0	R14009_38	6.73	0	0	2 MASCOT	T
R14009_38_3,16198	R.NTLAGHSGYLNTVAVSPDGS	2633.26242	-0.0019	3	1	60.17	0	4.81E-08	0|0	R14009_38	6.73	0	0	2 MASCOT	T
R14009_38_3,16240	R.NTLAGHSGYLNTVAVSPDGS	2633.26242	-0.00115	3	1	52.55	0	2.78E-07	0|0	R14009_38	6.73	0	0	2 MASCOT	T
R14009_38_3,16320	R.NTLAGHSGYLNTVAVSPDGS	2633.26242	-0.00055	3	1	65.44	0	1.43E-08	0|0	R14009_38	6.73	0	0	2 MASCOT	T
R14009_38_3,16417	R.NTLAGHSGYLNTVAVSPDGS	2633.26242	0.00113	3	1	51.03	0	3.94E-07	0|0	R14009_38	6.73	0	0	2 MASCOT	T
R14009_38_3,3220	R.QIVSASR.D	760.43117	0.00016	2	1	39.92	0	5.09E-06	0|0	R14009_38	9.75	0	0	2 MASCOT	T
R14009_38_3,3260	R.QIVSASR.D	760.43117	0.00036	2	1	48.29	0	7.41E-07	0|0	R14009_38	9.75	0	0	2 MASCOT	T
R14009_38_3,3293	R.QIVSASR.D	760.43117	0.00026	2	1	49.8	0	5.24E-07	0|0	R14009_38	9.75	0	0	2 MASCOT	T
R14009_38_3,3325	R.QIVSASR.D	760.43117	0.0001	2	1	46.61	0	1.09E-06	0|0	R14009_38	9.75	0	0	2 MASCOT	T
R14009_38_3,3350	R.QIVSASR.D	760.43117	0	2	1	46.91	0	1.02E-06	0|0	R14009_38	9.75	0	0	2 MASCOT	T
R14009_38_3,3375	R.QIVSASR.D	760.43117	-0.0001	2	1	48.05	0	7.83E-07	0|0	R14009_38	9.75	0	0	2 MASCOT	T
R14009_38_3,3403	R.QIVSASR.D	760.43117	0.00004	2	1	48.3	0	7.40E-07	0|0	R14009_38	9.75	0	0	2 MASCOT	T
R14009_38_3,3436	R.QIVSASR.D	760.43117	0.0003	2	1	49.78	0	5.26E-07	0|0	R14009_38	9.75	0	0	2 MASCOT	T
R14009_38_3,3469	R.QIVSASR.D	760.43117	0.00036	2	1	45.8	0	1.32E-06	0|0	R14009_38	9.75	0	0	2 MASCOT	T
R14009_38_3,3504	R.QIVSASR.D	760.43117	0.00036	2	1	34.68	0	1.70E-05	0|0	R14009_38	9.75	0	0	2 MASCOT	T
R14009_38_3,3540	R.QIVSASR.D	760.43117	0.00092	2	1	31.67	0	3.40E-05	0|0	R14009_38	9.75	0	0	2 MASCOT	T
R14009_38_3,11705	R.VWGIGR.Y	687.39367	0.00051	2	1	20.37	0	4.59E-04	0|0	R14009_38	9.72	0	0	2 MASCOT	T
R14009_38_3,11737	R.VWGIGR.Y	687.39367	0.00035	2	1	20.37	0	4.59E-04	0|0	R14009_38	9.72	0	0	2 MASCOT	T
R14009_38_3,11802	R.VWGIGR.Y	687.39367	-0.00033	2	1	20.4	0	4.56E-04	0|0	R14009_38	9.72	0	0	2 MASCOT	T
R14009_38_3,11834	R.VWGIGR.Y	687.39367	-0.00065	2	1	20.37	0	4.59E-04	0|0	R14009_38	9.72	0	0	2 MASCOT	T
R14009_38_3,11867	R.VWGIGR.Y	687.39367	-0.00067	2	1	20.39	0	4.57E-04	0|0	R14009_38	9.72	0	0	2 MASCOT	T
R14009_38_3,11901	R.VWGIGR.Y	687.39367	-0.00031	2	1	20.38	0	4.58E-04	0|0	R14009_38	9.72	0	0	2 MASCOT	T
R14009_38_3,11934	R.VWGIGR.Y	687.39367	-0.00053	2	1	20.38	0	4.58E-04	0|0	R14009_38	9.72	0	0	2 MASCOT	T
R14009_38_3,11967	R.VWGIGR.Y	687.39367	-0.00037	2	1	20.4	0	4.56E-04	0|0	R14009_38	9.72	0	0	2 MASCOT	T
R14009_38_3,12033	R.VWGIGR.Y	687.39367	-0.00001	2	1	20.39	0	4.57E-04	0|0	R14009_38	9.72	0	0	2 MASCOT	T
R14009_38_3,12066	R.VWGIGR.Y	687.39367	0.00003	2	1	20.33	0	4.63E-04	0|0	R14009_38	9.72	0	0	2 MASCOT	T
R14009_38_3,12134	R.VWGIGR.Y	687.39367	0.00017	2	1	20.39	0	4.57E-04	0|0	R14009_38	9.72	0	0	2 MASCOT	T
R14009_38_3,17558	R.YWLCAATENSIR.I	1483.6998	0.00051	2	1	37	0	9.98E-06	0|0	R14009_38	5.99	0	0	2 MASCOT	T
R14009_38_3,17601	R.YWLCAATENSIR.I	1483.6998	0.00041	2	1	52.62	0	2.74E-07	0|0	R14009_38	5.99	0	0	2 MASCOT	T
R14009_38_3,17632	R.YWLCAATENSIR.I	1483.6998	-0.00021	2	1	59.9	0	5.12E-08	0|0	R14009_38	5.99	0	0	2 MASCOT	T
R14009_38_3,17664	R.YWLCAATENSIR.I	1483.6998	-0.00021	2	1	52.7	0	2.69E-07	0|0	R14009_38	5.99	0	0	2 MASCOT	T
R14009_38_3,17697	R.YWLCAATENSIR.I	1483.6998	0.00057	2	1	56.39	0	1.15E-07	0|0	R14009_38	5.99	0	0	2 MASCOT	T

R14009_38_3,17730	R.YWLCAATENSIR.I	1483.6998	-0.00019	2	1	59.99	0	5.01E-08	0	0	R14009_38	5.99	0	0	2	MASCOT	T
R14009_38_3,17763	R.YWLCAATENSIR.I	1483.6998	0.00057	2	1	58.72	0	6.71E-08	0	0	R14009_38	5.99	0	0	2	MASCOT	T
R14009_38_3,17796	R.YWLCAATENSIR.I	1483.6998	-0.00049	2	1	52.52	0	2.80E-07	0	0	R14009_38	5.99	0	0	2	MASCOT	T
R14009_38_3,17829	R.YWLCAATENSIR.I	1483.6998	-0.00011	2	1	52.35	0	2.91E-07	0	0	R14009_38	5.99	0	0	2	MASCOT	T
R14009_38_3,17863	R.YWLCAATENSIR.I	1483.6998	0.00039	2	1	52.25	0	2.98E-07	0	0	R14009_38	5.99	0	0	2	MASCOT	T
R14009_38_3,17897	R.YWLCAATENSIR.I	1483.6998	0.00069	2	1	41.04	0	3.94E-06	0	0	R14009_38	5.99	0	0	2	MASCOT	T
R14009_38_3,17934	R.YWLCAATENSIR.I	1483.6998	0.00073	2	1	39.36	0	5.79E-06	0	0	R14009_38	5.99	0	0	2	MASCOT	T
R14009_38_3,17989	R.YWLCAATENSIR.I	1483.6998	0.00145	2	1	40.72	0	4.24E-06	0	0	R14009_38	5.99	0	0	2	MASCOT	T
R14009_38_3,18034	R.YWLCAATENSIR.I	1483.6998	-0.00181	2	1	45.72	0	1.34E-06	0	0	R14009_38	5.99	0	0	2	MASCOT	T

----- summary -----

Total protein : 1

Total protein grou : 1

UniPepCount	ProteinGroupCount	Percent	ProteinCount	Percent
30	1	100.00%	1	100.00%
