# Supplementary material for: Sumoylation stabilizes RACK1B and enhance its interaction with RAP2.6 in the abscisic acid response
Source: Sci Rep. 2017 Mar 8;7:44090. doi: 10.1038/srep44090 (PMC5341030; doi:10.1038/srep44090)
Supplement: Supplementary Table S2 [file srep44090-s3.pdf]

Supplementary Table S2. MS scan for sumoylation of RACK1B *in vivo*

	Reference	PepCount	UniquePepCount	CoverPercent	MW	PI													
	FileScan	Sequence	MH+	Diff(MH+)	Charge	Rank	Score	DeltaScore	ExpectValue	Ions	Reference	DIFF_MOD	PI	MissCleavage	Modification	MatchedTIC	NumProteaseTermini	Engine	Decoy
$1-1	AtRACK1B	15	10	39.57%	35800.11	6.66													
	R15025_10_1_2,22656	K.DGVILLWDLAEGK.K	1428.7733	-0.00043	2	1	26.27	0	1.18E-04	0|0	AtRACK1B		4.03	0		0	2	MASCOT	T
	R15025_10_1_2,15528	K.DVLSVAFSTDNR.Q	1323.65393	0.00058	2	1	33.93	0	2.02E-05	0|0	AtRACK1B		4.21	0		0	2	MASCOT	T
	R15025_10_1_2,15619	K.DVLSVAFSTDNR.Q	1323.65393	0.00028	2	1	65.48	0	1.42E-08	0|0	AtRACK1B		4.21	0		0	2	MASCOT	T
	R15025_10_1_2,11744	K.LWNTLGECK.Y	1120.54555	-0.00012	2	1	33.48	0	4.49E-05	0|0	AtRACK1B		5.99	0		0	2	MASCOT	T
	R15025_10_1_2,11840	K.LWNTLGECK.Y	1120.54555	0.00036	2	1	32.82	0	5.22E-05	0|0	AtRACK1B		5.99	0		0	2	MASCOT	T
	R15025_10_1_2,13864	K.SVVEDLKVDLK.A	1244.70964	-0.00009	2	1	36.87	0	2.06E-05	0|0	AtRACK1B		4.56	1		0	2	MASCOT	T
	R15025_10_1_2,6967	K.YTISEADGHK.E	1120.52691	0.00033	2	1	33.39	0	4.58E-05	0|0	AtRACK1B		5.32	0		0	2	MASCOT	T
	R15025_10_1_2,11189	K.YTISEADGHKEWVSCVR.F	2036.94945	0.00059	3	1	32.3	0	2.94E-05	0|0	AtRACK1B		5.45	1		0	2	MASCOT	T
	R15025_10_1_2,20747	R.FSPNTLVPTIVSASWDK.T	1861.96945	-0.00048	2	1	35.2	0	1.51E-05	0|0	AtRACK1B		5.84	0		0	2	MASCOT	T
	R15025_10_1_2,13652	R.LWDLATGESTR.R	1248.62189	0.0005	2	1	63.83	0	2.07E-08	0|0	AtRACK1B		4.37	0		0	2	MASCOT	T
	R15025_10_1_2,13741	R.LWDLATGESTR.R	1248.62189	0.00032	2	1	45.45	0	1.43E-06	0|0	AtRACK1B		4.37	0		0	2	MASCOT	T
	R15025_10_1_2,13839	R.LWDLATGESTR.R	1248.62189	0.00002	2	1	29.3	0	5.87E-05	0|0	AtRACK1B		4.37	0		0	2	MASCOT	T
	R15025_10_1_2,13931	R.NTLAGHSGYLNTVAVSPDGSLCASGGK.[illegible]	2633.26242	0.00179	3	1	44.36	0	1.83E-06	0|0	AtRACK1B		6.73	0		0	2	MASCOT	T
	R15025_10_1_2,14583	R.YWLCAATENSIR.I	1483.6998	0.00095	2	1	50.84	0	4.12E-07	0|0	AtRACK1B		5.99	0		0	2	MASCOT	T
	R15025_10_1_2,14678	R.YWLCAATENSIR.I	1483.6998	0.00055	2	1	35.38	0	1.45E-05	0|0	AtRACK1B		5.99	0		0	2	MASCOT	T
$2-1	AtSUMO_1_H89R	8	3	29.03%	10493.61	4.93													
	R15025_10_1_2,11835	K.GQDGNEVFFR.I	1168.53818	0.00011	2	1	64.14	0	1.93E-08	0|0	AtSUMO_1_H89R		4.37	0		0	2	MASCOT	T
	R15025_10_1_2,11926	K.GQDGNEVFFR.I	1168.53818	-0.00001	2	1	64.22	0	1.89E-08	0|0	AtSUMO_1_H89R		4.37	0		0	2	MASCOT	T
	R15025_10_1_2,12014	K.GQDGNEVFFR.I	1168.53818	-0.00011	2	1	64.46	0	1.79E-08	0|0	AtSUMO_1_H89R		4.37	0		0	2	MASCOT	T
	R15025_10_1_2,12105	K.GQDGNEVFFR.I	1168.53818	0.00017	2	1	51.16	0	3.83E-07	0|0	AtSUMO_1_H89R		4.37	0		0	2	MASCOT	T
	R15025_10_1_2,12204	K.GQDGNEVFFR.I	1168.53818	-0.00009	2	1	63.16	0	2.42E-08	0|0	AtSUMO_1_H89R		4.37	0		0	2	MASCOT	T
	R15025_10_1_2,10685	K.VKGQDGNEVFFR.I	1395.70155	-0.00021	2	1	48.88	0	6.47E-07	0|0	AtSUMO_1_H89R		6.04	1		0	2	MASCOT	T
	R15025_10_1_2,10775	K.VKGQDGNEVFFR.I	1395.70155	0.00091	2	1	77.46	0	8.97E-10	0|0	AtSUMO_1_H89R		6.04	1		0	2	MASCOT	T
	R15025_10_1_2,21190	R.QSVDMNSIAFLFDGR.R	1699.81083	0.00111	2	1	54.21	0	1.90E-07	0|0	AtSUMO_1_H89R		4.21	0		0	2	MASCOT	T

----- summary -----

Total protein: 2

Total protein: 2

UniPepCount	ProteinGroupCount	Percent	ProteinCount	Percent
3	1	50.00%	1	29.03%
10	1	50.00%	1	39.57%
