# Supplementary material for: Sumoylation stabilizes RACK1B and enhance its interaction with RAP2.6 in the abscisic acid response
Source: Sci Rep. 2017 Mar 8;7:44090. doi: 10.1038/srep44090 (PMC5341030; doi:10.1038/srep44090)
Supplement: Supplementary Table S3 [file srep44090-s4.pdf]

Supplementary Table S3. Primers used in this study

Purpose	Gene	Terminal vector	Name	Sequence (5'->3')
Recombinant protein expression	SUM1	pCDFDuet-1 pGreenII 62-SK	SUM1-F	ATGTCTGCAAACCAGGAGGAAGAC
			SUM1(GG)-R	TCAGGCCGTAGCACCACC
			SUM1(AA)-R	TCAAGCAGCAGTCTGATGGAG
			SUM1(QTGG)-R	TCAGCCACCAGTCTGATGGAGCA
	SUM3	pCDFDuet-1	SUM3-F	ATGTCTAACCCTCAAGATGACAAGC
			SUM3 (GG)-R	TTAACCACCACTCATCGCCCG
	SUM5	pCDFDuet-1	SUM5-F	ATGGTGAGTTCCACAGACACAATCT
			SUM5 (GG)-R	TCAGCCACCACCAAGTTCCATG
	UBQ	pGreenII 62-SK	UBQ-F	ATGCAGATCTTCGTTAAGACTCTCA
			UBQ-R	TTAGAAACCACCACGGAGACG
	SAE1B	pACYCDuet-1	SAE1B-F	ATGGACGGAGATGAGCTCACC
			SAE1B-R	TTAAAGCTTGTGGGATAGGTCCTC
	SAE2	pACYCDuet-1	SAE2-F	ATGGCTACGCAACAACAGC
			SAE2-R	CTATTCAACTCTTATCTTCTTT
	SCE1A	pCDFDuet-1 pGS-21a	SCE1A-F	ATGCGCTAGTGGAATCGCTCGTG
			SCE1A-R	TTAGACAAGAGCAGGATACTGCTTGGA
	SIZ1	pGS-21a	SIZ1-F	ATGGATTTGGAAGCTAATTGTAAGG
			SIZ1-R	TTAAACTCCGGTGTCTTGTCTGATG
	HPY2/MMS 21	pGreenII 62-SK	HPY2-F	ATGGCGTCGGCGTCCTCGTCTGACG
			HPY2-R	CTAATCTTCATCCACATCTTCTGTG
	RACK1A	pET28a	R1A-F	ATGGCGGAAGGACTCGTTTTG
			R1A-R	CTAGTAACGACCAATACCCCAAAC
	RACK1C	pET28a	R1C-F	ATGGCCGAGGGACTCGTATTG
			R1C-R	CTAGTAACGACCAATACCCCAAAC
	RAP2.6	pET28a pGreenII 62-SK	2.6-F	ATGGTGTCTATGCTGACTAATGTTG
			2.6-R	TTAACCAAAAGAGGAGTAATTGTAT
	RACK1B	pET28a pGreenII 62-SK pGS-21a pHB	R1B-F	ATGGCTGAAGGACTCGTGTTG
			R1B-R	CTAGTAACGACCAATACCCCAAAC
Site-directed mutagensis	SCE1A	pMD19	K15R-F	GCTGAAGAGAGGAGATCGTGGAGGAAGAATC
			K15R-R	CTCCTCTCTTCAGCTAAACGACC
			C94S-F	CCATCTGGAACTGTCAGTCTCTCTATCCTT
			C94S-R	TGACAGTTCCAGATGGATAGACATTAG
Truncated RAP2.6	RAP2.6	pET28a	Δ1-F	ATGGTGTCTATGCTGACTAATGTTG
			Δ1-R	TTATTTTGGCCTCTCCAAGGACA
			AP2-F	ATGAAATATAGAGGAGTAAGGCAACGAC
			AP2-R	TTAAATCGTCTGAGTTCCAACATTT
			Δ2-F	ATGGTGTCTATGCTGACTAATGTTG
			Δ2-R	TTATGTCGCCTTGTGTGGGTCT
			Δ3-F	ATGGTGTCTATGCTGACTAATGTTG
			Δ3-R	TTAAATCGTCTGAGTTCCAACATTT
